# Supplementary material for: CLASP Suppresses Microtubule Catastrophes through a Single TOG Domain
Source: Dev Cell. 2018 Jul 2;46(1):40–58.e8. doi: 10.1016/j.devcel.2018.05.032 (PMC6035287; doi:10.1016/j.devcel.2018.05.032)
Supplement: Document S1. Figures S1–S7 and Table S1 [file mmc1.pdf]

Developmental Cell, Volume *46*

Supplemental Information

CLASP Suppresses Microtubule Catastrophes through a Single TOG Domain

Amol Aher, Maurits Kok, Ashwani Sharma, Ankit Rai, Natacha Olieric, Ruddi Rodriguez-Garcia, Eugene A. Katrukha, Tobias Weinert, Vincent Olieric, Lukas C. Kapitein, Michel O. Steinmetz, Marileen Dogterom, and Anna Akhmanova

Figure S1

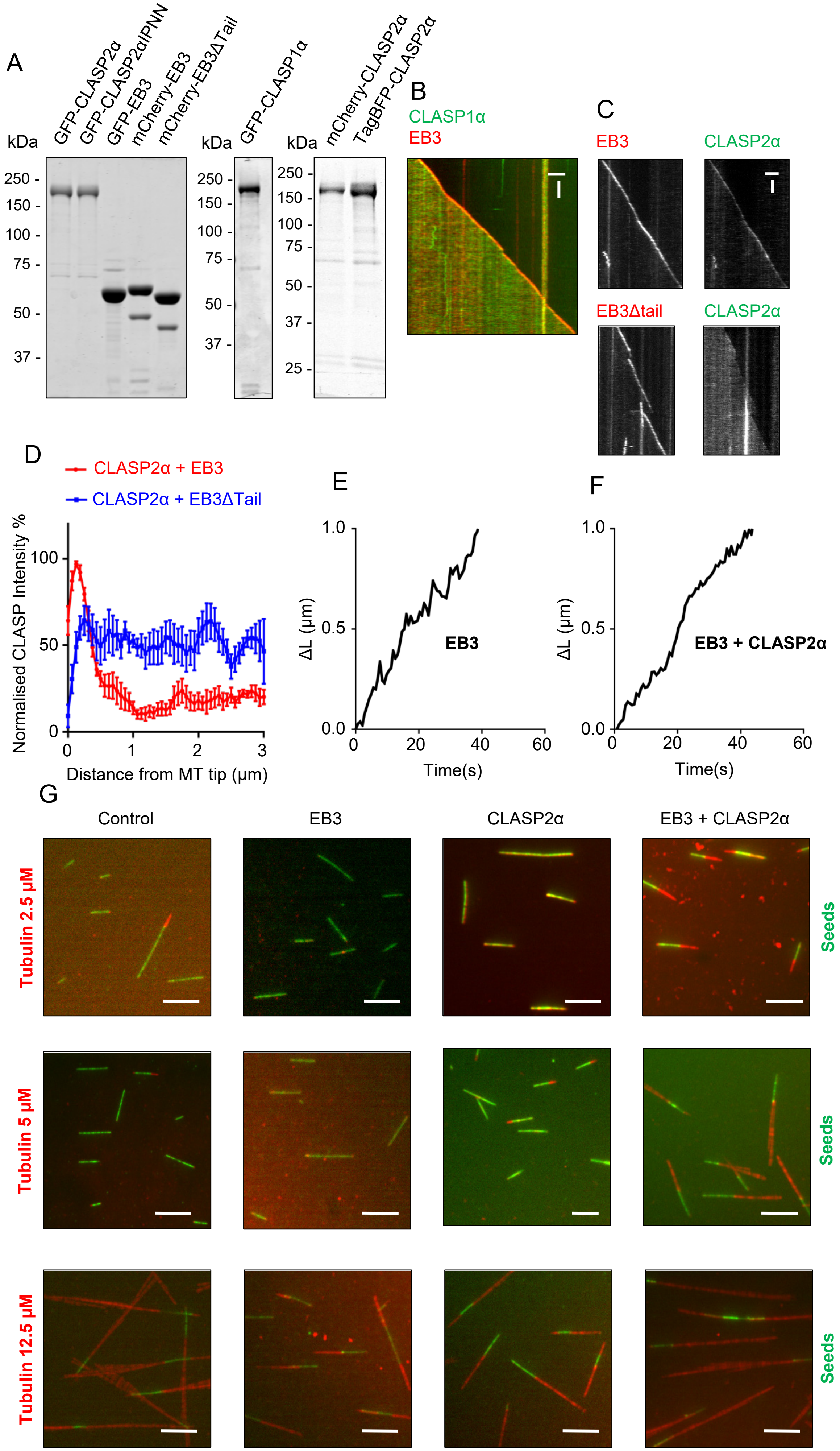
A
GFP-CLASP2α
GFP-CLASP2αIPNN
GFP-EB3
mCherry-EB3
mCherry-EB3ΔTail
GFP-CLASP1α
mCherry-CLASP2α
TagBFP-CLASP2α
kDa
250
150
100
75
50
37
25
B
CLASP1α
EB3
C
EB3
CLASP2α
EB3Δtail
CLASP2α
D
CLASP2α + EB3
CLASP2α + EB3ΔTail
Normalised CLASP Intensity %
Distance from MT tip (μm)
E
EB3
ΔL (μm)
Time(s)
F
EB3 + CLASP2α
ΔL (μm)
Time(s)
G
Control
EB3
CLASP2α
EB3 + CLASP2α
Tubulin 2.5 μM
Tubulin 5 μM
Tubulin 12.5 μM
Seeds
Seeds
Seeds

Figure S1, related to Figure 1. CLASPs suppress catastrophes and promote MT outgrowth from GMPCPP-stabilized seeds.

(A) Coomassie blue stained gels with GFP, mCherry and TagBFP-fusions of CLASP2α, GFP-fusions of the SxIP mutant CLASP2α, EB3 and mCherry-fusions of EB3 and EB3ΔTail. All CLASP proteins were purified from HEK293T cells whereas the EB3 proteins were purified from *E. coli*.

(B) Kymograph showing MT plus end dynamics in the presence of 20 nM mCherry-EB3 and 30 nM GFP-CLASP1α. Scale bars: 2 μm (horizontal) and 60 s (vertical).

(C, D) Kymographs illustrating MT plus end growth for MTs grown in the presence of 30 nM GFP-CLASP2α and 20 nM mCherry-EB3 or 300 nM GFP-CLASP2α and 20 nM mCherry-EB3Δtail (C) and normalized mean fluorescence intensity profiles along a growing MT plus end in the CLASP channel in the same conditions (D). Scale bars: 2 μm (horizontal) and 30 s (vertical). 8 MT profiles were used for averaging.

(E, F) Individual time traces of MT tip position in the presence of 20 nM mCherry-EB3 alone (E) or together with 30 nM GFP-CLASP2α (F).

(G) Maximum intensity projections of MT outgrowth (red) from HiLyte488-labeled GMPCPP seeds (green) in the presence of 2.5 μM, 5 μM and 12.5 μM rhodamine-labeled tubulin alone or together with 200 nM GFP-EB3 or 100 nM GFP-CLASP2α or both. Scale bar: 5 μm.

Figure S2

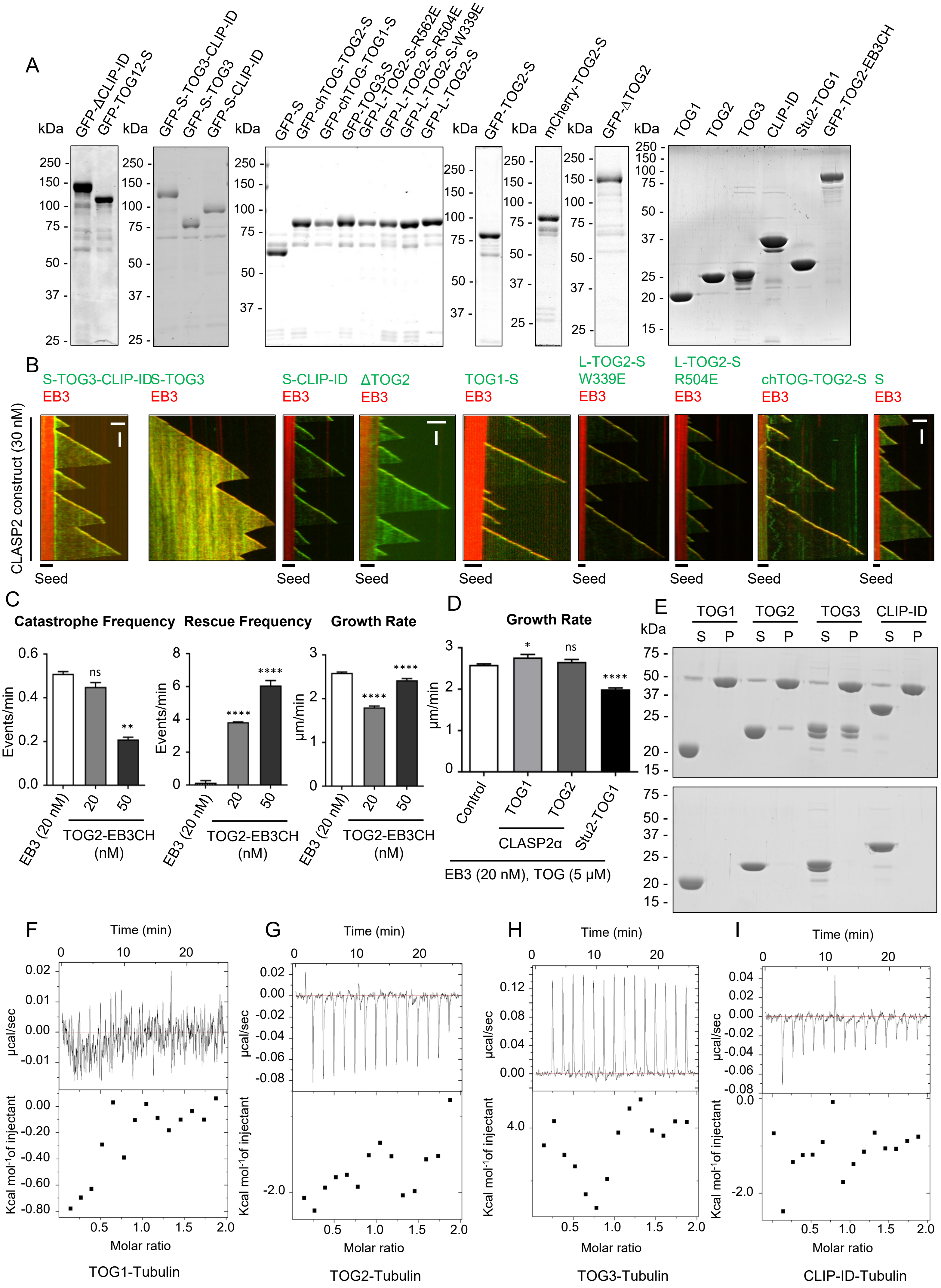

A
GFP-ΔCLIP-ID
GFP-TOG12-S
GFP-S-TOG3-CLIP-ID
GFP-S-TOG3
GFP-S-CLIP-ID
GFP-S
GFP-chTOG-TOG2-S
GFP-chTOG-TOG1-S
GFP-TOG3-S
GFP-L-TOG2-S-R562E
GFP-L-TOG2-S-R504E
GFP-L-TOG2-S-W339E
GFP-L-TOG2-S
GFP-TOG2-S
mCherry-TOG2-S
GFP-ΔTOG2
TOG1
TOG2
TOG3
CLIP-ID
Stu2-TOG1
GFP-TOG2-EB3CH
kDa
B
S-TOG3-CLIP-ID
S-TOG3
S-CLIP-ID
ΔTOG2
TOG1-S
L-TOG2-S W339E
L-TOG2-S R504E
chTOG-TOG2-S
S
EB3
CLASP2 construct (30 nM)
Seed
C
Catastrophe Frequency
Rescue Frequency
Growth Rate
Events/min
μm/min
EB3 (20 nM)
TOG2-EB3CH (nM)
ns
D
Growth Rate
Control
TOG1
TOG2
CLASP2α
Stu2-TOG1
EB3 (20 nM), TOG (5 μM)
E
TOG1
TOG2
TOG3
CLIP-ID
S
P
F
G
H
I
Time (min)
μcal/sec
Kcal mol-1of injectant
Molar ratio
TOG1-Tubulin
TOG2-Tubulin
TOG3-Tubulin
CLIP-ID-Tubulin

Figure S2, related to Figure 2. TOG2 domain of CLASP2α is necessary and sufficient for catastrophe suppression.

(A) Coomassie blue stained gels with GFP-fusions of full-length CLASP2α, TOG domain deletions, SxIP-fusions of different individual TOG domains, point mutants or chimeras with SxIP of CLASP2α fused to chTOG-TOG domains and mCherry-fusion of TOG2-S purified from HEK293T cells and the individual TOG domains of CLASP2α, STU2-TOG1 and the GFP-fusion of CLASP2α-TOG2 domain fused to EB3-CH domain purified from *E. coli*.

(B) Kymographs showing MT plus end dynamics in the presence of 20 nM mCherry-EB3 and 30 nM GFP-fusions of the indicated CLASP2α-TOG domain combinations, point mutants or SxIP motif of CLASP2α fused to chTOG-TOG domain or alone. Scale bars: 2 μm (horizontal) and 60 s (vertical).

(C) Parameters of MT plus end dynamics in the presence of mCherry-EB3 or GFP-TOG2-EB3CH at the indicated concentrations. Number of growth events analyzed: mCherry-EB3 alone, n= 207, GFP-TOG2-EB3CH (20 nM), n=194, GFP-TOG2-EB3CH (50 nM), n=173. Error bars represent SEM. For all plots, **$p<0.05$, ****$p<0.0001$ and ns, no significant difference with control, Mann-Whitney U test.

(D) MT plus end growth rates in the presence of 20 nM mCherry-EB3 alone or together with 5 μM of CLASP2α-TOG1 or TOG2 domains or with Stu2-TOG1 domain. Error bars represent SEM. *$p<0.05$, ****$p<0.0001$ and ns, no significant difference with control, Mann-Whitney U test.

(E) MT pelleting assays with CLASP2α-TOG1, TOG2, TOG3 and the CLIP-interacting domains with taxol-stabilized MTs.

(F-I) ITC analysis of the interaction between tubulin and the CLASP2α-TOG1, TOG2, TOG3 or the CLIP-interacting domain. No interactions were detected.

Figure S3

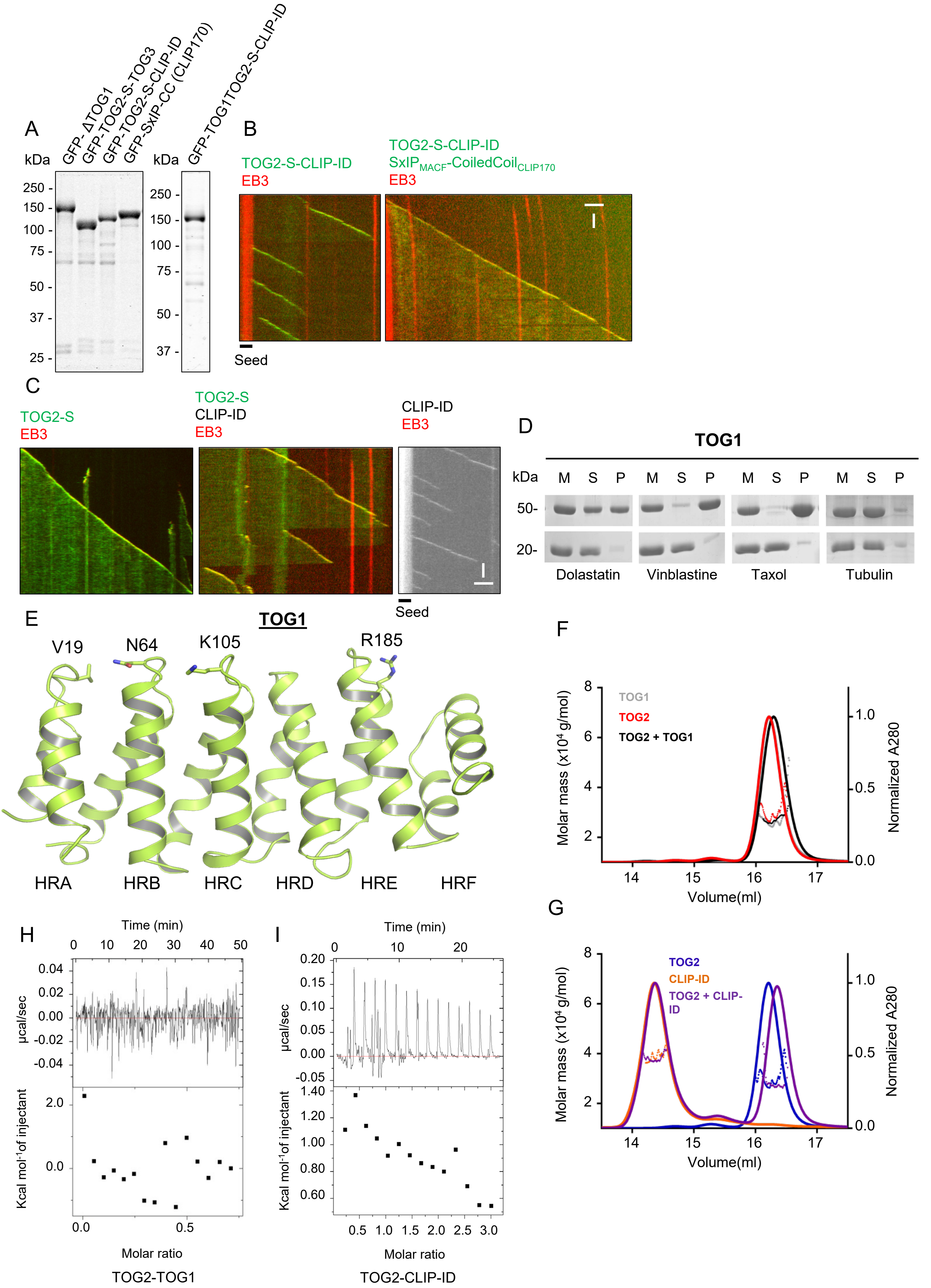

A
kDa
GFP- ΔTOG1
GFP-TOG2-S-TOG3
GFP-TOG2-S-CLIP-ID
GFP-SxIP-CC (CLIP170)
GFP-TOG1TOG2-S-CLIP-ID
250
150
100
75
50
37
25
B
TOG2-S-CLIP-ID
EB3
SxIP$_{MACF}$-CoiledCoil$_{CLIP170}$
Seed
C
TOG2-S
CLIP-ID
EB3
D
TOG1
M S P
50-
20-
Dolastatin
Vinblastine
Taxol
Tubulin
E
TOG1
V19
N64
K105
R185
HRA
HRB
HRC
HRD
HRE
HRF
F
TOG1
TOG2
TOG2 + TOG1
Molar mass (x10^4 g/mol)
Normalized A280
Volume(ml)
G
TOG2
CLIP-ID
TOG2 + CLIP-ID
H
Time (min)
µcal/sec
Kcal mol^{-1}of injectant
Molar ratio
TOG2-TOG1
I
TOG2-CLIP-ID

Figure S3, related to Figure 3. TOG1 domain of CLASP2α has an autoregulatory function and does not interact with MTs or tubulin oligomers.

(A) Coomassie blue stained gels with the indicated GFP-fusions, purified from HEK293T cells.

(B,C) Kymographs showing MT plus end dynamics in the presence of 20 nM mCherry-EB3 and together with the indicated proteins. 30 nM GFP-CLASP fusions, 30 nM of the MACF-CLIP-170 coiled coil fusion and 500 nM CLIP-ID were used. Scale bars: 2 μm (horizontal) and 60 s (vertical).

(D) MT pelleting of CLASP2α-TOG1 domain with tubulin-dolastatin rings, tubulin-vinblastine spirals, taxol-stabilized MTs or tubulin alone, as indicated.

(E) Crystal structure of the CLASP2α-TOG1 domain with the indicated HEAT repeats and the residues at the intra-HEAT loop regions.

(F, G) Analysis of the interactions between tubulin CLASP2α-TOG1 and TOG2, and TOG2 and CLIP-ID domains by size exclusion chromatography followed by multi-angle light scattering (SEC-MALS). No interactions were detected.

(H,I) ITC analysis of the interactions between CLASP2α TOG1 and TOG2 (H), and TOG2 and CLIP-ID (I). No interactions were detected in both the cases.

Figure S4

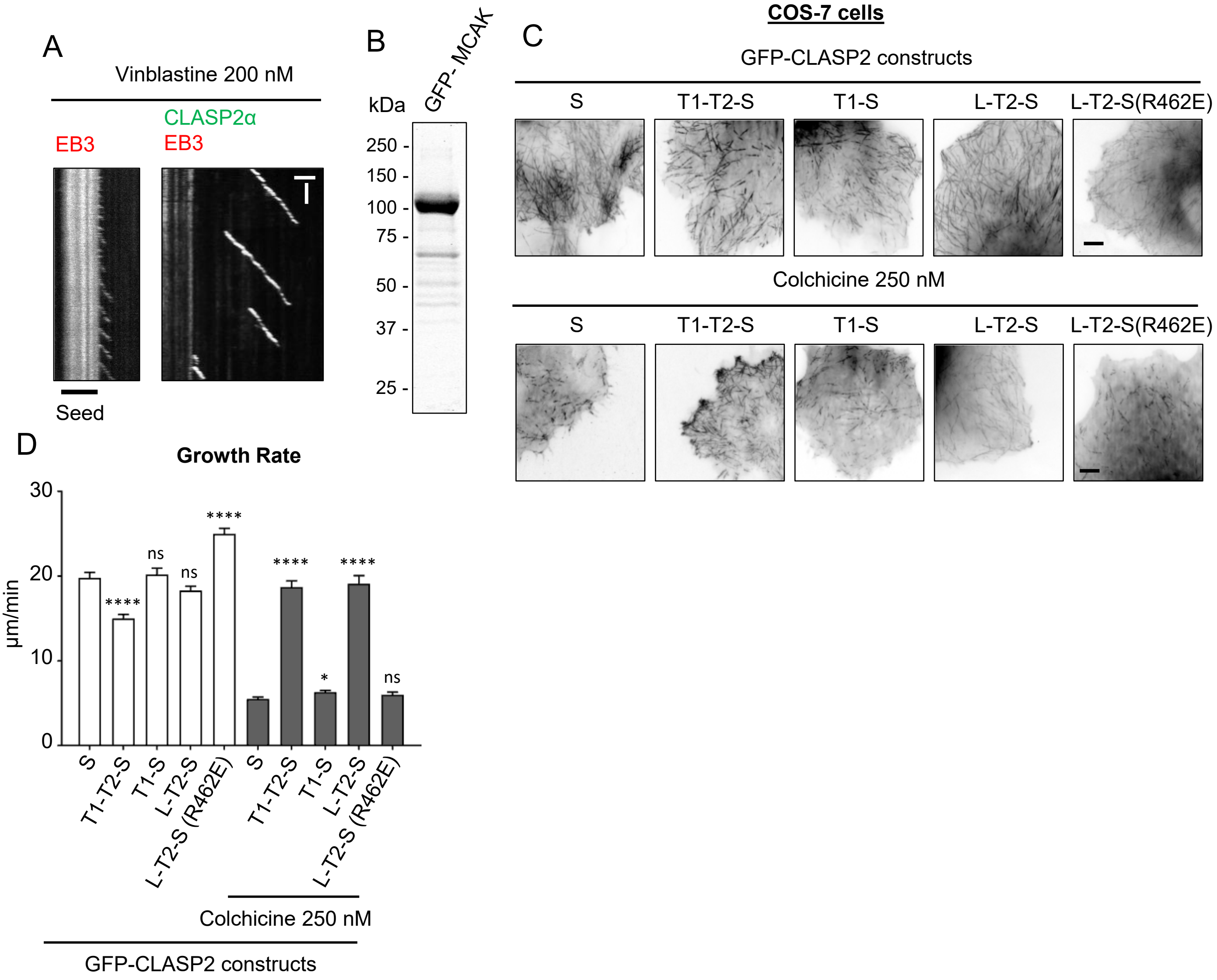
A
Vinblastine 200 nM
EB3
CLASP2α
EB3
Seed
B
GFP- MCAK
kDa
250 -
150 -
100 -
75 -
50 -
37 -
25 -
C
COS-7 cells
GFP-CLASP2 constructs
S
T1-T2-S
T1-S
L-T2-S
L-T2-S(R462E)
Colchicine 250 nM
S
T1-T2-S
T1-S
L-T2-S
L-T2-S(R462E)
D
Growth Rate
µm/min
30
20
10
0
****
ns
ns
****
****
*
****
ns
S
T1-T2-S
T1-S
L-T2-S
L-T2-S (R462E)
S
T1-T2-S
T1-S
L-T2-S
L-T2-S (R462E)
Colchicine 250 nM
GFP-CLASP2 constructs

Figure S4, related to Figure 4. CLASP2α suppresses catastrophes induced by MT-depolymerizing agents.

(A) Kymographs showing MT plus end dynamics in the presence of 20 nM mCherry-EB3 together with either 200 nM vinblastine alone or 30 nM GFP-CLASP2α. Scale bars: 2 μm (horizontal) and 60 s (vertical).

(B) Coomassie blue stained gel with GFP-MCAK.

(C) Maximum intensity projections illustrating time lapse imaging of COS-7 cells expressing GFP fusions of different TOG domain combinations of CLASP2α or a TOG2 point mutant fused to the SxIP motif of CLASP2α; cells were untreated or treated with 250 nM colchicine. Scale bar: 5 μm.

(D) MT plus end growth rates in COS-7 cells shown in (C). *$p<0.05$, ****$p<0.0001$ and ns, no significant difference with control, Mann-Whitney U test.

Figure S5

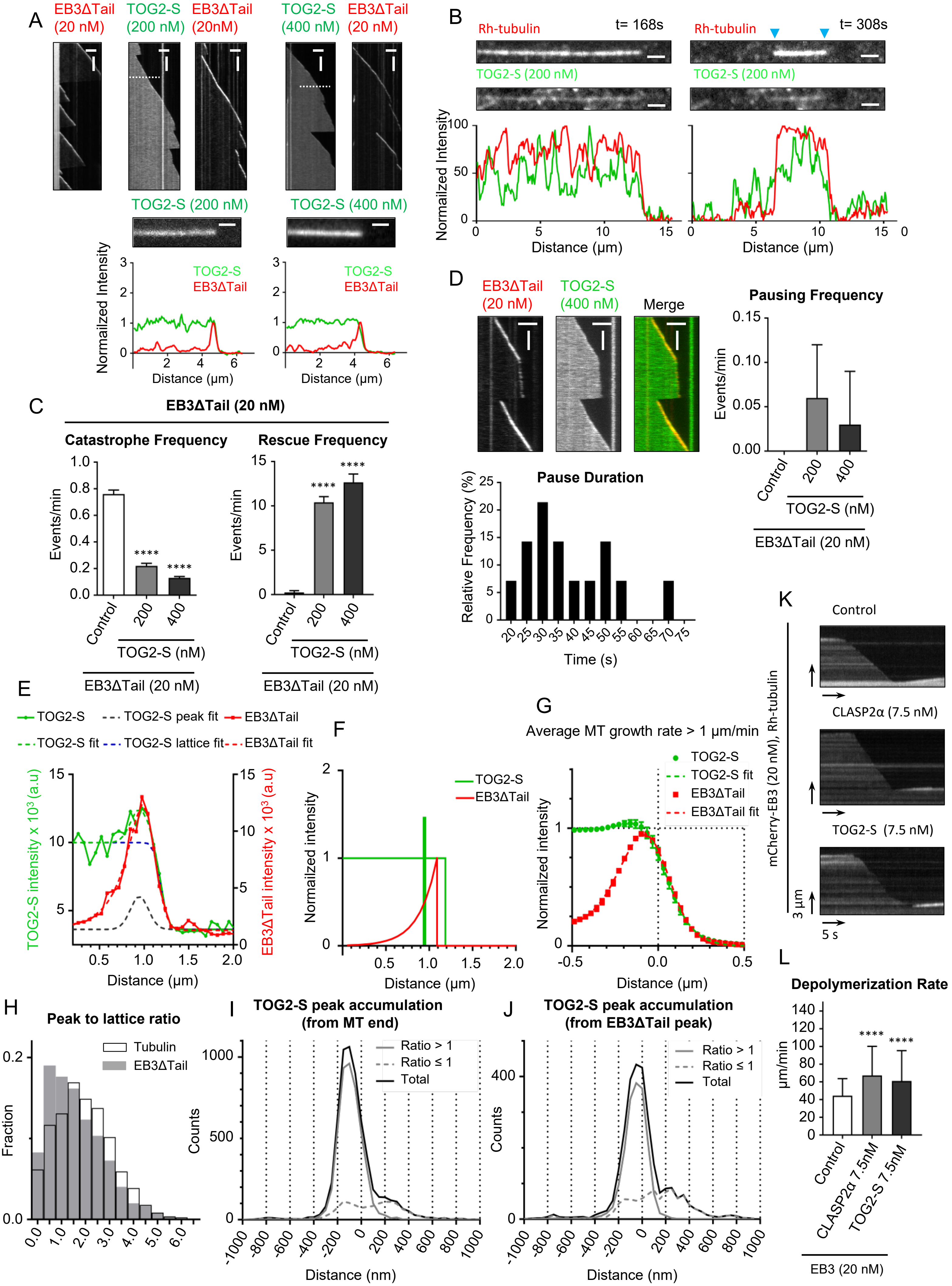

A
EB3ΔTail (20 nM)
TOG2-S (200 nM)
EB3ΔTail (20nM)
TOG2-S (400 nM)
EB3ΔTail (20 nM)
TOG2-S (200 nM)
TOG2-S (400 nM)
Normalized Intensity
Distance (μm)
B
Rh-tubulin
t= 168s
t= 308s
TOG2-S (200 nM)
Normalized Intensity
Distance (μm)
C
EB3ΔTail (20 nM)
Catastrophe Frequency
Rescue Frequency
Events/min
Control
200
400
TOG2-S (nM)
D
EB3ΔTail (20 nM)
TOG2-S (400 nM)
Merge
Pausing Frequency
Pause Duration
Relative Frequency (%)
Time (s)
E
TOG2-S
TOG2-S peak fit
EB3ΔTail
TOG2-S fit
TOG2-S lattice fit
EB3ΔTail fit
TOG2-S intensity x 10³ (a.u)
EB3ΔTail intensity x 10³ (a.u)
Distance (μm)
F
Normalized intensity
G
Average MT growth rate > 1 μm/min
H
Peak to lattice ratio
Tubulin
Fraction
I
TOG2-S peak accumulation (from MT end)
Ratio > 1
Ratio ≤ 1
Total
Counts
Distance (nm)
J
TOG2-S peak accumulation (from EB3ΔTail peak)
K
Control
CLASP2α (7.5 nM)
TOG2-S (7.5 nM)
mCherry-EB3 (20 nM), Rh-tubulin
3 μm
5 s
L
Depolymerization Rate
μm/min
CLASP2α 7.5nM
TOG2-S 7.5nM
EB3 (20 nM)

Figure S5, related to Figure 6. TOG2 shows preference for a MT region behind the outmost MT end.

(A) Kymographs for 20 nM mCherry-EB3ΔTail alone or together with 200 nM GFP-TOG2-S or with 400 nM GFP-TOG2-S, stills and fluorescence intensity profiles for GFP-TOG2-S at the indicated concentrations (200 nM and 400 nM) in the presence of 20 nM mCherry-EB3ΔTail. For stills, scale bar: 1 μm. Scale bars for kymographs: 3 μm (horizontal) and 60 s (vertical).

(B) Stills and fluorescence intensity profiles for GFP-TOG2-S (200 nM) for a microtubule grown in the presence of rhodamine-tubulin post tubulin washout with the time after tubulin washout indicated. Blue arrowheads indicate the depolymerizing MT ends. Scale bar: 2 microns.

(C) Parameters of MT plus end dynamics in the presence of 20 nM mCherry-EB3ΔTail (n=187) in combination with GFP-TOG2-S at 200 nM (n=110) and 400 nM (n=105). Error bars denote SEM. For all plots, ****p<0.0001 and ns, no significant difference with control, Mann-Whitney U test.

(D) Kymographs showing pausing induced by GFP-TOG2-S (400 nM) in the presence of 20 nM mCherry-EB3ΔTail. Scale bar: 2 μm (horizontal) and 30s (vertical). Distributions of the pause durations (200 nM GFP-TOG2-S) are shown in the bottom panel, and the pausing frequency in the presence of 20 nM mCherry-EB3ΔTail alone or in combination with 200nM and 400 nM of GFP-TOG2-S (n=14 and n=9 in a total growth time of 255.5 and 291.9 minutes respectively) is shown in the right panel. Error bars denote SEM.

(E) Example of an individual fitting of GFP-TOG2-S and mCherry-EB3ΔTail fluorescence intensity profiles at GFP-TOG2-S (400 nM) and mCherry-EB3ΔTail (20 nM). Blue dashed line corresponds to the lattice and black dashed line to the peak accumulation components of overall TOG2-S fit function (green dashed line).

(F) Density distribution of TOG2-S (peak and lattice components shown separately, green lines) and EB3ΔTail (exponential decay, red line) extracted from the fitting shown in (E).

(G) Normalized, aligned, resampled and averaged fluorescence intensity profiles of GFP-TOG2-S (400 nM) and mCherry-EB3ΔTail (20 nM) with the average MT growth rate above 1 μm/min. Profiles were first averaged per kymograph. Error bars represent SEM of second averaging among multiple kymographs (10 kymographs, 2413 total time points).

(H) Histograms of ratios of the peak intensity to lattice intensity of GFP-TOG2-S (400 nM) measured on rhodamine-tubulin-labeled MTs (white bars, n=5997) or in the presence of mCherry-EB3ΔTail (20 nM) (grey bars, n=2774 time points).

(I) Histograms of the distances between TOG2-S intensity peak accumulation and the fitted position of MT tip (solid line, n=5997) for 400 nM GFP-TOG2-S. Grey line displays the histogram when TOG2-S tip to lattice intensity ratio is above 1 (n=4586) and dashed grey line when it is below 1 (n=1411). In the histograms, 0 corresponds to the fitted position of the MT end or EB3ΔTail peak.

(J) Histograms of distance between TOG2-S intensity peak accumulation and the fitted peak of EB3ΔTail (solid line, n=2774) for 400 nM GFP-TOG2-S and 20 nM mCherry-EB3Δtail. Grey line displays the histogram when TOG2-S tip to lattice intensity ratio is above 1 (n=1778) and dashed grey line when it is below 1 (n=996). In the histograms, 0 corresponds to the fitted position of the EB3ΔTail peak.

(K,L) Kymographs of depolymerizing MT ends in the presence of rhodamine-tubulin and mCherry-EB3 (20 nM) alone (n=160) or together with 7.5 nM GFP-CLASP2α (n=114) or 7.5 nM GFP-TOG2-S (n=151) (K) and the corresponding quantification of

depolymerization rates (L). Scale bar: 3 μm (vertical) and 5s (horizontal). Error bars denote SD. For depolymerization rates plot, ****$p<0.0001$, Mann-Whitney U test.

Figure S6

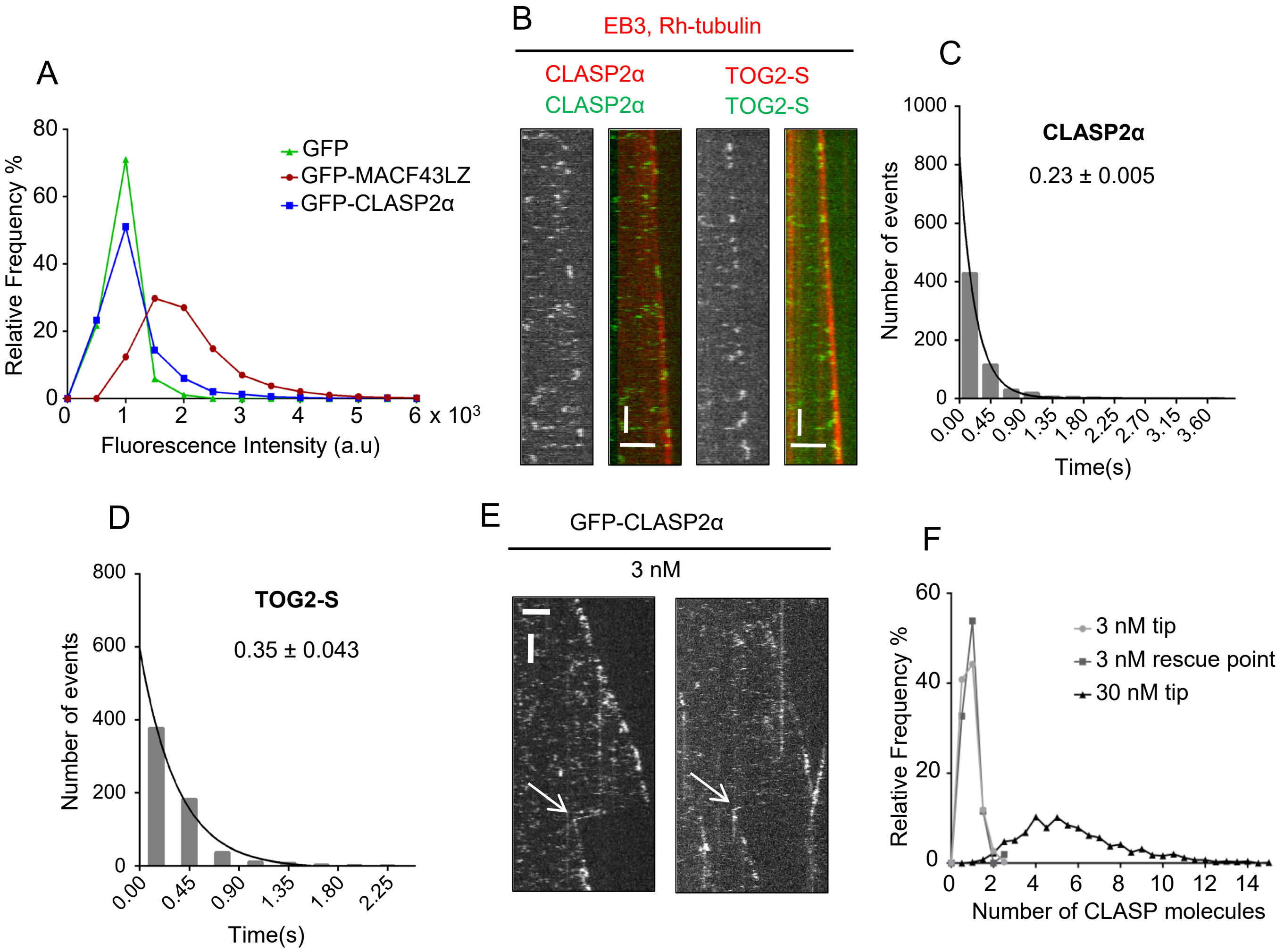

A
Relative Frequency %
Fluorescence Intensity (a.u)
6 x 10³
GFP
GFP-MACF43LZ
GFP-CLASP2α
B
EB3, Rh-tubulin
CLASP2α
CLASP2α
TOG2-S
TOG2-S
C
CLASP2α
0.23 ± 0.005
Number of events
Time(s)
D
TOG2-S
0.35 ± 0.043
Number of events
Time(s)
E
GFP-CLASP2α
3 nM
F
3 nM tip
3 nM rescue point
30 nM tip
Relative Frequency %
Number of CLASP molecules

Figure S6, related to Figure 7. A small number of CLASP2α molecules can suppress catastrophe and induce rescue.

(A) Distributions of fluorescence intensity values for single molecules of GFP (green, mean intensity value=0.92 x 10^3 ± 0.25 x 10^3) (n=846 molecules analyzed), GFP-CLASP2α (blue, mean=1.2 x 10^3 ± 1.3 x 10^3) (n=2341) and GFP-MACF43-LZ (red, mean=2.12 x 10^3 ± 1.5 x 10^3) (n=7516).

(B) Kymographs showing MT plus end and lattice turnover for GFP-CLASP2α and TOG2-S for MTs grown in the presence of 20 nM mCherry-EB3, rhodamine-tubulin together with both 30 nM mCherry-CLASP2α and 0.5 nM GFP-CLASP2α or 30 nM mCherry-TOG2-S and 0.5 nM GFP-TOG2-S. Scale bars: 2 μm (horizontal) and 2 s (vertical).

(C, D) Exponential fits of the distributions of dwell times at the MT tip for CLASP2α (mean time= 0.23 ± 0.005 s), n=608 tip events (C) and TOG2-S (mean time= 0.35 ± 0.043 s), n=622 tip events (D).

(E) Kymographs showing MT plus end dynamics in the presence of 3 nM GFP-CLASP2α and 20 nM mCherry-EB3 (only green channel shown). Scale bars: 2 μm (horizontal) and 30 s (vertical).

(F) Distributions of the number of GFP-CLASP2α molecules in the presence of 20 nM mCherry-EB3 at growing MT plus ends (n=1078 tip events) and at rescue points (n=17 rescue events) in the presence of 3 nM GFP-CLASP2α, and at growing MT plus end with 30 nM GFP-CLASP2α (n=1393 tip events).

Figure S7

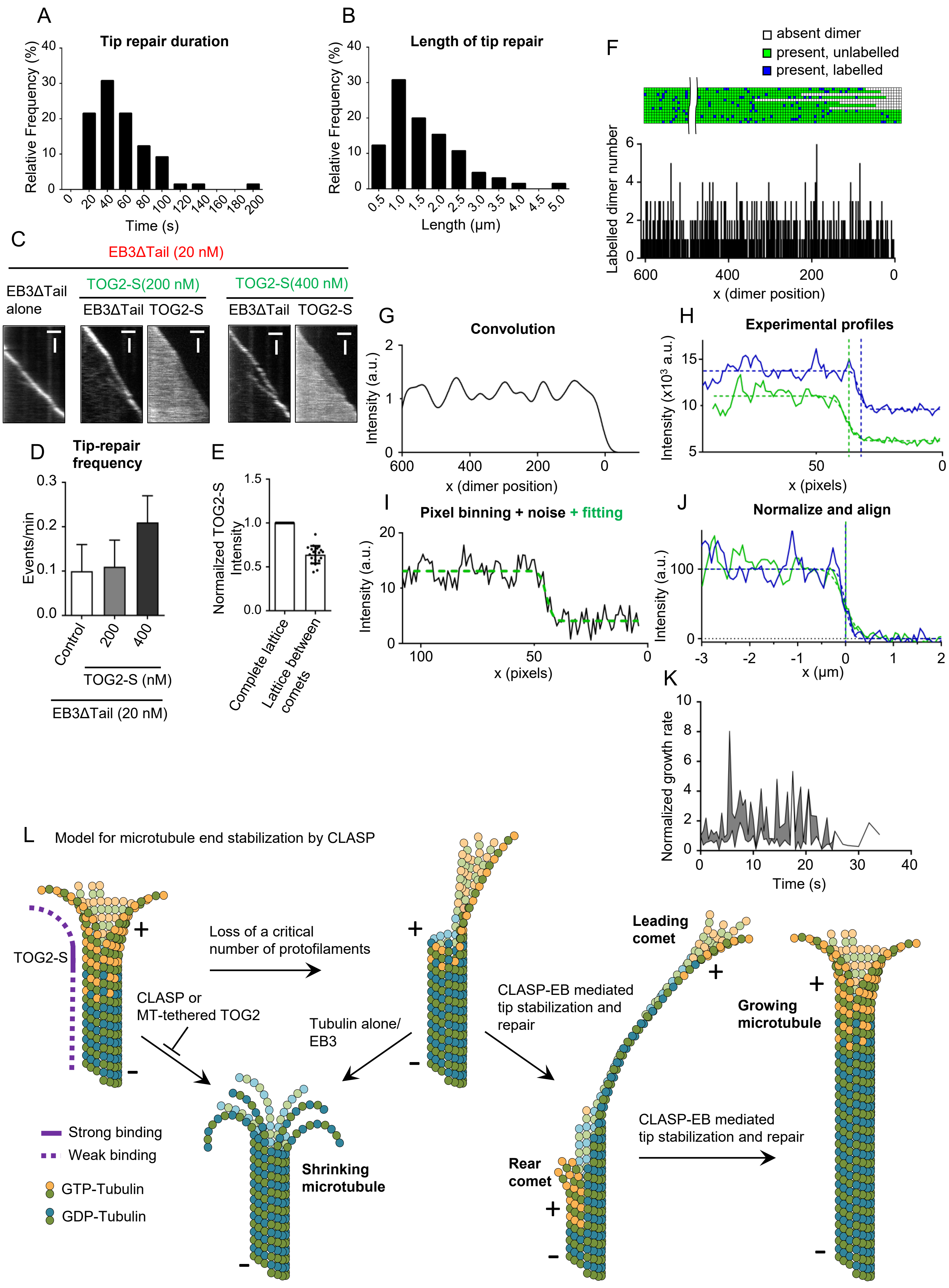

A
Tip repair duration
Relative Frequency (%)
Time (s)
B
Length of tip repair
Relative Frequency (%)
Length (μm)
C
EB3ΔTail (20 nM)
EB3ΔTail alone
TOG2-S(200 nM)
EB3ΔTail TOG2-S
TOG2-S(400 nM)
EB3ΔTail TOG2-S
D
Tip-repair frequency
Events/min
Control
200
400
TOG2-S (nM)
EB3ΔTail (20 nM)
E
Normalized TOG2-S Intensity
Complete lattice
Lattice between comets
F
absent dimer
present, unlabelled
present, labelled
Labelled dimer number
x (dimer position)
G
Convolution
Intensity (a.u.)
x (dimer position)
H
Experimental profiles
Intensity (x10³ a.u.)
x (pixels)
I
Pixel binning + noise + fitting
Intensity (a.u.)
x (pixels)
J
Normalize and align
Intensity (a.u.)
x (μm)
K
Normalized growth rate
Time (s)
L
Model for microtubule end stabilization by CLASP
TOG2-S
Loss of a critical number of protofilaments
CLASP or MT-tethered TOG2
Tubulin alone/ EB3
CLASP-EB mediated tip stabilization and repair
Leading comet
Growing microtubule
Rear comet
Shrinking microtubule
Strong binding
Weak binding
GTP-Tubulin
GDP-Tubulin

Figure S7, related to Figure 7. Characterization of MT tip repair events.

(A, B) Distributions of the duration (A) and length (B) of tip repair events, mean time=54 ± 4 s; mean length= 1.6 ± 0.1 microns from n=65 events. Error bars represent SEM.

(C) Kymographs showing tip repair events with EB3ΔTail alone and together with the indicated concentrations of TOG2-S (200 nM and 400 nM). Scale bars: 1 μm (horizontal) and 15 s (vertical).

(D) Frequency of tip repair for MTs grown in the presence of 20 nM mCherry-EB3ΔTail alone (n=24) or together with 200 nM or 400nM GFP-TOG2-S (n=27 and 62 respectively). The frequency was calculated by dividing the number of observed tip repair events by the total growth time, n=number of tip repair events observed in each condition. Error bars represent SEM.

(E) Averaged fluorescence intensity of GFP-TOG2-S in the MT lattice region between the leading and lagging comet, normalized to the average intensity along the complete MT lattice (n=21). Mean ± SD.

(F) Schematic view of MT structure used for Monte-Carlo simulations and an example of labeled dimer number distribution along MT length for a single random realization of simulation.

(G) Intensity profile after convolution of dimer number density from (F) with the point spread function of a microscope.

(H) Examples of experimentally measured MT tip intensity profiles (solid lines) and corresponding error function fittings (dashed lines), vertical lines mark a position of tip derived from the fitting.

(I) Intensity profile from (G) after binning in x with the experimental image pixel size and noise addition (black solid line) and corresponding error function fitting (green dashed line).

(J) Experimental intensity profiles from (H) (solid lines) and corresponding fitted curves (dashed lines) after background subtraction, alignment and normalization.

(K) Quantification of rear comet velocities over time during tip repair events corresponding to Fig 7N. Grey areas represent SEM.

(L) Model for MT end stabilization mediated by CLASP.

Table S1, related to Figure 3, S3. X ray crystallography data collection and refinement statistics.

**Data Collection**[a]	**Native**	**S-SAD**
Wavelength (Å)	1	2.066
Resolution range (Å)[b]	38.49 - 1.19 (1.24 - 1.19)	47.12 - 2.14 (2.2 - 2.14)
Space group	P 1 21 1	P 1 21 1
Unit cell a, b, c (Å) α, β, γ (°)	66.08, 47.12, 74.15, 90, 115.83, 90	66.05, 47.12, 74.02, 90, 115.797, 90
Total reflections	797853 (62543)	831552 (6204)
Unique reflections	128081 (11920)	21534 (1379)
Multiplicity	6.2 (5.1)	38.62 (4.50)
Completeness (%)	99 (95)	93.49 (60.91)
Mean I/sigma(I)	13.42 (0.84)	64.0 (10.2)
Wilson B-factor	12.78	-
R-merge	0.07116 (1.571)	0.056 (0.119)
R-meas	0.07762 (1.748)	0.056 (0.132)
CC1/2[c]	1 (0.3)	0.999 (0.984)
CC*	1 (0.679)	Chi orientations/oscillations :<br>Chi0/360°<br>Chi5/360°<br>Chi10/360°<br>Chi15/360°<br>Chi20/360°<br>Chi25/360°<br>Chi30/360°<br>Chi35/360°
**Refinement**		
R-work	0.2003 (0.2917)	
R-free	0.2137 (0.3135)	
Macromolecules	3368	
Water	770	
RMS(bonds) (Å)	0.008	
RMS(angles) (°)	1.27	
Ramachandran favored (%)[d]	99	
Ramachandran outliers (%)[d]	0	
**B-factors**		
Average B-factor	21.22	
Macromolecules	19.03	

Solvent	30.80

[a] Highest resolution shell statistics are in parentheses.
[b] Resolution cutoffs were chosen based on CC1/2 and Mean I/sigma(I) (Karplus and Diederichs, 2012)
[c] As defined by (Karplus and Diederichs, 2012)
[d] As defined by MolProbity (Davis et al., 2004)
