# Supplementary material for: CLASP Suppresses Microtubule Catastrophes through a Single TOG Domain
Source: Dev Cell. 2018 Jul 2;46(1):40–58.e8. doi: 10.1016/j.devcel.2018.05.032 (PMC6035287; doi:10.1016/j.devcel.2018.05.032)
Supplement: Document S2. Article plus Supplemental Information [file mmc7.pdf]

Developmental Cell

Article

CLASP Suppresses Microtubule Catastrophes through a Single TOG Domain

Graphical Abstract

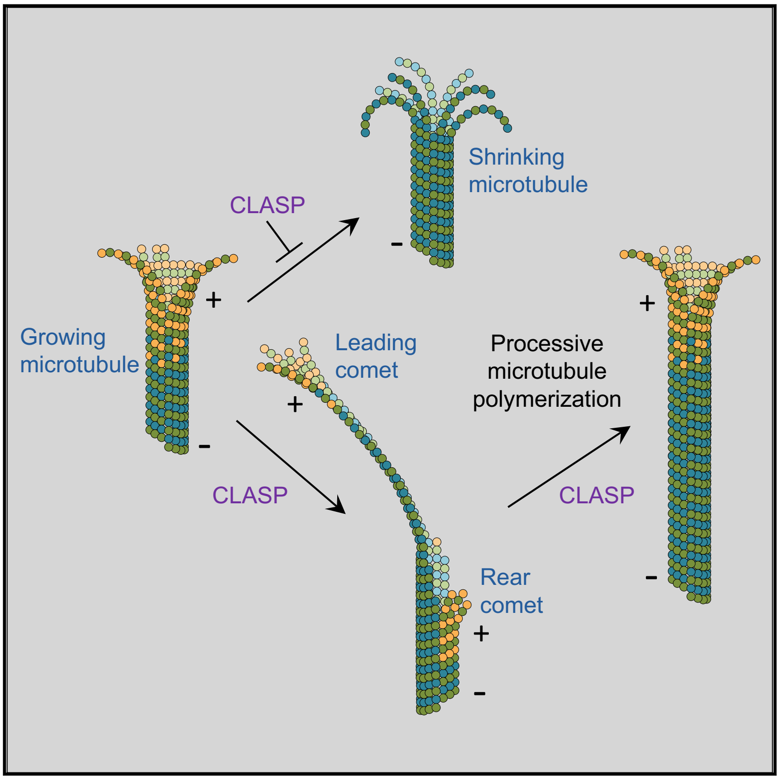

Highlights

- CLASPs potently suppress microtubule catastrophes induced by different mechanisms
- CLASPs act by stabilizing growing microtubule ends, including incomplete ones
- CLASP2 TOG-like domain, TOG2, is necessary and sufficient for catastrophe inhibition
- TOG2 fused to a positively charged peptide accumulates at growing microtubule ends

Authors

Amol Aher, Maurits Kok, Ashwani Sharma, ..., Michel O. Steinmetz, Marileen Dogterom, Anna Akhmanova

Correspondence

a.akhmanova@uu.nl

In Brief

Aher et al. dissect the mechanisms underlying the ability of CLASPs, major microtubule-stabilizing factors in interphase and mitosis, to prevent microtubule from switching from growth to shortening. They show that the CLASP domain essential for this function does not bind to free tubulin but directly stabilizes growing microtubule ends.

Aher et al., 2018, Developmental Cell *46*, 40–58
July 2, 2018 © 2018 The Authors. Published by Elsevier Inc.
https://doi.org/10.1016/j.devcel.2018.05.032

Developmental Cell
Article

CLASP Suppresses Microtubule Catastrophes through a Single TOG Domain

Amol Aher,[1] Maurits Kok,[2] Ashwani Sharma,[3] Ankit Rai,[1] Natacha Olieric,[3] Ruddi Rodriguez-Garcia,[1] Eugene A. Katrukha,[1] Tobias Weinert,[3] Vincent Olieric,[4] Lukas C. Kapitein,[1] Michel O. Steinmetz,[3,5] Marileen Dogterom,[2] and Anna Akhmanova[1,6,*]

[1]Cell Biology, Department of Biology, Faculty of Science, Utrecht University, Padualaan 8, 3584 CH Utrecht, the Netherlands
[2]Department of Bionanoscience, Kavli Institute of Nanoscience, Delft University of Technology, van der Maasweg 9, 2629 HZ Delft, the Netherlands
[3]Laboratory of Biomolecular Research, Division of Biology and Chemistry, Paul Scherrer Institut, CH-5232 Villigen PSI, Switzerland
[4]Swiss Light Source, Paul Scherrer Institut, CH-5232 Villigen PSI, Switzerland
[5]University of Basel, Biozentrum, 4056 Basel, Switzerland
[6]Lead Contact
*Correspondence: a.akhmanova@uu.nl
https://doi.org/10.1016/j.devcel.2018.05.032

SUMMARY

The dynamic instability of microtubules plays a key role in controlling their organization and function, but the cellular mechanisms regulating this process are poorly understood. Here, we show that cytoplasmic linker-associated proteins (CLASPs) suppress transitions from microtubule growth to shortening, termed catastrophes, including those induced by microtubule-destabilizing agents and physical barriers. Mammalian CLASPs encompass three TOG-like domains, TOG1, TOG2, and TOG3, none of which bind to free tubulin. TOG2 is essential for catastrophe suppression, whereas TOG3 mildly enhances rescues but cannot suppress catastrophes. These functions are inhibited by the C-terminal domain of CLASP2, while the TOG1 domain can release this auto-inhibition. TOG2 fused to a positively charged microtubule-binding peptide autonomously accumulates at growing but not shrinking ends, suppresses catastrophes, and stimulates rescues. CLASPs suppress catastrophes by stabilizing growing microtubule ends, including incomplete ones, preventing their depolymerization and promoting their recovery into complete tubes. TOG2 domain is the key determinant of these activities.

INTRODUCTION

Microtubules (MTs) are dynamic cytoskeletal polymers composed of tubulin dimers, which attach to each other in a head-to-tail fashion to form protofilaments that interact laterally to form a hollow tube (Desai and Mitchison, 1997). MTs can alternate between phases of growth, shortening, and pause, and numerous cellular factors regulate these transitions (Akhmanova and Steinmetz, 2015; Mimori-Kiyosue, 2011). Switching to MT depolymerization, termed catastrophe, can be induced by intrinsic MT properties, such as fluctuations in the size of the protective guanosine triphosphate (GTP) cap (Brouhard, 2015; Howard and Hyman, 2009). When MTs are grown *in vitro* in a system without boundaries, the catastrophe frequency increases with MT age (Gardner et al., 2011b). Recent work suggested that this behavior could be explained by MT end tapering, which would affect tubulin binding/unbinding kinetics (Coombes et al., 2013) or the density of the protective cap close to the MT end (Duellberg et al., 2016b). Furthermore, MT catastrophes can be induced by MT depolymerases that can cause protofilament peeling, by an encounter with obstacles that block protofilament elongation or by MT-destabilizing agents that can induce structural defects at MT tips (Akhmanova and Steinmetz, 2015; Gardner et al., 2013). Interestingly, blocking just one MT protofilament at the growing MT end can disrupt growth and induce a catastrophe (Doodhi et al., 2016), but it is unclear how severe the accompanying aberrations in MT structure can be, and whether and how they can be repaired.

Cytoplasmic linker-associated proteins (CLASPs) are excellent candidates to promote MT growth because they are well known to increase MT abundance and stability in mitosis and interphase. Mammalian CLASPs are essential for proper spindle MT dynamics and MT polymerization near kinetochores (Maiato et al., 2003, 2005), and the depletion of CLASPs leads to severe spindle defects (Maiato et al., 2003). CLASP homologs stabilize overlapping MTs in mitotic spindles of fission yeast (Bratman and Chang, 2007) and induce MT pausing in *Drosophila* S2 cells (Sousa et al., 2007). In worms, CLASPs are required for the assembly of the central spindle in embryos (Maton et al., 2015) and suppress catastrophes in muscle cells (Lacroix et al., 2014). In plants, CLASPs inhibit catastrophes when MTs grow around sharp cell edges (Ambrose et al., 2011). In migrating mammalian cells, CLASPs stimulate MT rescues (switches from shrinkage to growth) at leading cell edges in 2D (Mimori-Kiyosue et al., 2005) and inhibit catastrophes at the tips of mesenchymal cell protrusions in a 3D matrix (Bouchet et al., 2016). Moreover, CLASPs can promote γ-tubulin-dependent MT nucleation at the Golgi (Efimov et al., 2007).

The ability of CLASPs to induce MT rescues, inhibit catastrophes, and induce pausing has been reconstituted *in vitro* (Al-Bassam et al., 2010; Moriwaki and Goshima, 2016; Yu et al., 2016). Different CLASP homologs contain two or three TOG-like

 © 2018 The Authors. Published by Elsevier Inc.
This is an open access article under the CC BY license (http://creativecommons.org/licenses/by/4.0/).

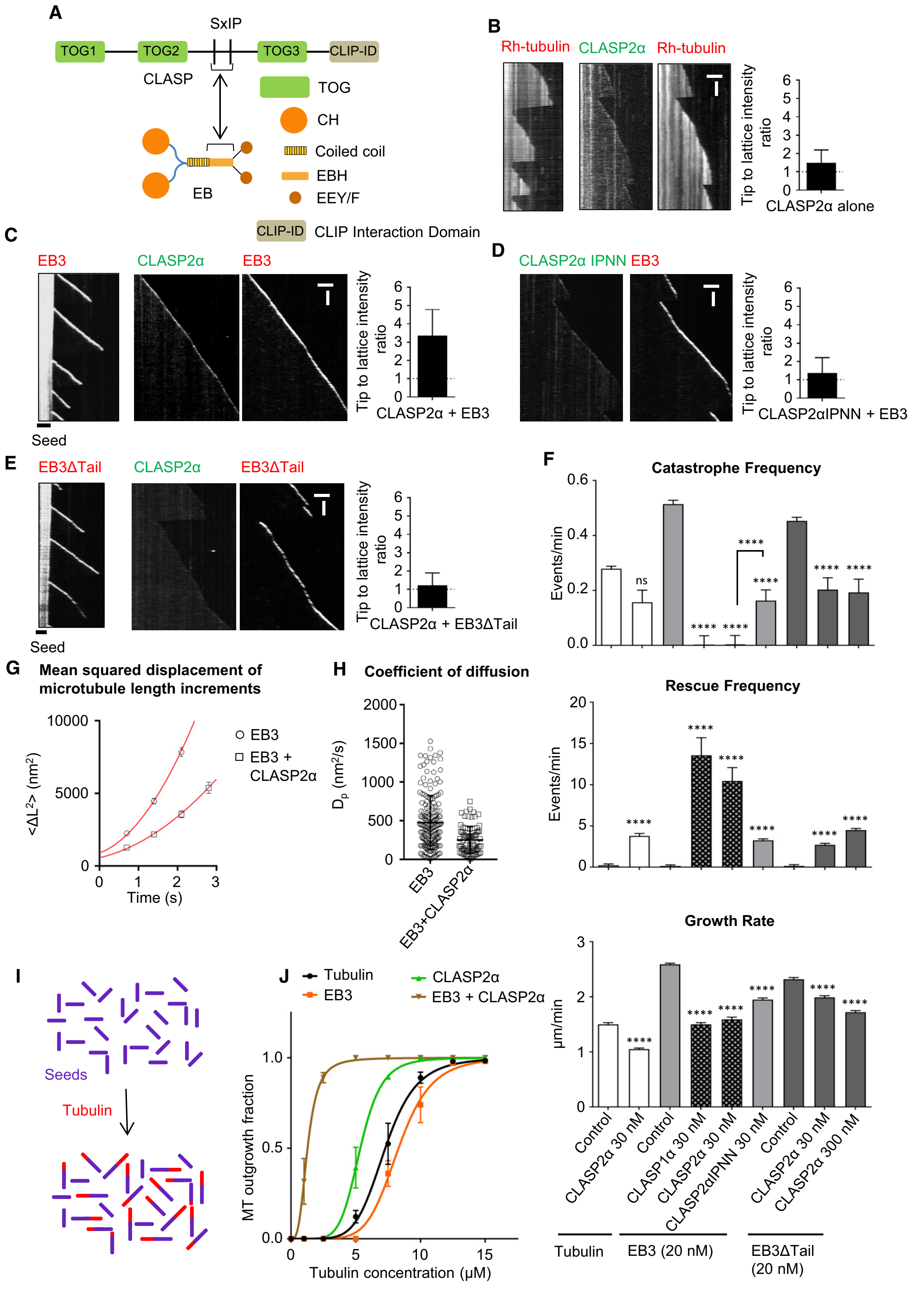

(legend on next page)

domains, protein modules known to bind to free tubulin, and it has been proposed that CLASPs act like MT polymerases by promoting the recruitment of tubulin dimers (Al-Bassam et al., 2010; Yu et al., 2016). However, unlike the TOG domain-containing MT polymerases of the XMAP215/ch-TOG family, CLASPs do not accelerate MT growth but either slow it down or do not affect it (Lawrence et al., 2018; Moriwaki and Goshima, 2016; Yu et al., 2016), and the known structures of CLASP TOG-like domains are incompatible with binding to free tubulin due to their highly convex architecture (Leano et al., 2013; Maki et al., 2015). Alternatively, it was proposed that CLASPs might affect MTs by binding to highly curved protofilaments at MT ends (Maki et al., 2015), but this has not been directly tested. The mechanisms by which CLASPs stabilize MT growth and prevent depolymerization thus remain unresolved.

Here, by using *in vitro* MT dynamics assays, we show that CLASPs potently suppress MT catastrophes that occur spontaneously or are induced by MT-destabilizing agents and physical barriers and promote templated MT nucleation. We demonstrate that a single TOG-like domain of CLASP2, TOG2, which does not bind to free tubulin, is sufficient to induce rescues and, when targeted to MT plus ends, suppress catastrophes. Another TOG-like CLASP2 domain, TOG3, can promote rescues but does not inhibit catastrophes. The additional folded domains present in CLASPs do not bind to free tubulin or MTs but rather have autoregulatory and partner-binding functions. Furthermore, we show that CLASP2 stabilizes incomplete MT structures at the plus ends, thereby enabling their restoration to promote processive MT growth. We find that TOG2 is essential and, when recruited to the MT plus end, sufficient for these functions. When tethered to MTs through a positively charged peptide, TOG2 autonomously accumulates at the growing but not depolymerizing MT ends and is enriched in a region behind the outmost tip that likely overlaps with the GTP (or GDP-Pi) cap. Taken together, our data suggest that TOG2 acts by preventing the loss of the stabilizing cap associated with MT growth.

RESULTS

A Complex of CLASP2α and EB3 Suppresses Catastrophes and Promotes Templated MT Nucleation

To investigate the impact of CLASP2α on MT dynamics, we purified it from HEK293T cells (Figure S1A) and analyzed its activity using an *in vitro* reconstitution assay (Bieling et al., 2007; Doodhi et al., 2016), in which MT growth from GMPCPP-stabilized seeds is observed by total internal reflection fluorescence microscopy (TIRFM). In the presence of tubulin alone, full-length GFP-tagged CLASP2α showed some binding to MT lattices and a very weak enrichment at MT tips (Figures 1A and 1B). However, when mCherry-EB3 was included in the assay, CLASP2α strongly accumulated at MT plus ends (Figures 1A and 1C). MT tip recruitment of CLASP2α was abrogated by mutating the Ile and Pro residues of the two tandemly arranged SxIP motifs in the middle of the protein to asparagines (IPNN mutant) or by removal of the acidic tail of EB3 (EB3ΔTail), as these polypeptide sequences are essential for the binding between CLASP2 and EBs (Honnappa et al., 2009) (Figures1D, 1E, and S1A).

Analysis of MT dynamics showed that 30 nM CLASP2α had a mild inhibitory effect on the MT growth rate both with (1.6-fold reduction) and without EB3 (1.4-fold reduction) (Figure 1F). Strikingly, when recruited to MT tips by EB3, CLASP2α almost completely suppressed catastrophes (Figures 1C and 1F). CLASP1α, the CLASP2α paralog that shares a very similar domain organization, displayed a very similar activity (Figures 1F, S1A, and S1B). Catastrophe suppression was not observed when EB3 was absent or when the binding between CLASP2α and EB3 was abolished (Figures 1B–1F). We next attempted to compensate for the lack of interaction between CLASP2α and EB3 by increasing the concentration of CLASP2α from 30 to

Figure 1. CLASP2α Promotes Processive MT Polymerization and MT Outgrowth from a Template

(A) A scheme of CLASP and EB domain organization and CLASP-EB interaction.

(B–E) Kymographs of MT plus end growth with rhodamine-tubulin alone or together with 30 nM GFP-CLASP2α (B), 20 nM mCherry-EB3 alone or together with 30 nM GFP-CLASP2α (C), 20 nM mCherry-EB3 and 30 nM GFP-CLASP2αIPNN (D), and 20 nM mCherry-EB3ΔTail alone or together with 30 nM GFP-CLASP2α (E). Plots of fluorescence intensity ratio of CLASP2α at the growing MT plus end and MT lattice are shown on the right, n = 27 (B), 26 (C), 25 (D), and 30 (E). Scale bars, 2 μm (horizontal) and 60 s (vertical).

(F) Parameters of MT plus end dynamics in the presence of rhodamine-tubulin alone or together with 20 nM mCherry-EB3 or together with 20 nM mCherry-EB3ΔTail in combination with the indicated CLASP constructs at 30 or 300 nM as indicated. Number of growth events analyzed: for tubulin alone, n = 135, tubulin with GFP-CLASP2α, n = 134, mCherry-EB3 alone, n = 207, mCherry-EB3 with GFP-CLASP1α, n = 110, mCherry-EB3 with GFP-CLASP2α, n = 110, mCherry-EB3 with GFP-CLASP2αIPNN, n = 182, mCherry-EB3ΔTail, n = 182, mCherry-EB3ΔTail and GFP-CLASP2α, n = 174, mCherry-EB3ΔTail and 300 nM GFP-CLASP2α, n = 128. Error bars represent SEM.

(G and H) Average of the mean-squared displacement (MSD) of MT length increments, plotted over time (G) and the values of the diffusion constant D_p, obtained from fits of the MSD curves (H). Data are shown for MTs grown either in the presence of EB3 alone or together with 30 nM of CLASP2α. The average diffusion constant of 506 ± 41 nm^2/s for control and 316 ± 25 nm^2/s in presence of CLASP2α were estimated from fits to the data (red line). Each dot in (H) represents the diffusion constant estimated for an individual MT growth event; control (n = 183), CLASP2α (n = 88).

(I and J) Schematic of the MT outgrowth assay and plot of the fraction of the total GMPCPP seeds that show MT outgrowth in 15 min at increasing tubulin concentrations with tubulin alone (black) or together with GFP-EB3 (200 nM) (orange), or together with GFP-CLASP2α (100 nM) (green), or together with GFP-EB3 (200 nM) and GFP-CLASP2α (100 nM) (brown). For increasing tubulin concentrations in the case of tubulin alone, n = 92, 96, 105, 82, 97, 87, 161, and 127 GMPCPP seeds, respectively, for 200 nM GFP-EB3, n = 69, 73, 68, 77, 80, 83, 106, and 96 GMPCPP seeds, respectively, for 100 nM GFP-CLASP2α, n = 119, 122, 118, 119, 145, 110, 119, and 115 GMPCPP seeds, respectively, and for 200 nM GFP-EB3 together with 100 nM GFP-CLASP2α, n = 107, 54, 85, 88, 70, 87, 85, and 70 GMPCPP seeds, respectively. Data are from two experiments. Error bars represent SD. Solid lines indicate the sigmoidal equation fit to the data. Tubulin concentration for half-maximal MT outgrowth for tubulin alone = 7.28 ± 0.08, for 200 nM GFP-EB3 = 8.30 ± 0.11, for 100 nM GFP-CLASP2α = 5.35 ± 0.04, for 100 nM GFP-CLASP2α and 200 nM GFP-EB3 = 1.28 ± 0.01. Hill slopes for the fits with tubulin alone = 5.99 ± 0.34, for EB3 = 6.53 ± 0.49, for CLASP2α = 6.46 ± 0.31, and for CLASP2α and EB3 = 3.16 ± 0.07.

For all plots, ****$p < 0.0001$, ns, no significant difference with control, Mann-Whitney U test. See also Figure S1.

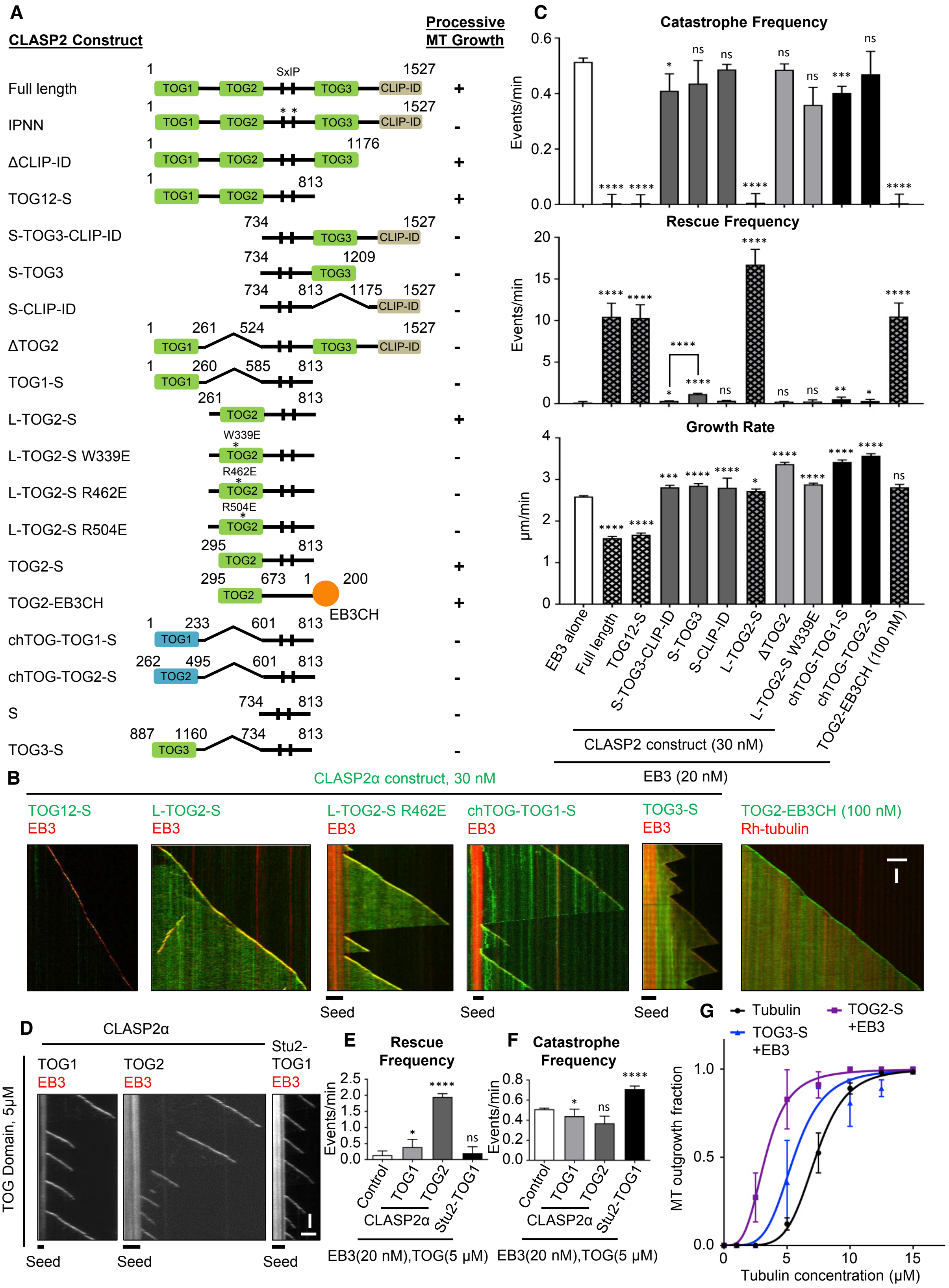

(legend on next page)

300 nM in the presence of EB3ΔTail, but found that this was insufficient to achieve the same MT tip accumulation of CLASP2α, as observed with 30 nM CLASP2α in the presence of full-length EB3 (Figures S1C and S1D). Consistently, we observed no complete catastrophe suppression in these conditions (Figure 1F). Furthermore, we observed a CLASP2α-dependent increase in MT rescues, which did not strictly require CLASP2α accumulation at MT tips, but which was more pronounced when EB3 was present and could interact with CLASPs (Figures 1B–1F). We conclude that CLASPs potently suppress catastrophes when concentrated on MT tips by EB3, and promote rescues in a manner that does not strictly depend on EBs.

To get a better insight into how CLASPs suppress catastrophes, we examined the dynamics of growing MT tips in more detail. By fitting MT fluorescence intensity profiles to the error function to determine the MT tip position with sub-pixel precision, we found that the length variability for MTs grown in the presence of EB3 and CLASP2α was significantly lower than with EB3 alone (Figures 1G, 1H, S1E, and S1F). These data indicate that CLASP2α promotes smooth MT extension by preventing transient episodes of MT tip shortening, suggesting that, in the presence of CLASP2α, MT plus ends are more stable.

It has been shown that factors that destabilize MT tips, such as MT-depolymerizing kinesin-13 MCAK, suppress MT outgrowth from templates, such as stable MT seeds or centrosomes, whereas catastrophe-suppressing factors promote MT outgrowth, an effect that becomes particularly obvious at low tubulin concentrations (Wieczorek et al., 2015). We performed similar assays in which we looked at MT outgrowth from GMPCPP seeds and found that EB3 mildly inhibited MT outgrowth, while CLASP2α alone mildly increased the MT outgrowth frequency (Figures 1I, 1J, and S1G). When combined, CLASP2α and EB3 dramatically increased MT outgrowth from GMPCPP seeds, strongly lowering its kinetic threshold: half-maximal MT outgrowth was observed at a tubulin concentration that was almost 6-fold lower than in the presence of tubulin alone (Figures 1J and S1G). These results support the notion that CLASP2α in complex with EB3 potently promotes formation of stably growing MT plus ends.

A Single MT Tip-Targeted TOG-like Domain of CLASP2 Is Sufficient to Suppress Catastrophes

CLASP1α and 2α consist of three TOG-like domains (termed TOG1, 2, and 3) and a C-terminal domain responsible for interactions with CLIP-170 and other partners, CLIP-interacting domain (CLIP-ID) (Akhmanova et al., 2001; Al-Bassam and Chang, 2011) (Figure 1A). By targeting single CLASP2α domains or their different combinations to MT tips and lattices using a positively charged SxIP-containing peptide of CLASP2 (termed "S" in different abbreviations, Figure 2A), we found that TOG2 was necessary and sufficient to suppress catastrophes (Figures 2A–2C and S2A). Catastrophe suppression was not dependent on the linker region preceding TOG2, but was abrogated when the conserved residues in TOG2, W339, R462, and R504, corresponding to the residues which contribute to MT binding in CLASP1 and to tubulin binding in the XMAP215/ch-TOG family proteins (Leano et al., 2013), were individually mutated to glutamates (Figures 2A–2C, S2A, and S2B). The catastrophe-suppressing properties of CLASP2 TOG2 are unique, because TOG1, TOG3, and CLIP-ID domains of CLASP2 or either of the first two TOG domains of ch-TOG had no effect on MT growth processivity when targeted to MT tips individually by an SxIP peptide (Figures 2A–2C, S2A, and S2B). TOG3 had no effect on catastrophes irrespective of whether it was fused to the N or the C terminus of the SxIP peptide (S-TOG3 or TOG3-S, Figures 2A–2C, S2A, and S2B).

A direct fusion of the CLASP2 TOG2 to the MT tip-binding calponin homology (CH) domain of EB3 (GFP-TOG2-EB3CH) was sufficient to promote processive MT growth (Figures 2A–2C, S2A, and S2C). We note that at low (<100 nM) concentrations, this fusion was less potent than the combination of TOG2-S and EB3 (Figure S2C), likely because it is monomeric and has

Figure 2. The Second TOG-like Domain of CLASP2α Is Necessary and Sufficient for Catastrophe Suppression

(A) A scheme of different CLASP2 constructs used. Processive MT growth is the condition in which no catastrophes were observed within 10 min in the assay with 20 nM mCherry-EB3.

(B) Representative kymographs showing MT plus end growth in the presence of 20 nM mCherry-EB3 and GFP fusions of the indicated fusion proteins. EB3-CH domain fusion was used at 100 nM, all the other proteins at 30 nM. Scale bars, 2 μm (horizontal) and 60 s (vertical).

(C) Parameters of MT plus end dynamics in the presence of 20 nM mCherry-EB3 alone or together with the indicated GFP-fusion proteins. Protein concentrations were as in (B). Number of growth events analyzed: for mCherry-EB3 alone, n = 207, together with GFP-CLASP2α, n = 110, with TOG12-S, n = 110, with S-TOG3-CLIP-ID, n = 117, with S-TOG3, n = 70, with S-CLIP-ID, n = 136, with L-TOG2-S, n = 110, with ΔTOG2, n = 154, with L-TOG2-S W339E, n = 118, with chTOG-TOG1-S, n = 47, with chTOG-TOG2-S, n = 78, and for TOG2-EB3CH alone, n = 110. Error bars represent SEM. For catastrophe frequency plots, *p < 0.05, ***p < 0.005, ****p < 0.0001, for rescue frequency plots, *p < 0.05, **p < 0.005, ****p < 0.0001, and for growth rate plots, *p < 0.05, ***p < 0.005, ****p < 0.0001, and ns, no significant difference with control, Mann-Whitney U test.

(D) Representative kymographs showing MT plus end dynamics in the presence of 20 nM mCherry-EB3 and 5 μM concentration of the indicated TOG domains from CLASP2α or Stu2. Scale bars, 2 μm (horizontal) and 60 s (vertical).

(E and F) MT plus end rescue and catastrophe frequencies in the presence of 20 nM mCherry-EB3 alone (n = 207) or together with 5 μM of CLASP2α TOG1 (n = 61) or TOG2 (n = 100), or with Stu2-TOG1 (n = 146). Error bars represent SEM. For all plots, *p < 0.05, ****p < 0.0001 and ns, no significant difference with control, Mann-Whitney U test.

(G) Plot of the fraction of the total GMPCPP seeds that show MT outgrowth at increasing tubulin concentrations with tubulin alone (black curve) or GFP-EB3 (200 nM) together with either GFP-TOG3-S (100 nM) (blue) or GFP-TOG2-S (100 nM) (purple). For increasing tubulin concentrations in case of tubulin alone, n = 92, 96, 105, 82, 97, 87, 161, and 127 GMPCPP seeds, respectively, for GFP-TOG3-S, n = 61, 52, 53, 56, 71, 59, 61, and 88 GMPCPP seeds, respectively, and for GFP-TOG2-S, n = 70, 64, 50, 50, 55, 66, 63, and 63 GMPCPP seeds, respectively. Data are from two experiments. Error bars represent SD. Solid lines indicate the sigmoidal equation fit to the data. Tubulin concentration for half-maximal MT outgrowth for tubulin alone = 7.28 ± 0.08, for GFP-TOG2-S with GFP-EB3 = 3.29 ± 0.07, and for GFP-TOG3-S with GFP-EB3 = 5.54 ± 0.32. Hill slope for the fits with tubulin alone = 5.99 ± 0.34, for GFP-TOG2-S with GFP-EB3 = 3.56 ± 0.21, and for GFP-TOG3-S with GFP-EB3 = 4.59 ± 1.11.

See also Figure S2.

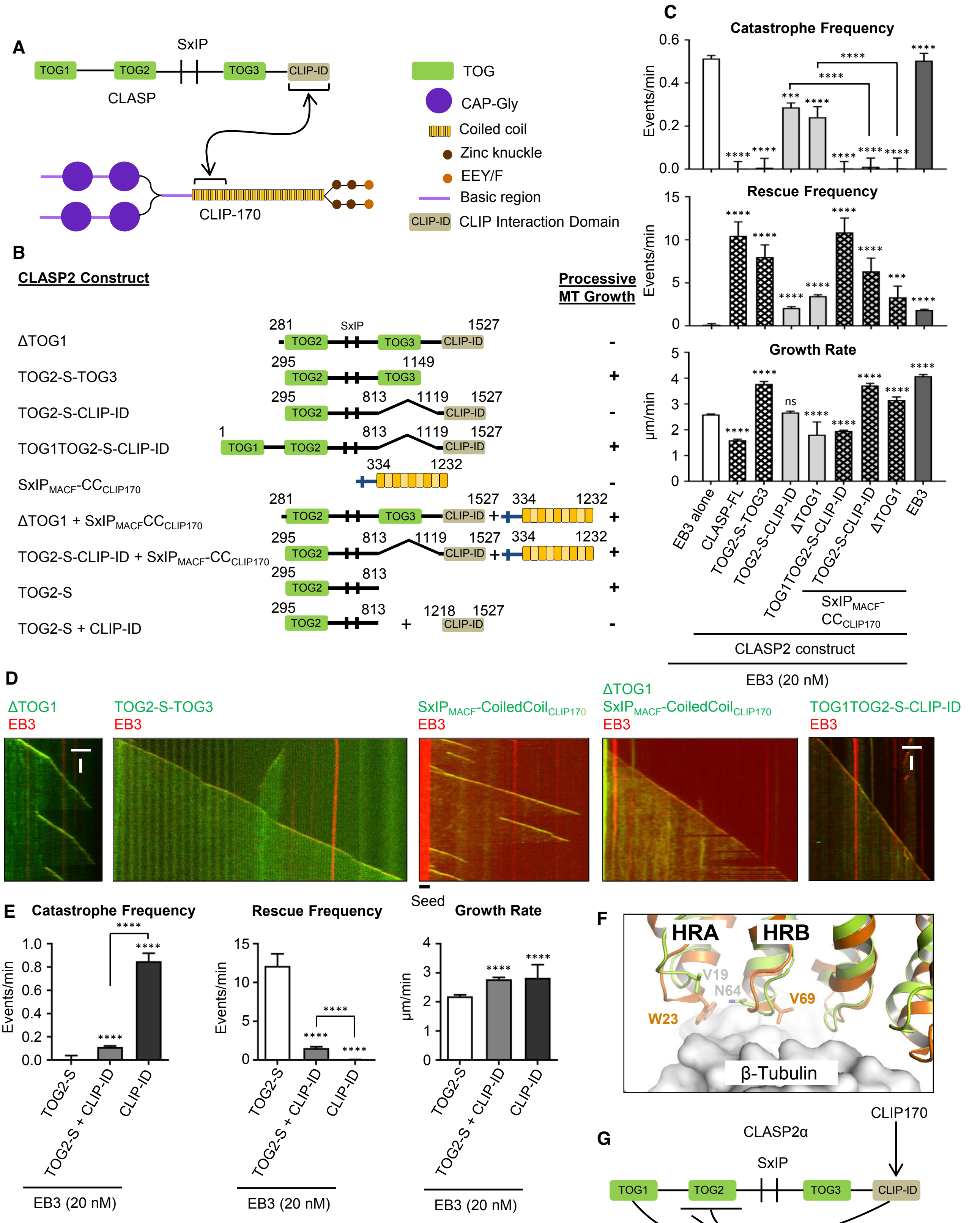

(legend on next page)

a lower MT tip affinity than the full-length EB3, which is a dimer (Sen et al., 2013). Importantly, unlike the other proteins used in this study, which were purified from HEK293T cells, the GFP-TOG2-EB3CH protein was purified from bacteria, excluding possible contamination with MT regulators as a source of catastrophe-inhibiting activity (Figures 2A–2C, S2A, and S2C).

MT tip-targeted TOG2 had little impact on the MT growth rate (Figure 2C), while a TOG1-TOG2-S fusion reduced the MT growth rate similar to the full-length CLASP2α (1.6-fold), suggesting that this effect might be caused by TOG1 or the TOG1-TOG2 combination (Figure 2C). The S-TOG3 fusion led to a ~8-fold increase in the rescue frequency (from 0.14 ± 0.13 min^{-1} with EB3 alone to 1.17 ± 0.08 min^{-1} for S-TOG3 fusion combined with EB3). This effect was suppressed when the C-terminal CLIP-ID domain was also included, leading to a 3.5-fold reduction in the rescue frequency (from 1.17 ± 0.08 min^{-1} with S-TOG3, to 0.34 ± 0.13 min^{-1} for S-TOG3-CLIP-ID fusion, both with EB3) (Figures 2A–2C, and see below). Strikingly, for all constructs containing TOG2, the depolymerization events became extremely short, leading to a dramatic increase in rescue frequency; we note, however, that the number of observed rescues was low due to extremely low catastrophe frequency.

We next investigated the activity of TOG2 without the EB3- and MT lattice-binding SxIP peptide and found that while it had little effect at nanomolar concentrations, at a concentration of 5 μM, it increased the rescue frequency approximately 14-fold (from 0.14 ± 0.13 min^{-1} with EB3 alone to 1.95 ± 0.10 min^{-1} for TOG2 combined with EB3). In contrast, the TOG1 domain of CLASP2 or the tubulin-binding TOG domain of the yeast ch-TOG homolog, Stu2, did not show such an effect (Figures 2D–2F and S2A). The Stu2-TOG1 but not the TOG-like domains of CLASP2 somewhat reduced the MT growth rate, likely by sequestering tubulin dimers (Figures 2D, 2F, and S2D). Furthermore, both TOG2-S and TOG3-S in combination with EB3 individually lowered the kinetic threshold for MT outgrowth from GMPCPP seeds (Figure 2G), but the effect was milder than with the full-length protein (Figure 1J).

Previous analyses of the TOG2 and TOG3 domains of CLASP2 showed that they interact with tubulin ring-like oligomers, but have only a low affinity for MT lattices (Maki et al., 2015). We confirmed that the binding of TOG2 and TOG3 to stabilized MTs was weak, while TOG1 and CLIP-ID did not bind to MTs at all, and none of these domains interacted with free tubulin (Figures S2E–S2I). Together, these results show that TOG2 can potently regulate MT plus end dynamics when targeted to MT plus ends and has an intrinsic rescue activity, although it does not bind to free tubulin. The two latter properties make it distinct from the TOG domains of XMAP215/ch-TOG family of MT polymerases.

Autoregulatory Interactions within CLASP2α

As mentioned above, TOG1 of CLASP2α does not bind to free tubulin or MTs. Strikingly, the deletion of this domain (which converted CLASP2α to the equivalent of the naturally occurring splice isoform CLASP2γ), strongly diminished the ability of CLASP2 to suppress catastrophes, in agreement with a previous publication (Yu et al., 2016) (Figures 3A–3D). This was surprising, as the TOG2 domain sufficient for catastrophe suppression was fully retained in this mutant. Further deletion mapping showed that the presence of CLIP-ID counteracted catastrophe inhibition by TOG2, just as it suppressed MT rescue by CLASP2-TOG3 (Figures 2A–2C, 3A–3D, S2A, S2B, S3A, and S3B). An excess (500 nM) of purified CLIP-ID could reduce the catastrophe-suppressing activity of MT plus end-targeted TOG2, while by itself this protein had little effect on MT dynamics (Figures 3E and S3C).

To explain these results, we hypothesized that CLIP-ID has an auto-inhibitory activity that can be relieved by TOG1. If this were the case, then the binding to partners might release the CLIP-ID-induced inhibition of constructs lacking TOG1. To test this idea, we targeted the CLASP binding coiled-coil domain of CLIP-170 (Figure 3A) to MT tips by fusing it to the EB-binding SxIP motif of MACF2 (Honnappa et al., 2009) (Figure 3B). Addition of this construct potently increased the anti-catastrophe activity of all TOG2-containing CLASP2 constructs that lacked TOG1 but contained CLIP-ID (Figures 3A–3D and S3B). These results suggest that CLIP-ID, when it is not bound to partners such as CLIP-170, has an inhibitory effect on TOG2, and possibly also on TOG3 (Figure 2C), while TOG1 can relieve this inhibition.

Figure 3. The C-Terminal CLIP-Interacting Domain of CLASP2α Shows Auto-inhibitory Activity that Is Relieved by the First TOG-like Domain or by CLIP-170

(A) A scheme of the CLASP-CLIP-170 interaction.

(B) A scheme of the different CLASP and CLIP-170 constructs used. Conditions showing processive MT growth in the presence of 20 nM mCherry-EB3 are indicated based on (C).

(C) Parameters of MT plus end dynamics in the presence of 20 nM mCherry-EB3 and the indicated constructs. Number of growth events: for mCherry-EB3 together with GFP-CLASP2α, n = 110, with TOG2-S-TOG3, n = 62, with TOG2-S-CLIP-ID, n = 101, with ΔTOG1, n = 141, with TOG1TOG2-S-CLIP-ID, n = 116, for mCherry-EB3 and $SxIP_{MACF}$-$CC_{CLIP170}$ alone n = 117, and together with TOG2-S-CLIP-ID, n = 72, with ΔTOG1, n = 50. Error bars represent SEM.

(D) Representative kymographs showing MT plus end dynamics in the presence of 20 nM mCherry-EB3 together with the indicated fusion proteins. Scale bars, 2 μm (horizontal) and 60 s (vertical).

(E) Parameters of MT plus end dynamics in the presence of 20 nM mCherry-EB3 with 30 nM TOG2-S alone (n = 62), or together with 500 nM CLIP-ID (n = 96), or with CLIP-ID alone (n = 115). n = number of growth events. Error bars represent SEM.

(F) Superposition of the structure of hsCLASP2-TOG1 (in green, PDB: 5NR4) and scStu2-TOG1 in complex with tubulin (in orange, PDB: 4FFB) at the β-tubulin binding interface. The scStu2-TOG1 residues located in the two first HEAT repeats (HRA and HRB) and which are involved in tubulin binding, and the equivalent hsCLASP2-TOG1 residues are indicated.

(G) Model for regulation of CLASP activity. CLIP-interacting domain inhibits the catastrophe-suppressing activity of TOG2. In the context of the full-length CLASP2α, this auto-inhibition is relieved by the presence of TOG1, whereas in CLASP2 isoforms such as CLASP2γ, which lack TOG1, the auto-inhibition is relieved by engaging CLIP-ID with the CLIP-170 coiled-coil domain.

For all plots, ***p < 0.005, ****p < 0.0001, and ns, no significant difference with control, Mann-Whitney U test. See also Figure S3.

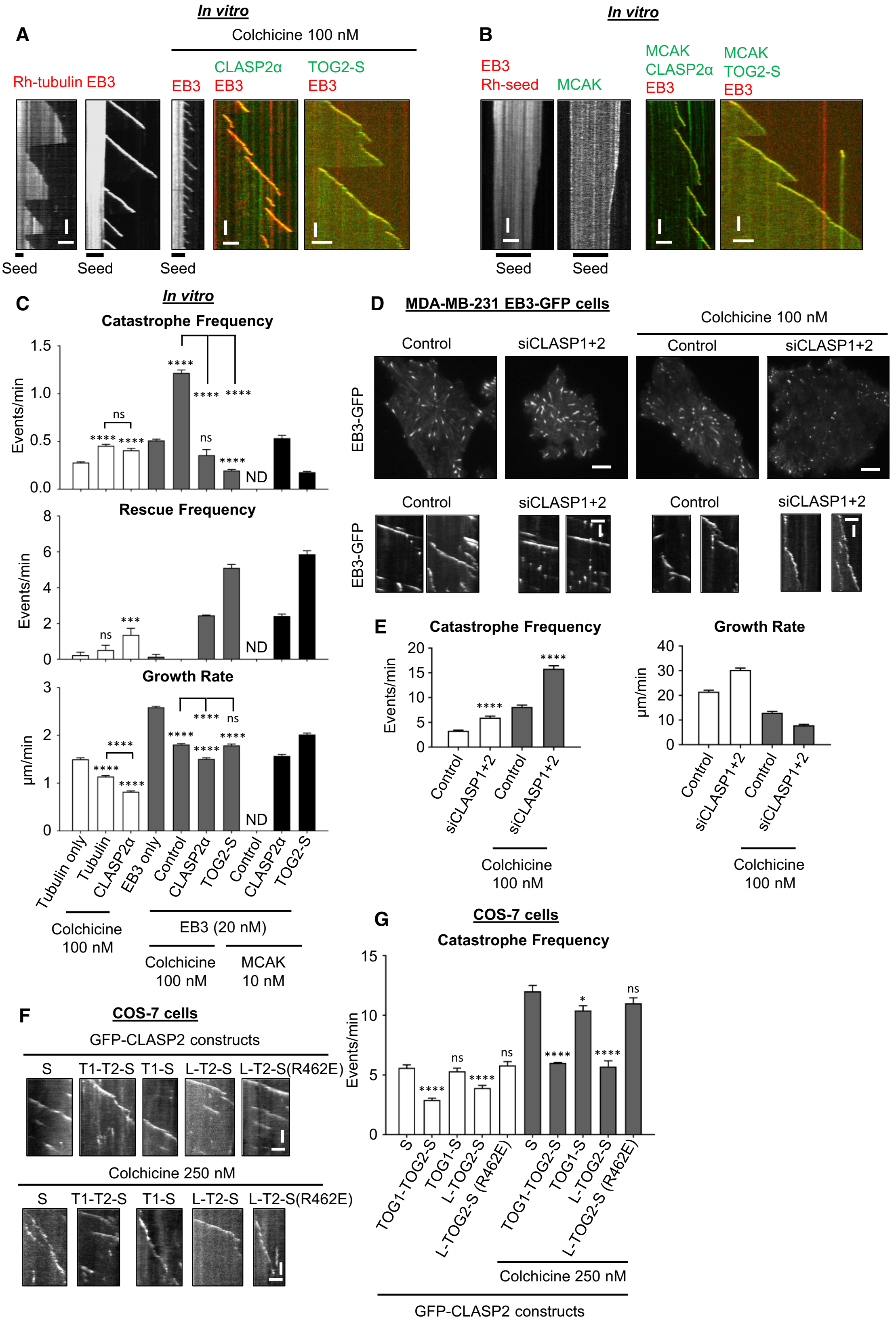

(legend on next page)

To understand why TOG1 does not bind to free tubulin, we solved its structure by X-ray crystallography. The structure of TOG1 showed a conserved TOG-domain fold, but also demonstrated that the conserved residues required for tubulin interaction are lacking (Figures 3F and S3E). This explains why TOG1 does not bind to either free tubulin or MTs (Figures S2E and S2F). We also checked whether TOG1 could bind to alternative tubulin structures, such as tubulin rings induced by dolastatin or vinblastine, but found this not to be the case (Figure S3D). Thus, in contrast to a previous publication suggesting that the ability of TOG1 to bind free tubulin is required for CLASP activity (Yu et al., 2016), we establish that TOG1 has an autoregulatory function (Figure 3G). We note that we were unable to detect direct interactions between isolated TOG2 and TOG1 or CLIP-ID by biophysical methods (Figures S3F–S3I), which is not surprising because autoregulatory interactions within proteins are often weak and difficult to detect using isolated protein fragments. Based on these data, we propose that the TOG1-containing CLASP1/2α isoforms are constitutively active, whereas the CLASP2β/γ isoforms, which lack TOG1 (Akhmanova et al., 2001), require partners interacting with CLIP-ID for their optimal activity.

CLASP2α Suppresses Catastrophes Induced by MT-Depolymerizing Agents

The data described above revealed that CLASPs suppress spontaneous catastrophes. However, in cells catastrophes are often induced by MT-destabilizing factors (Gardner et al., 2013). To test if CLASPs can counteract the action of such factors, we first tested the effect of MT-depolymerizing drugs, such as colchicine and vinblastine, which perturb MT plus end structure and induce catastrophes in the presence of EBs (Mohan et al., 2013). We found that CLASP2α indeed promoted longer MT polymerization events at drug concentrations that strongly inhibited MT growth (Figures 4A, 4C, and S4A). Similarly, CLASP2α counteracted the activity of MCAK, an MT depolymerase that induces protofilament curling, even when the latter was added at a concentration that, in the absence of CLASP2α, was sufficient to completely block MT outgrowth and cause depolymerization of MT seeds (Figures 4B, 4C, and S4B). The minimal catastrophe-suppressing module TOG2-S could also counteract the catastrophe-inducing action of colchicine and promoted MT growth in the presence of MCAK, and was even more effective than full-length CLASP2α (Figures 4A–4C).

We next tested whether CLASPs can protect MTs from drug-induced catastrophes in cells. Simultaneous depletion of CLASP1 and CLASP2 in cells stably expressing EB3-GFP led to a mild increase in the MT catastrophe frequency in internal cell regions and resulted in more frequent catastrophes in cells treated with colchicine (Figures 4D and 4E). Importantly, the expression of MT tip-targeted TOG1-TOG2-S and TOG2-S fusions, but not of TOG1-S or the mutated version of TOG2-S, caused a mild catastrophe inhibition in control cells and strongly suppressed catastrophes in colchicine-treated cells (Figures 4F, 4G, S4C, and S4D). These data show that the TOG2 domain potently counteracts catastrophes induced by agents that perturb the MT end structure in different ways both *in vitro* and in cells.

CLASP2α Suppresses Force-Induced Catastrophes

Next, we investigated whether CLASP2α is capable of suppressing catastrophes induced by compressive forces. It was previously shown that when a growing MT polymerizes against a solid barrier, the ensuing compressive force can advance the onset of a catastrophe (Janson et al., 2003). We used micro-fabricated barriers composed of SiO_2 etched on a glass coverslip (Kalisch et al., 2011). This fabrication process resulted in 1.7-μm-high barriers enclosing 15-μm-wide channels (Figure 5A). MTs were allowed to grow from GMPCPP-stabilized seeds inside the channels and polymerize toward the barriers from varying angles and distances. The interaction of the MT plus end with the barrier gave rise to three different possible outcomes: sliding (bending and growing along the barrier), stalling, and buckling (Figure 5B and Videos S1, S2, and S3). Stalling indicates that an MT is unable to overcome the compressive force build-up during barrier contact, and therefore cannot continue polymerizing, but instead remains in a static contact with the barrier until the onset of a catastrophe. Buckling occurs when an MT contacting the barrier keeps growing while its end remains at the same position at the barrier (indicative of a moderate compressive force).

In the absence of EB3 and CLASP2α, sliding behavior predominated for all contact angles due to the smooth surface of the

Figure 4. CLASP2α Suppresses Catastrophes Induced by MT-Destabilizing Agents *In Vitro* and in Cells

(A) Kymographs showing MT plus end dynamics in the presence of rhodamine-tubulin alone or with 20 nM mCherry-EB3 or in the presence of 100 nM colchicine with 20 nM mCherry-EB3 alone or together with 30 nM GFP-CLASP2α. Scale bars, 2 μm (horizontal) and 60 s (vertical).

(B) Kymographs showing MT plus end depolymerization in the presence of 20 nM mCherry-EB3 and 10 nM GFP-MCAK, or plus end growth dynamics when 30 nM GFP-CLASP2α or GFP-TOG2-S are added. Scale bars, 2 μm (horizontal) and 60 s (vertical).

(C) Parameters of MT plus end dynamics in the presence of the indicated of proteins, with or without 100 nM colchicine. Number of growth events analyzed: for rhodamine-tubulin alone, n = 135, with colchicine, n = 110, with colchicine and GFP-CLASP2α, n = 68, for mCherry-EB3 alone, n = 207, for mCherry-EB3 with colchicine, n = 228, for mCherry-EB3 with colchicine and GFP-CLASP2α, n = 136, and for mCherry-EB3 with colchicine and GFP-TOG2-S, n = 241. For mCherry-EB3 with GFP-MCAK and GFP-CLASP2, n = 144 and for mCherry-EB3 together with GFP-MCAK and GFP-TOG2-S, n = 227. Error bars represent SEM.

(D) Still images of MDA-MB-231 cells stably expressing EB3-GFP and kymographs showing MT plus end growth in control or CLASP1- and CLASP2-depleted cells alone or in the presence of 100 nM colchicine. Scale bars, 5 μm (cell images), 2 μm (horizontal), and 60 s (vertical) (for kymographs).

(E) MT plus end catastrophe frequency and growth rates in MDA-MB-231 cells stably expressing EB3-GFP after transfection either with control or CLASP1 and CLASP2 siRNAs, untreated or treated with 100 nM colchicine. Number of growth events from left to right, n = 56, 53, 106, and 123. Error bars represent SEM.

(F) Kymographs showing MT plus end dynamics in COS-7 cells expressing the indicated GFP-fusions; cells were untreated or treated with 250 nM colchicine. Scale bars, 2 μm (horizontal) and 15 s (vertical).

(G) MT plus end catastrophe frequency in COS-7 cells shown in (F). Numbers of growth events from left to right n = 61, 61, 65, 64, and 57 (without colchicine) and with 250 nM colchicine, n = 61, 65, 92, 47, and 70 (with 250 nM colchicine). Error bars represent SEM.

For all plots, *p < 0.05, ***p < 0.005, ****p < 0.0001, and ns, no significant difference with control, Mann-Whitney U test. See also Figure S4.

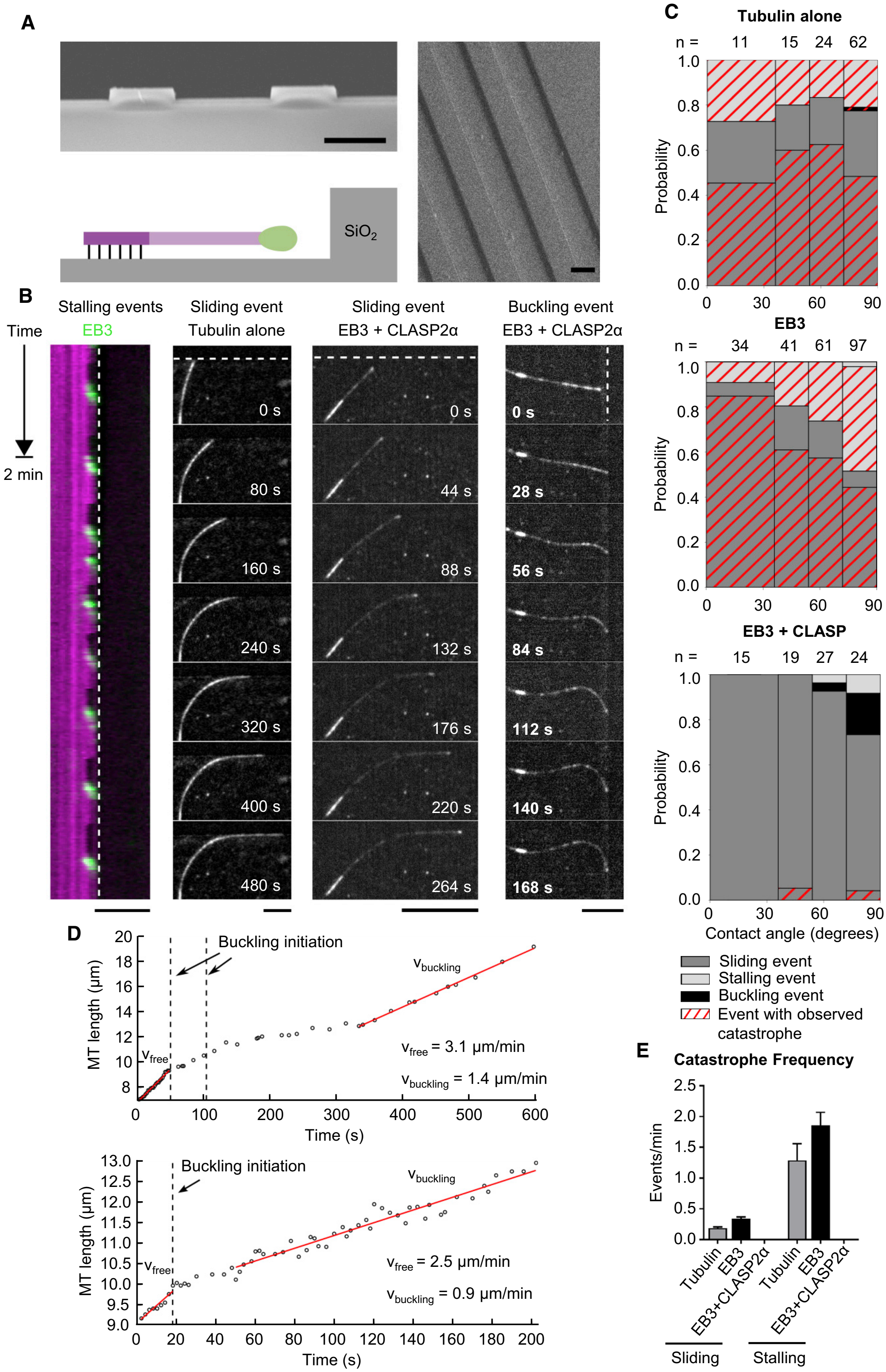

(legend on next page)

barriers, while the addition of EB3 led to an increase in stalling events, particularly when the seeds were perpendicular to the barriers (Figures 5B and 5C and Video S1). The catastrophe frequency during contact in both cases was higher for stalling than for sliding, and was particularly high for MTs stalled at barriers in the presence of EB3, clearly showing that the MT plus end is less stable at high compressive forces in these conditions (Figure 5E). Strikingly, the addition of EB3 and CLASP2α resulted in persistent MT growth almost devoid of observable catastrophes for all event types (Figure 5D and Videos S2 and S3). A few buckling events were observed in the presence of EB3 and CLASP2α at almost perpendicular contact angles (Figure 5D and Video S3). During buckling, the MT growth speed decreased compared with the growth speed prior to barrier contact, but remained constant after an initial pausing phase (Figure 5D). These data show that CLASP2α can prevent destabilization of a growing MT tip during barrier contact even at high compressive forces during buckling.

TOG2 Shows Preference for a Region Located Behind the Outmost MT End

To get better insight into how TOG2 prevents catastrophes, we next examined the behavior of this domain tethered to MTs by the positively charged SxIP peptide (TOG2-S) and found that, at concentrations between 200 and 400 nM, it showed enrichment at the GMPCPP seeds and growing MT ends in the absence of EB3 (Figure 6A), while the SxIP peptide alone showed no autonomous MT tip enrichment (Honnappa et al., 2009, data not shown). A similar, albeit weaker MT tip enrichment was found in the presence of EB3ΔTail, which does not bind to TOG2-S (Figure S5A). In contrast, no TOG2-S accumulation was present at depolymerizing ends, also when MT disassembly was induced in the absence of free tubulin (Figures 6B and S5B). At 200–400 nM, TOG2-S reduced catastrophes, stimulated rescues, and induced occasional pausing events, while the MT growth rate was mildly reduced (Figures 6C, S5C, and S5D). TOG2-S-induced pauses or periods of very slow growth with duration of up to ~60 s were particularly obvious in the presence of EB3ΔTail, as they were never observed with EB3 or EB3ΔTail alone. Some weak EB3ΔTail accumulation at MT tips was present during such events (Figure S5D), suggesting that they maintain a short stabilizing cap (Maurer et al., 2012).

We next used high-resolution simultaneous dual-color TIRFM imaging of GFP-TOG2-S together with rhodamine-tubulin or mCherry-EB3ΔTail, in combination with automated data analysis with sub-pixel precision and convolved model fitting, to extract molecular density distributions of TOG2-S relative to the MT end and the EB3ΔTail comet, following the procedures described previously (Maurer et al., 2014). For the fitting, we modeled MT intensity as a step function, TOG2-S intensity as a combination of a step function (lattice intensity) and a delta function (point peak accumulation), and EB3ΔTail comet as an exponential decay function (Figures 6D–6F, S5E, and S5F). Peak-to-lattice ratios of fluorescence intensity of TOG2-S were higher for higher growth rates (Figure 6G): a clear peak of TOG2-S was visible for MTs growing faster than 1 μm/min, but not at lower rates (Figures 6H and 6I). Analysis of the averaged profiles indicated that the TOG2-S peak, when detectable, was positioned ~90 nm behind the MT end (Figures 6J and S5I). Similar analysis of averaged profiles with EB3ΔTail showed that the TOG2-S peak-to-lattice ratio was lower (Figures S5G and S5H), and that TOG2-S peak was centered at ~60 nm behind the EB3ΔTail peak (Figures 6J, 6K, and S5J).

Previous work showed that the stabilizing (GTP or GDP-Pi) MT cap detected with EB1 as a marker starts at ~20–90 nm behind the MT end and decays for 200–500 nm, depending on the EB1 concentration and MT growth rate (Maurer et al., 2014). This localization broadly fits with the position of the TOG2-S peak detected here with tubulin alone. However, simultaneous imaging of EB3ΔTail and TOG2-S showed that the peaks of the two proteins do not coincide, but that the maximal accumulation of TOG2-S was positioned at the rear of the EB3 comet (Figures 6J and 6K), and the amplitude of this accumulation was reduced compared with the assays with tubulin alone (Figures S5G and S5H). These findings can be explained by a combination of factors, such as the effect of EB3ΔTail on the MT lattice structure or GTP hydrolysis (Maurer et al., 2014), or direct competition between TOG2-S and EB3ΔTail. Taken together, these data indicate that, in the conditions used in our study, TOG2-S shows a preference for MT end sites enriched during rapid MT growth and located ~10–12 tubulin layers behind the outmost end. This region likely represents a part of the MT-stabilizing cap and not the strongly curved protofilament ends that could be present at outmost MT extremities. In line with this view, TOG2-S showed no accumulation at depolymerizing MT ends, and both CLASP2α and TOG2-S did not slow down, but rather mildly increased MT depolymerization rate (Figures S5K and S5L), possibly because MT lattice polymerized in the presence of CLASPs has different properties (Grimaldi et al., 2014). Consistently, a very recent paper showed that CLASP2γ mildly increased MT shrinkage rate (Lawrence et al., 2018). Collectively, our data indicate that TOG2 acts to suppress MT

Figure 5. CLASP2α Inhibits Force-Induced Catastrophes in the Presence of EB3

(A) Scanning electron microscope images with cross-sectional and top-down view of the SiO_2 barriers. The cartoon illustrates the MT-barrier interaction of a seed-nucleated MT in the presence of MT tip-binding proteins. Scale bars, 10 μm.

(B) Representative kymograph and three-frame averaged montages of the three types of events during MT-barrier contact: stalling, sliding, and buckling. The location of the barrier is denoted by dashed white lines. All experiments were performed at 30°C, with the following concentrations when present: tubulin (15 μM), EB3 (20 nM), and CLASP (30 nM). Scale bars, 10 μm. See also Videos S1, S2, and S3.

(C) Probability of the event type during MT-barrier contact as a function of the contact angle, with 90° being perpendicular to the barrier. The red hatched events ended with a catastrophe. Number of growth events analyzed are indicated above each bin.

(D) MT growth during two buckling events. Vertical dotted lines indicate the start of a buckling event. The first graph contains two buckling initiation events, as the MT tip slipped during the first event. MT growth velocities are significantly lower during buckling compared with free growth.

(E) MT plus end catastrophe frequency during barrier contact for MTs sliding or stalling in the presence of tubulin alone or together with 20 nM mCherry-EB3 alone or with 20 nM mCherry-EB3 and 30 nM GFP-CLASP2α. For sliding events, n = 88, 156, and 77, and for stalling events, n = 23, 77, and 3 for MTs grown in the presence of tubulin alone, together with mCherry-EB3, and with both mCherry-EB3 and GFP-CLASP2α. Error bars represent SEM.

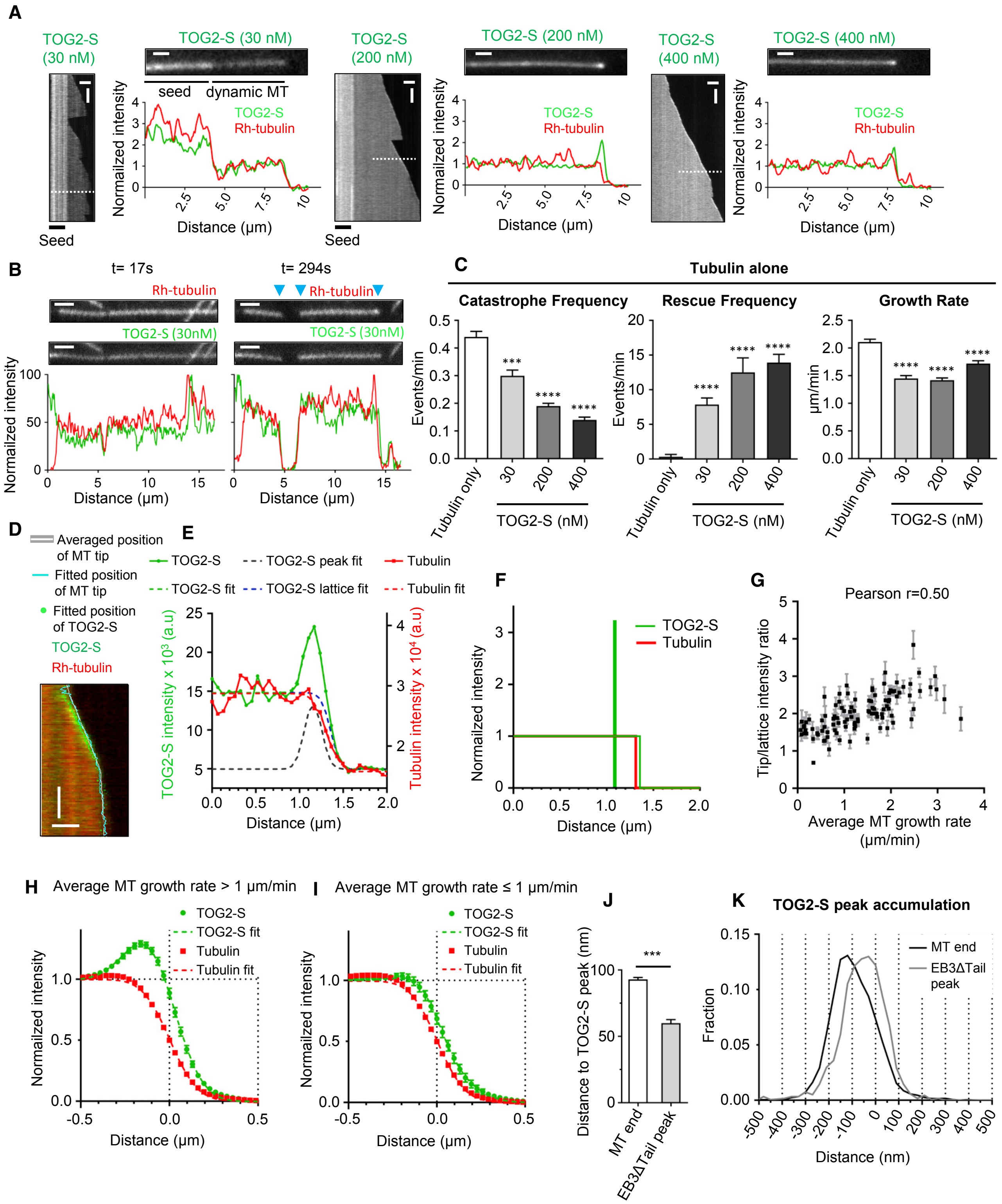

Figure 6. TOG2 Domain Shows Preference for a Region behind the Outmost MT End

(A) Kymographs, stills, and fluorescence intensity profiles for GFP-TOG2-S at the indicated concentrations (30, 200, and 400 nM) in the presence of rhodamine-tubulin. Scale bars, 1 μm (for stills). Scale bars, 3 μm (horizontal) and 60 s (vertical) (for kymos).

(B) Stills and fluorescence intensity profiles for GFP-TOG2-S (30 nM) and rhodamine-tubulin for MTs assembled in the presence of tubulin alone after tubulin washout, with the time after tubulin washout indicated. Blue arrowheads indicate the depolymerizing MT ends. Scale bars, 2 μm.

(legend continued on next page)

catastrophes by binding behind the outmost MT end before rapid MT depolymerization is initiated.

A Few CLASP2α Monomers Stabilize Incomplete MT Structures

Next, we used single-molecule analysis to investigate the number and the residence time of CLASP2α molecules suppressing catastrophes. As published previously (Drabek et al., 2006), we found that mammalian CLASP2α is monomeric (Figure S6A). The interactions of CLASP2α and TOG2-S with MT tips and lattices in the presence of EB3 in our assays were very transient (Figures S6B–S6D), with an average residence time at MT tips of ~0.2–0.3 s, similar to that previously shown for other MT tip-tracking proteins (Bieling et al., 2007; Montenegro Gouveia et al., 2010). We note that this residence time was shorter than that recently described for CLASP2γ in the presence of EB1 (Lawrence et al., 2018), possibly due to differences in the proteins used or assay conditions.

By using single GFP-CLASP2α molecules immobilized in a separate chamber on the same coverslip used for the MT dynamics assay, we estimated the number of CLASP2α molecules necessary for catastrophe suppression and rescue induction. Due to the exponential decay of the TIRF field, the brightness of a molecule attached to an MT compared with a molecule attached to the glass surface would be lower, but the underestimate is in the range of 10% (van Riel et al., 2017). We found that one or two transiently binding molecules were sufficient to induce rescues at 3 nM CLASP2α (Figures S6E and S6F). Rescues often occurred after a short event of CLASP2α tracking the depolymerizing MT end (Figures S6E, white arrow). In contrast to the previous work on yeast and *Drosophila* CLASPs (Al-Bassam et al., 2010; Moriwaki and Goshima, 2016), but consistent with the work on mammalian CLASP2γ (Lawrence et al., 2018), rescues thus did not require the accumulation of immobile CLASP2α clusters on the MT lattice. At the MT tip, 4–7 CLASP2α molecules were typically present in conditions when catastrophes were fully suppressed (Figure S6F). Together, these data indicate that a small number of CLASP2α molecules (less than 10) are sufficient to suppress catastrophes, and even fewer CLASP2α molecules can promote rescues.

Interestingly, the examination of kymographs of MT growth in the presence of CLASPs or the MT tip-targeted TOG2 domain often revealed the presence of two EB3 comets on the same MT–events whereby an MT tip polymerization slowed down and was subsequently restored by a “catching up” (a rear) EB3 comet that appeared behind the growing tip and was moving more rapidly than the “leading” comet (Figures 7A–7E). We termed such events as “tip repair” events. It has been previously shown that such events could occasionally be observed with MTs grown in the presence of EB3, and that their frequency could be strongly increased by the protofilament-blocking agent eribulin, indicating that they occur when some protofilaments in an MT temporarily lag behind and then resume growth to “catch up” with the growing end (Doodhi et al., 2016). In the presence of full-length CLASP2α, frequent and long tip repair events were observed, with the duration often exceeding 60 s and the length of up to 3–4 μm (Figures 7A, 7F, S7A, and S7B). An increase in the frequency of tip repair events was also observed at a high concentration (400 nM) of TOG2-S together with EB3ΔTail (Figures S7C and S7D). The idea that some protofilaments are missing from the MT end corresponding to the leading comet was supported by the reduced intensity of CLASP2α or TOG2-S bound to the MT lattice in these regions (Figures 7B and S7E), and by the observation that such ends were often bent or curled, suggesting the loss of the mechanical integrity of the tube-like MT structure (Figure 7G and Video S4). Tip repair events that exhibited curling were also observed in cells (Figure 7H and Video S5), indicating that they are not an artifact of *in vitro* reconstitution.

To test if the presence of two EB3 comets on the same MT is indeed caused by stalling of a subset of the protofilaments, we collected data in the presence of TagBFP-CLASP2α, mCherry-EB3, and HiLyte488-labeled tubulin and analyzed MT intensity profiles in the tubulin channel during comet splitting (Figure 7I). As expected, the MT intensity in the region between the leading and lagging comets was lower than the region behind the lagging comet (Figure 7I, bottom panel). We considered two simple models of MT tip “erosion”: a subset of *N* protofilaments could be shortened by a uniform value *d* from the growing tip, creating a sharp drop in intensity (model A, Figure 7J), or the erosion could be gradual, and we assumed that the lengths of the missing parts of *N* protofilaments were distributed exponentially with a characteristic value *d* (model B, Figure 7J). Using these models, we performed Monte Carlo simulations of the MT

(C) Parameters of MT plus end dynamics in the presence of either rhodamine-tubulin alone (n = 122) or in combination with GFP-TOG2-S at 30 nM (n = 91), 200 nM (n = 109), and 400 nM (n = 80). Error bars denote SEM. For all plots, ***p < 0.005, ****p < 0.0001, and ns, no significant difference with control, Mann-Whitney U test.
(D) Example kymograph of GFP-TOG2-S (400 nM) (green channel) and rhodamine-tubulin (red channel) with overlayed profiles fitting results. Cyan line marks fitted position and white line marks averaged position of MT tip, derived from piecewise linear approximation. Green dots mark fitted position of TOG2-S accumulation. The opacity of dots is proportional to the amplitude of the accumulation. Scale bars, 1 μm and 10 s.
(E) Example of an individual fitting of GFP-TOG2-S (400 nM) and rhodamine-tubulin fluorescence intensity profiles. Blue dashed line corresponds to the lattice and black dashed line to the peak accumulation components of overall TOG2-S fit function (shown with green dashed line).
(F) Density distribution of TOG2-S (400 nM) (peak and lattice component shown separately, green lines) and MT lattice (red line) extracted from fitting shown in (E).
(G) Plot of the ratio of peak-to-lattice fluorescence intensity of GFP-TOG2S (400 nM) derived from fitting versus average speed of growth. Ratios are averaged for segments of constant average speed growth (128 segments, 5997 time points, 13 kymographs). Error bars represent SEM.
(H and I) Normalized, aligned, resampled, and averaged fluorescent intensity profiles of GFP-TOG2-S (400 nM) and rhodamine-tubulin split based on average MT growth rate threshold value of 1 μm/min. Profiles were first averaged per kymograph. Error bars represent SEM of second averaging among multiple kymographs (13 kymographs, 3,536 left + 2,461 right = 5,997 total time points).
(J and K) Mean values with SEM (J) and histograms (K) of distances between TOG2-S intensity peak accumulation and the fitted position of MT tip (white bar, mean = 92.9 nm, n = 4586 fits) or the fitted peak of EB3ΔTail (gray bar, mean = 59.9 nm, n = 1778 fits). Only the fits where TOG2-S tip to lattice intensity ratio was above 1 were included. ***p < 0.0001, two-tailed Mann-Whitney test. In the histograms, 0 corresponds to the fitted position of the MT end or EB3ΔTail peak.
See also Figure S5.

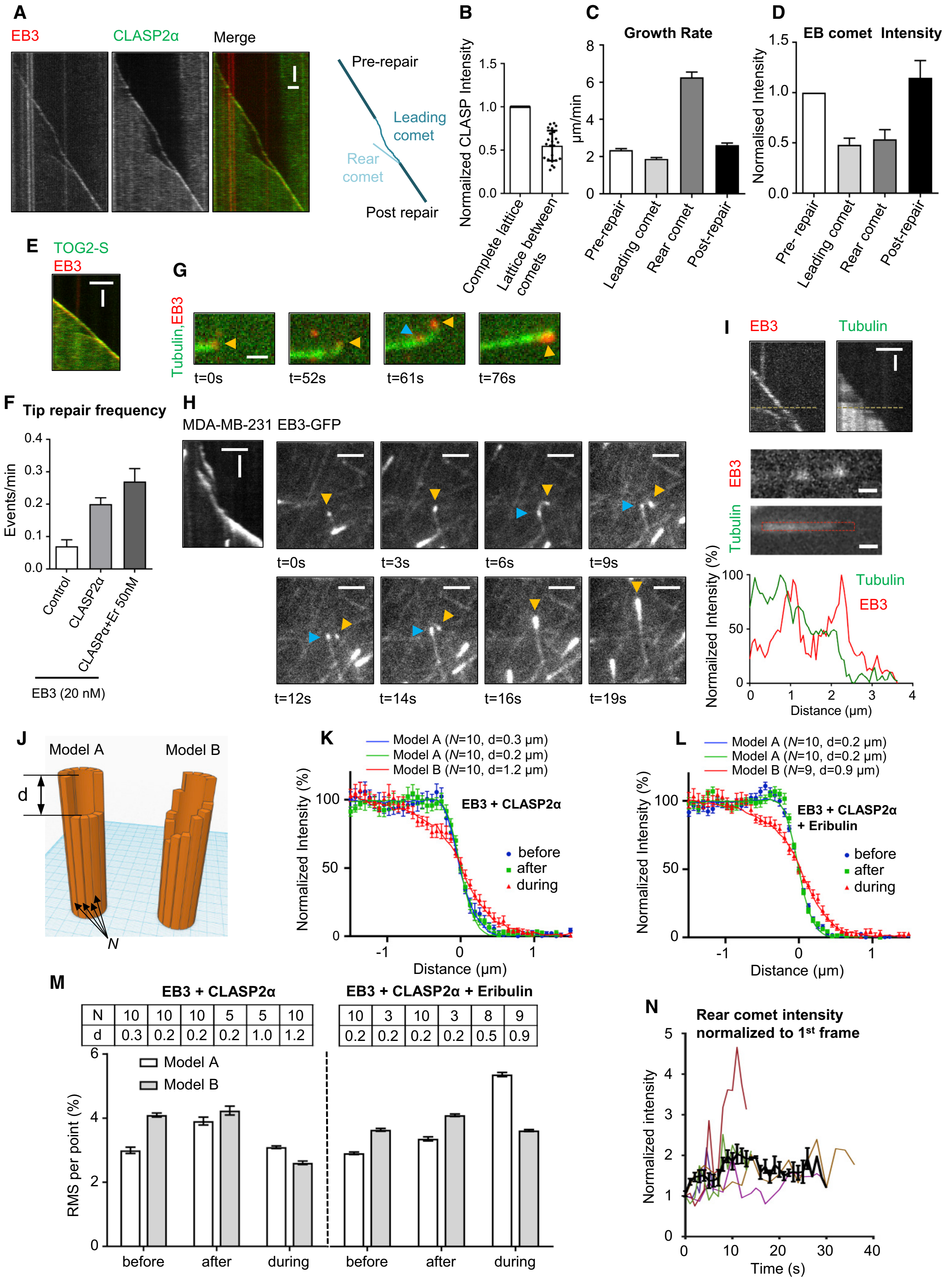

(legend on next page)

intensity, assuming that an MT has 13 protofilaments and that tubulin dimers are labeled with a probability equal to the fraction of labeled tubulin in the reaction (Figures S7F–S7J, see STAR Methods for details).

We analyzed two types of experimental data, the ‘‘control’’ situation with CLASP2α and EB3, or the situation where the reaction was supplemented with 50 nM eribulin, which increases the frequency of detectable tip repair events (Figure 7F). For each condition, experimental intensity profiles of tubulin were recorded at three time points: before, during, and after the tip repair event (Figures 7K and 7L). The time point during tip repair was selected at the moment when the distance between comets was approximately equal to 1 μm. For each model, the parameters *N* and *d* were varied in search of values minimizing the residual between the experimental and theoretical profiles (Figure 7M). Results for both "before" and "after" conditions were very similar and favored model A (sharp drop) with $N = 10$ missing protofilaments (or three protruding protofilaments) with a characteristic length *d* of 0.2–0.3 μm (Figures 7K–7M). For the catching up phases, the experimental profiles were better approximated by model B (gradual) with nine to ten missing protofilaments and a much longer erosion length *d* of 0.9–1.2 μm (Figures 7K–7M). If the assumption of gradual tip erosion from model B is correct, this means that the rear EB3 comet intensity should gradually increase during the tip repair event, as more protofilaments are joining it, and we found that this is indeed the case (Figure 7N). This increase was not due to the increasing velocity of the rear comet, which could affect EB3 intensity (Figure S7K), and these data thus support the results of modeling. Taken together, our results show that a few monomeric CLASP2α molecules transiently associated with MT tips prevent the onset of a catastrophe at MT ends missing a number of protofilaments and promote restoration of the MT tip structure (Figure S7L).

DISCUSSION

CLASPs are among the most conserved regulators of MT dynamics, which are present in animals, plants, and fungi, where they can suppress catastrophes and induce rescues (Bratman and Chang, 2007; Ruan and Wasteneys, 2014). CLASPs contain several TOG-like domains, and we found that TOG2, targeted to the MT tip, is necessary and sufficient for catastrophe suppression. Importantly, TOG2 by itself does not interact with free tubulin, and the same is true for the other folded CLASP domains. TOG2 also has only a very weak affinity for MTs; however, it normally acts as a part of CLASP, which interacts with MTs through unstructured positively charged regions and the TOG3 domain, and is targeted to the MT end-stabilizing cap by the nucleotide-sensitive CH domain of EBs (Honnappa et al., 2009; Maurer et al., 2012). Interestingly, when tethered to MTs by a positively charged peptide, TOG2 shows some autonomous preference for the region overlapping with the stabilizing MT cap. These data suggest that the GTP-hydrolysis-dependent MT-stabilizing region, located behind the outmost MT tip is the actual site of the anti-catastrophe activity of TOG2. This view fits with our observations that CLASPs potently inhibit catastrophes irrespective of how they are initiated at the MT tip—spontaneously, mechanically, or by drugs that might create defects or loss of individual protofilaments, or by an MT depolymerase that triggers protofilament peeling. This idea is also in line with the fact that TOG2 or CLASP2α do not accelerate MT polymerization: TOG2 shows no preference for the outmost MT end and does not bind to free tubulin, and therefore does not share these two distinguishing features of the MT polymerases of the XMAP215/ch-TOG family (Brouhard et al., 2008; Maurer et al., 2014). Our data thus exclude the model that mammalian CLASPs act like MT polymerases that recruit tubulin dimers to MTs (Yu et al., 2016), although this model may still hold true for the yeast

Figure 7. CLASP2α Stabilizes Incomplete MT Tip Structures

(A) Kymographs showing an MT tip repair event with 20 nM mCherrry-EB3 and 30 nM GFP-CLASP2α; a schematic of the same event is shown on the right. Scale bars, 2 μm (horizontal) and 60 s (vertical).

(B) Averaged fluorescence intensity of GFP-CLASP2α in the MT lattice region between the leading and lagging comet, normalized to the intensity of the complete MT lattice (n = 23). Mean ± SD.

(C and D) Growth rates (C) (n = 65 events) and the EB-comet intensities (D) (n = 17 events) before, during, and after comet splitting. EB-comet intensities are normalized to the comet intensity before splitting.

(E) Kymograph showing a tip repair event in the presence of 20 nM mCherry-EB3 and 30 nM GFP-TOG2-S. Scale bars, 2 μm (horizontal) and 45 s (vertical).

(F) Frequency of tip repair for MTs grown in the presence of 20 nM mCherry-EB3 alone (n = 49) or together with 30 nM TagBFP-CLASP2α (n = 103), or in the presence of 20 nM mCherry-EB3, 30 nM TagBFP-CLASP2α, and 50 nM Eribulin-A488 (n = 56). The frequency was calculated by dividing the number of observed tip repair events by the total growth time, n is the number of MTs analyzed in each condition. Error bars represent SEM.

(G) Still images of an MT grown in the presence of Alexa 488-tubulin, 20 nM mCherry-EB3, and 30 nM TagBFP-CLASP2α, showing curling in the region between the leading and the lagging comet. Arrowheads point to the EB comets, yellow points to the leading comets, and blue points to the rear comets. Scale bar, 1 μm.

(H) Kymograph and corresponding still images showing an MT tip repair event in MDA-MB-231 cells stably expressing EB3-GFP. The yellow arrowhead points to the leading comet and the blue ones to the rear comets. Scale bars, 2 μm (horizontal) and 5 s (vertical) (kymograph) and 2 μm (cell image).

(I) Kymograph showing an MT tip repair event in the presence of HiLyte-488 tubulin, 20 nM mCherry-EB3, and 30 nM TagBFP-CLASP2α, still images and line-scans along the red (EB3) and green (tubulin) channel during tip repair. Scale bars, 2 μm (horizontal) and 30 s (vertical) (kymograph) and 0.5 μm (still images).

(J) Illustration of two different MT end tapering models representing sharp (model A, left) and gradual (model B, right) loss of protofilaments.

(K and L) Averaged tip intensity profiles of tubulin channel (green) for MTs grown in the presence of 20 nM mCherry-EB3 and 30 nM TagBFP-CLASP2α (K), n = 16, 17, and 17 for before, after, and during tip repair, respectively, and for MTs grown in the presence of 20 nM mCherry-EB3, 30 nM TagBFP-CLASP2α, and 50 nM Eribulin, n = 40, 44, and 27 for before, after, and during tip repair, respectively. Error bars represent SEM. Lines correspond to the best fits of simulations with the optimal model type and parameter values indicated at the top of each plot.

(M) The distribution of minimal residuals between simulated and experimental profiles depending on the model. Top table shows optimal parameter values for each case (d is in μm). For each case n = 3. Error bars represent SD.

(N) Changes of the mCherry-EB3 comet intensity over time for the lagging comet before the tip repair. Individual traces represent a single tip repair event. The black line is the average of several time traces (n = 22). Intensity values were normalized to the value at the first time point.

See also Figures S6 and S7 and Videos S4 and S5.

CLASP homologs (Al-Bassam et al., 2010). The difference between CLASPs and the MT polymerases of the XMAP215/ch-TOG family is further emphasized by the observation that the latter do not suppress catastrophes or induce rescues *in vitro* (Zanic et al., 2013), and our experiments showed that, in contrast to the CLASP2 TOG2, targeting of individual tubulin-binding TOG domains of ch-TOG to growing MT tips had little effect on catastrophes and rescues.

The nature of the exact binding site of TOG2 remains an important question. TOG2 has a unique convex architecture with an additional N-terminal helix that stabilizes the domain's paddle-like HEAT repeat structure (Leano et al., 2013; Maki et al., 2015), and it has been shown that it displays a higher affinity for drug-induced tubulin ring-like oligomers (Maki et al., 2015). We showed that the TOG2 residues W339, R462, and R504, corresponding to the amino acids that contribute to tubulin binding in the TOG domains of XMAP215/ch-TOG family proteins, are required for TOG2 function in our assays, in agreement with the previous analysis of MT binding by CLASP1 (Leano et al., 2013). However, this does not mean that CLASP TOG2 binds to highly bent tubulin dimers. TOG2-S does not concentrate on depolymerizing MT ends or at the outmost ends of growing MTs, where the most strongly curved conformation of tubulin dimers is expected to be found. Since the TOG2 structure is unique, its binding site might be completely different from that of other TOG domains. We find that TOG2-S shows some preference for GMPCPP MTs and to a region overlapping with the GTP or GDP-Pi cap, and is enriched at rapidly growing MT ends that are expected to have a long cap. Therefore, it is possible that, similar to EBs, TOG2 binds to a site that overlaps with interprotofilament contacts, the structure of which is known to be sensitive to GTP hydrolysis by tubulin (Maurer et al., 2012). The fact that TOG2-S accumulation at MT tips is reduced in the presence of EB3ΔTail, possibly due to structural changes in the cap region or through direct competition, supports this view, which would need to be tested by structural approaches. It is important to emphasize that since the enrichment of TOG2-S at the growing MT ends is only observed at high concentrations, TOG2 affinity for the stabilizing cap is low, and at lower concentrations, TOG2 requires other domains or proteins, such as EBs, to be positioned in this region.

CLASPs prevent catastrophe onset but do not slow down MT shortening (Lawrence et al., 2018; this paper). This finding combined with the experiments showing TOG2-S enrichment behind the outmost tip at the MT plus end suggests that CLASPs, through their TOG2 domain, promote the stability of the MT region corresponding to the GTP (or GDP-Pi) cap so long as it is present. Recent work showed that the onset of an MT catastrophe occurs with a delay, which is visible as an MT pause or a very slow depolymerization that corresponds to the gradual loss of MT-stabilizing EB1-binding sites within the cap (Duellberg et al., 2016a). Since only the density of such stabilizing sites at the outmost end of the cap is critical (Duellberg et al., 2016a), this would explain how a small number of CLASP molecules can be sufficient to prevent catastrophes. This idea also fits with the data that CLASP prevents short depolymerization excursions and that TOG2-S can induce MT pausing, during which some EB-binding sites are still present at MT ends. The number of such stabilizing sites at the pausing MT ends is lower than at growing ends, explaining why TOG2-S, which has a low affinity to these sites, is not enriched at pausing ends. For this reason, in order to induce pausing, TOG2-S concentration must be sufficiently high to decorate the whole MT. Recent work showed that CLASP2 does not affect the length of the GTP cap (Lawrence et al., 2018), which suggests that CLASPs stabilize MT ends directly rather than by regulating GTP hydrolysis by tubulin.

An important clue about the catastrophe-suppressing mechanism is provided by the finding that, in the presence of CLASPs, persistently growing MTs could tolerate the loss of a significant number of protofilaments at the growing end. Recent work suggested that MT tapering and the accompanying reduction in the stabilizing cap density might be the underlying cause of MT age-induced catastrophes (Coombes et al., 2013; Duellberg et al., 2016b). Interestingly, while in the presence of CLASP2 or TOG2-S, catastrophes were almost completely blocked, we observed a ~3-fold increase in the frequency of tip repair events, in which a transient MT growth perturbation and protofilament loss was followed by the appearance of a rear, catching up comet that restored the complete MT plus end structure. Our previous work indicated that such events occur when at least one MT protofilament is transiently blocked but can later resume elongation (Doodhi et al., 2016). The frequency of tip repair events in the presence of CLASP2 (~0.2 per min) was lower than the catastrophe frequency in control assays (~0.5 per min), which means that these events could represent at least a fraction of the suppressed catastrophes. However, we note that the detection of tip repair events is limited by the resolution of optical microscopy, and might thus be underestimated, and the contribution of such events to MT growth persistence could in fact be significant. Furthermore, the presence of very long (up to 4 μm) tip repair events shows that CLASPs can prevent disassembly of stabilizing caps on partial MT structures and thus create a window of opportunity for MT tip restoration.

While TOG2 is key for catastrophe suppression, TOG3 did not suppress catastrophes but mildly promoted rescues together with EB3. The distinct activities of TOG2 and TOG3 suggest that catastrophe suppression and rescue induction might be mechanistically different, and, in line with this view, more CLASP2α molecules were needed to suppress catastrophes than to induce rescues.

Many cytoskeletal proteins are known to be controlled by auto-inhibitory interactions that are released by partner binding. Here, we showed that CLASPs share this property, because their C-terminal domain (CLIP-ID), responsible for the interactions with the majority of known CLASP partners such as CLIP-170 (Akhmanova et al., 2001; Efimov et al., 2007; Hannak and Heald, 2006; Lansbergen et al., 2006), can inhibit the MT-directed activities of TOG2 and possibly TOG3. The N-terminal TOG domain of CLASP2α, TOG1, which does not bind to either MTs or free tubulin because it lacks the conserved residues necessary for contacting tubulin, can release this auto-inhibition. TOG1 is present in CLASP1/2α but absent in CLASP2β and CLASP2γ isoforms (Akhmanova et al., 2001). CLASP2 can thus be expressed both as a constitutively active and regulated isoforms, which might be important for controlling CLASP activity at specific cellular sites.

Our findings provide insight into how CLASPs work in different cellular settings, for example, by helping MTs to withstand

compression when they make sharp turns in plant cells (Ambrose et al., 2011) or in the protrusions of cancer cells invading a 3D matrix (Bouchet et al., 2016). The finding that the CLASP-EB complex strongly lowered the kinetic threshold for template-based MT outgrowth explains why CLASPs can stimulate γ-tubulin-dependent MT nucleation from the Golgi (Efimov et al., 2007). Both TOG2 and TOG3 are likely to cooperate in this process, as they both display partial activity compared with the full-length CLASP2α protein. Taken together, our data reveal how a combination of distinct domains with anti-catastrophe, rescue, partner binding, and autoregulatory activities make CLASPs potent MT regulators that help to maintain MTs in a growing state.

STAR★METHODS

Detailed methods are provided in the online version of this paper and include the following:

- KEY RESOURCES TABLE
- CONTACT FOR REAGENT AND RESOURCE SHARING
- EXPERIMENTAL MODEL AND SUBJECT DETAILS
- METHOD DETAILS
  - DNA Constructs, Cell Lines and Cell Culture
  - Protein Purification from HEK293T Cells for *In Vitro* Reconstitution Assays
  - Cloning, Protein Expression and Purification from *E. coli*
  - MT Pelleting Assay
  - Isothermal Titration Calorimetry (ITC)
  - Size Exclusion Chromatography Followed by Multi-angle Light Scattering (SEC-MALS)
  - Crystallization, Data Collection and Structure Solution
  - *In Vitro* MT Dynamics Assays
  - TIRF Microscopy
  - *In Vitro* Template-based MT Outgrowth Assay
  - Microfabrication of SiO_2 Barriers
  - MT Growth against SiO_2 Barriers and Analysis of Barrier Contact Events
  - Quantification of the Intensities of EB Comets
  - Tip-averaging of MT Intensity Profiles
  - Fitting and Averaging of GFP-TOG2-S Intensity Profiles
  - End Tapering Simulations
  - Analysis of MT Growth Variability
  - Single-molecule Fluorescence Intensity Analysis of CLASP2α
  - CLASP Molecule Counting at MT Tips and Rescue Points
- QUANTIFICATION AND STATISTICAL ANALYSIS
- DATA AND SOFTWARE AVAILABILITY

SUPPLEMENTAL INFORMATION

Supplemental Information includes seven figures, one table, and five videos and can be found with this article online at https://doi.org/10.1016/j.devcel.2018.05.032.

ACKNOWLEDGMENTS

We thank S. Royle for the gift of ch-TOG construct and the beamline scientists at beamline X06DA of the Swiss Light Source (Paul Scherrer Institut, Villigen, Switzerland) for technical assistance with the X-ray data collection. This work was supported by the European Research Council Synergy grant 609822 to M.D. and A. Akhmanova, by grant 31003A_166608 from the Swiss National Science Foundation to M.O.S., by an EMBO Long Term Fellowship to A.S., and by the Netherlands Organization for Scientific Research (NWO) ALW-Vidi grant 864.12.008 to L.C.K.

AUTHOR CONTRIBUTIONS

A. Aher, M.K., A.S., L.C.K., M.O.S., M.D., and A. Akhmanova designed experiments, analyzed data, and wrote the paper. A. Aher, M.K., A.S., A.R.,N.O., and R.R.-G. performed experiments and data analysis, T.W. and V.O. contributed to solving TOG1 structure, and E.A.K. carried out data analysis and modeling. A. Akhmanova coordinated the project.

DECLARATION OF INTERESTS

The authors declare no competing financial interests.

Received: October 19, 2017
Revised: April 13, 2018
Accepted: May 25, 2018
Published: June 21, 2018

REFERENCES

Adams, P.D., Afonine, P.V., Bunkoczi, G., Chen, V.B., Davis, I.W., Echols, N., Headd, J.J., Hung, L.W., Kapral, G.J., Grosse-Kunstleve, R.W., et al. (2010). PHENIX: a comprehensive Python-based system for macromolecular structure solution. Acta Crystallogr. D Biol. Crystallogr. *66*, 213–221.

Akhmanova, A., Hoogenraad, C.C., Drabek, K., Stepanova, T., Dortland, B., Verkerk, T., Vermeulen, W., Burgering, B.M., De Zeeuw, C.I., Grosveld, F., and Galjart, N. (2001). Clasps are CLIP-115 and -170 associating proteins involved in the regional regulation of microtubule dynamics in motile fibroblasts. Cell *104*, 923–935.

Akhmanova, A., and Steinmetz, M.O. (2015). Control of microtubule organization and dynamics: two ends in the limelight. Nat. Rev. Mol. Cell Biol. *16*, 711–726.

Al-Bassam, J., and Chang, F. (2011). Regulation of microtubule dynamics by TOG-domain proteins XMAP215/Dis1 and CLASP. Trends Cell Biol. *21*, 604–614.

Al-Bassam, J., Kim, H., Brouhard, G., van Oijen, A., Harrison, S.C., and Chang, F. (2010). CLASP promotes microtubule rescue by recruiting tubulin dimers to the microtubule. Dev. Cell *19*, 245–258.

Ambrose, C., Allard, J.F., Cytrynbaum, E.N., and Wasteneys, G.O. (2011). A CLASP-modulated cell edge barrier mechanism drives cell-wide cortical microtubule organization in *Arabidopsis*. Nat. Commun. *2*, 430.

Bieling, P., Laan, L., Schek, H., Munteanu, E.L., Sandblad, L., Dogterom, M., Brunner, D., and Surrey, T. (2007). Reconstitution of a microtubule plus-end tracking system in vitro. Nature *450*, 1100–1105.

Bouchet, B.P., Noordstra, I., van Amersfoort, M., Katrukha, E.A., Ammon, Y.C., Ter Hoeve, N.D., Hodgson, L., Dogterom, M., Derksen, P.W., and Akhmanova, A. (2016). Mesenchymal cell invasion requires cooperative regulation of persistent microtubule growth by SLAIN2 and CLASP1. Dev. Cell *39*, 708–723.

Bratman, S.V., and Chang, F. (2007). Stabilization of overlapping microtubules by fission yeast CLASP. Dev. Cell *13*, 812–827.

Brouhard, G.J. (2015). Dynamic instability 30 years later: complexities in microtubule growth and catastrophe. Mol. Biol. Cell *26*, 1207–1210.

Brouhard, G.J., Stear, J.H., Noetzel, T.L., Al-Bassam, J., Kinoshita, K., Harrison, S.C., Howard, J., and Hyman, A.A. (2008). XMAP215 is a processive microtubule polymerase. Cell *132*, 79–88.

Campbell, J.N., and Slep, K.C. (2011). alphabeta-Tubulin and microtubule-binding assays. Methods Mol. Biol. *777*, 87–97.

Chazeau, A., Katrukha, E.A., Hoogenraad, C.C., and Kapitein, L.C. (2016). Studying neuronal microtubule organization and microtubule-associated

proteins using single molecule localization microscopy. Methods Cell Biol. *131*, 127–149.

Chen, V.B., Arendall, W.B., 3rd, Headd, J.J., Keedy, D.A., Immormino, R.M., Kapral, G.J., Murray, L.W., Richardson, J.S., and Richardson, D.C. (2010). MolProbity: all-atom structure validation for macromolecular crystallography. Acta Crystallogr. D Biol. Crystallogr. *66*, 12–21.

Coombes, C.E., Yamamoto, A., Kenzie, M.R., Odde, D.J., and Gardner, M.K. (2013). Evolving tip structures can explain age-dependent microtubule catastrophe. Curr. Biol. *23*, 1342–1348.

Cowtan, K. (2006). The Buccaneer software for automated model building. 1. Tracing protein chains. Acta Crystallogr. D Biol. Crystallogr. *62*, 1002–1011.

Desai, A., and Mitchison, T.J. (1997). Microtubule polymerization dynamics. Annu. Rev. Cell Dev. Biol. *13*, 83–117.

Doodhi, H., Prota, A.E., Rodriguez-Garcia, R., Xiao, H., Custar, D.W., Bargsten, K., Katrukha, E.A., Hilbert, M., Hua, S., Jiang, K., et al. (2016). Termination of protofilament elongation by Eribulin induces lattice defects that promote microtubule catastrophes. Curr. Biol. *26*, 1713–1721.

Drabek, K., van Ham, M., Stepanova, T., Draegestein, K., van Horssen, R., Sayas, C.L., Akhmanova, A., Ten Hagen, T., Smits, R., Fodde, R., et al. (2006). Role of CLASP2 in microtubule stabilization and the regulation of persistent motility. Curr. Biol. *16*, 2259–2264.

Duellberg, C., Cade, N.I., Holmes, D., and Surrey, T. (2016a). The size of the EB cap determines instantaneous microtubule stability. Elife *5*, e13470.

Duellberg, C., Cade, N.I., and Surrey, T. (2016b). Microtubule aging probed by microfluidics-assisted tubulin washout. Mol. Biol. Cell *27*, 3563–3573.

Efimov, A., Kharitonov, A., Efimova, N., Loncarek, J., Miller, P.M., Andreyeva, N., Gleeson, P., Galjart, N., Maia, A.R., McLeod, I.X., et al. (2007). Asymmetric CLASP-dependent nucleation of noncentrosomal microtubules at the trans-Golgi network. Dev. Cell *12*, 917–930.

Emsley, P., and Cowtan, K. (2004). Coot: model-building tools for molecular graphics. Acta Crystallogr. D Biol. Crystallogr. *60*, 2126–2132.

Gardner, M.K., Charlebois, B.D., Janosi, I.M., Howard, J., Hunt, A.J., and Odde, D.J. (2011a). Rapid microtubule self-assembly kinetics. Cell *146*, 582–592.

Gardner, M.K., Zanic, M., Gell, C., Bormuth, V., and Howard, J. (2011b). The depolymerizing kinesins Kip3 (kinesin-8) and MCAK (kinesin-13) are catastrophe factors that destabilize microtubules by different mechanisms. Cell *147*, 1092–1103.

Gardner, M.K., Zanic, M., and Howard, J. (2013). Microtubule catastrophe and rescue. Curr. Opin. Cell Biol. *25*, 14–22.

Grimaldi, A.D., Maki, T., Fitton, B.P., Roth, D., Yampolsky, D., Davidson, M.W., Svitkina, T., Straube, A., Hayashi, I., and Kaverina, I. (2014). CLASPs are required for proper microtubule localization of end-binding proteins. Dev. Cell *30*, 343–352.

Hannak, E., and Heald, R. (2006). Xorbit/CLASP links dynamic microtubules to chromosomes in the *Xenopus* meiotic spindle. J. Cell Biol. *172*, 19–25.

Honnappa, S., Gouveia, S.M., Weisbrich, A., Damberger, F.F., Bhavesh, N.S., Jawhari, H., Grigoriev, I., van Rijssel, F.J., Buey, R.M., Lawera, A., et al. (2009). An EB1-binding motif acts as a microtubule tip localization signal. Cell *138*, 366–376.

Howard, J., and Hyman, A.A. (2009). Growth, fluctuation and switching at microtubule plus ends. Nat. Rev. Mol. Cell Biol. *10*, 569–574.

Janson, M.E., de Dood, M.E., and Dogterom, M. (2003). Dynamic instability of microtubules is regulated by force. J. Cell Biol. *161*, 1029–1034.

Kabsch, W. (2010a). Integration, scaling, space-group assignment and post-refinement. Acta Crystallogr. D Biol. Crystallogr. *66*, 133–144.

Kabsch, W. (2010b). Xds. Acta Crystallogr. D Biol. Crystallogr. *66*, 125–132.

Kalisch, S.M., Laan, L., and Dogterom, M. (2011). Force generation by dynamic microtubules in vitro. Methods Mol. Biol. *777*, 147–165.

Lacroix, B., Bourdages, K.G., Dorn, J.F., Ihara, S., Sherwood, D.R., Maddox, P.S., and Maddox, A.S. (2014). In situ imaging in *C. elegans* reveals developmental regulation of microtubule dynamics. Dev. Cell *29*, 203–216.

Lansbergen, G., Grigoriev, I., Mimori-Kiyosue, Y., Ohtsuka, T., Higa, S., Kitajima, I., Demmers, J., Galjart, N., Houtsmuller, A.B., Grosveld, F., and Akhmanova, A. (2006). CLASPs attach microtubule plus ends to the cell cortex through a complex with LL5beta. Dev. Cell *11*, 21–32.

Lawrence, E.J., Arpag, G., Norris, S.R., and Zanic, M. (2018). Human CLASP2 specifically regulates microtubule catastrophe and rescue. Mol. Biol. Cell *29*, 1168–1177.

Leano, J.B., Rogers, S.L., and Slep, K.C. (2013). A cryptic TOG domain with a distinct architecture underlies CLASP-dependent bipolar spindle formation. Structure *21*, 939–950.

Maiato, H., Fairley, E.A., Rieder, C.L., Swedlow, J.R., Sunkel, C.E., and Earnshaw, W.C. (2003). Human CLASP1 is an outer kinetochore component that regulates spindle microtubule dynamics. Cell *113*, 891–904.

Maiato, H., Khodjakov, A., and Rieder, C.L. (2005). *Drosophila* CLASP is required for the incorporation of microtubule subunits into fluxing kinetochore fibres. Nat. Cell Biol. *7*, 42–47.

Maki, T., Grimaldi, A.D., Fuchigami, S., Kaverina, I., and Hayashi, I. (2015). CLASP2 has two distinct TOG domains that contribute differently to microtubule dynamics. J. Mol. Biol. *427*, 2379–2395.

Maton, G., Edwards, F., Lacroix, B., Stefanutti, M., Laband, K., Lieury, T., Kim, T., Espeut, J., Canman, J.C., and Dumont, J. (2015). Kinetochore components are required for central spindle assembly. Nat. Cell Biol. *17*, 953.

Maurer, S.P., Cade, N.I., Bohner, G., Gustafsson, N., Boutant, E., and Surrey, T. (2014). EB1 accelerates two conformational transitions important for microtubule maturation and dynamics. Curr. Biol. *24*, 372–384.

Maurer, S.P., Fourniol, F.J., Bohner, G., Moores, C.A., and Surrey, T. (2012). EBs recognize a nucleotide-dependent structural cap at growing microtubule ends. Cell *149*, 371–382.

Mimori-Kiyosue, Y. (2011). Shaping microtubules into diverse patterns: molecular connections for setting up both ends. Cytoskeleton (Hoboken) *68*, 603–618.

Mimori-Kiyosue, Y., Grigoriev, I., Lansbergen, G., Sasaki, H., Matsui, C., Severin, F., Galjart, N., Grosveld, F., Vorobjev, I., Tsukita, S., and Akhmanova, A. (2005). CLASP1 and CLASP2 bind to EB1 and regulate microtubule plus-end dynamics at the cell cortex. J. Cell Biol. *168*, 141–153.

Mohan, R., Katrukha, E.A., Doodhi, H., Smal, I., Meijering, E., Kapitein, L.C., Steinmetz, M.O., and Akhmanova, A. (2013). End-binding proteins sensitize microtubules to the action of microtubule-targeting agents. Proc. Natl. Acad. Sci. USA *110*, 8900–8905.

Montenegro Gouveia, S., Leslie, K., Kapitein, L.C., Buey, R.M., Grigoriev, I., Wagenbach, M., Smal, I., Meijering, E., Hoogenraad, C.C., Wordeman, L., et al. (2010). In vitro reconstitution of the functional interplay between MCAK and EB3 at microtubule plus ends. Curr. Biol. *20*, 1717–1722.

Moriwaki, T., and Goshima, G. (2016). Five factors can reconstitute all three phases of microtubule polymerization dynamics. J. Cell Biol. *215*, 357–368.

Olieric, N., Kuchen, M., Wagen, S., Sauter, M., Crone, S., Edmondson, S., Frey, D., Ostermeier, C., Steinmetz, M.O., and Jaussi, R. (2010). Automated seamless DNA co-transformation cloning with direct expression vectors applying positive or negative insert selection. BMC Biotechnol. *10*, 56.

Ruan, Y., and Wasteneys, G.O. (2014). CLASP: a microtubule-based integrator of the hormone-mediated transitions from cell division to elongation. Curr. Opin. Plant Biol. *22*, 149–158.

Ruhnow, F., Zwicker, D., and Diez, S. (2011). Tracking single particles and elongated filaments with nanometer precision. Biophys. J. *100*, 2820–2828.

Schindelin, J., Arganda-Carreras, I., Frise, E., Kaynig, V., Longair, M., Pietzsch, T., Preibisch, S., Rueden, C., Saalfeld, S., Schmid, B., et al. (2012). Fiji: an open-source platform for biological-image analysis. Nat. Methods *9*, 676–682.

Sen, I., Veprintsev, D., Akhmanova, A., and Steinmetz, M.O. (2013). End binding proteins are obligatory dimers. PLoS One *8*, e74448.

Sharma, A., Aher, A., Dynes, N.J., Frey, D., Katrukha, E.A., Jaussi, R., Grigoriev, I., Croisier, M., Kammerer, R.A., Akhmanova, A., et al. (2016). Centriolar CPAP/SAS-4 imparts slow processive microtubule growth. Dev. Cell *37*, 362–376.

Sheldrick, G.M. (2010). Experimental phasing with SHELXC/D/E: combining chain tracing with density modification. Acta Crystallogr. D Biol. Crystallogr. *66*, 479–485.

Sousa, A., Reis, R., Sampaio, P., and Sunkel, C.E. (2007). The *Drosophila* CLASP homologue, Mast/Orbit regulates the dynamic behaviour of interphase microtubules by promoting the pause state. Cell Motil. Cytoskeleton *64*, 605–620.

Taylor, J.R. (1997). An Introduction to Error Analysis (Sausalito, University Science Books).

van Riel, W.E., Rai, A., Bianchi, S., Katrukha, E.A., Liu, Q., Heck, A.J., Hoogenraad, C.C., Steinmetz, M.O., Kapitein, L.C., and Akhmanova, A. (2017). Kinesin-4 KIF21B is a potent microtubule pausing factor. Elife *6*, e24746.

Wieczorek, M., Bechstedt, S., Chaaban, S., and Brouhard, G.J. (2015). Microtubule-associated proteins control the kinetics of microtubule nucleation. Nat. Cell Biol. *17*, 907–916.

Yu, N., Signorile, L., Basu, S., Ottema, S., Lebbink, J.H., Leslie, K., Smal, I., Dekkers, D., Demmers, J., and Galjart, N. (2016). Isolation of functional tubulin dimers and of tubulin-associated proteins from mammalian cells. Curr. Biol. *26*, 1728–1736.

Zaliapin, I., Semenova, I., Kashina, A., and Rodionov, V. (2005). Multiscale trend analysis of microtubule transport in melanophores. Biophys. J. *88*, 4008–4016.

Zanic, M., Widlund, P.O., Hyman, A.A., and Howard, J. (2013). Synergy between XMAP215 and EB1 increases microtubule growth rates to physiological levels. Nat. Cell Biol. *15*, 688–693.

STAR★METHODS

KEY RESOURCES TABLE

REAGENT or RESOURCE	SOURCE	IDENTIFIER
Bacterial and Virus Strains		
E.coli BL21 (DE3)	Agilent	200131
Chemicals, Peptides, and Recombinant Proteins		
StrepTactin Sepharose High Performance	GE Healthcare	Cat# 28-9355-99
Polyethyleneimine	Polysciences	Cat# 24765-2
cOmplete™, EDTA-free Protease Inhibitor Cocktail	Roche	Cat# 4693116001
Tubulin Porcine	Cytoskeleton	Cat# T240-C
Tubulin Porcine TRITC	Cytoskeleton	Cat# TL590M
Tubulin Porcine HiLyte 488™	Cytoskeleton	Cat# TL488M
Tubulin Porcine HiLyte 647™	Cytoskeleton	Cat# TL670M
Tubulin Porcine	Cytoskeleton	Cat# T333P
GMPCPP	Jena Biosciences	Cat# NU-405L
GTP	Sigma	Cat# G8877
Glucose oxidase	Sigma	Cat# G7141
Catalase	Sigma	Cat# C9322
DTT	Sigma	Cat# R0861
k-casein	Sigma	Cat# C0406
Neutravidin	Invitrogen	Cat# A-2666
Taxol	Sigma	Cat# T7402
Colchicine	Sigma	Cat# C9754
Vinblastine sulfate salt	Sigma	Cat# V1377
Dolastatin	Sigma	Cat# D5566
Strep-GFP-CLASP2α full length	This study	N/A
Strep-GFP-CLASP1α full length	This study	N/A
Strep-GFP-CLASP2α IPNN	This study	N/A
Strep-GFP-CLASP2α 1-1176 (ΔCLIP-ID)	This study	N/A
Strep-GFP-CLASP2α 1-813 (TOG12-S)	This study	N/A
Strep-GFP-CLASP2α 734-1527 (S-TOG3-CLIP-ID)	This study	N/A
Strep-GFP-CLASP2α 734-1209 (S-TOG3)	This study	N/A
Strep-GFP-CLASP2α 734-813$^{\Delta}$1175-1527 (S-CLIP-ID)	This study	N/A
Strep-GFP-CLASP2α 1-261$^{\Delta}$524-1527 (ΔTOG2)	This study	N/A
Strep-GFP-CLASP2α 1-260$^{\Delta}$585-813 (TOG1-S)	This study	N/A
Strep-GFP-CLASP2α 261-813 (L-TOG2-S)	This study	N/A
Strep-GFP-CLASP2α 261-813 W339E (L-TOG2-S W339E)	This study	N/A
Strep-GFP-CLASP2α 261-813 R462E (L-TOG2-S R462E)	This study	N/A
Strep-GFP-CLASP2α 261-813 R504E (L-TOG2-S R504E)	This study	N/A
Strep-GFP-CLASP2α 295-813 (TOG2-S)	This study	N/A
Strep-GFP-CLASP2α 295-673 $^{\Delta}$EB3 1-200 (TOG2-EB3CH)	This study	N/A
Strep-GFP-chTOG 1-233$^{\Delta}$CLASP2α 601-813 (chTOG-TOG1-S)	This study	N/A
Strep-GFP-chTOG 262-495 $^{\Delta}$CLASP2α 601-813 (chTOG-TOG2-S)	This study	N/A
Strep-GFP-CLASP2α 734-813 (S)	This study	N/A
Strep-GFP-CLASP2α 281-1527 (ΔTOG1)	This study	N/A
Strep-GFP-CLASP2α 295-1149 (TOG2-S-TOG3)	This study	N/A
Strep-GFP-CLASP2α 295-813$^{\Delta}$1119-1527 (TOG2-S-CLIP-ID)	This study	N/A
Strep-GFP-CLASP2α 1-813$^{\Delta}$1119-1527(TOG1TOG2-S-CLIP-ID)	This study	N/A

(*Continued on next page*)

Continued		
REAGENT or RESOURCE	SOURCE	IDENTIFIER
Strep-GFP-MACF2 5455-5497$^{\wedge}$CLIP170 334-1232 (SxIP$_{MACF}$-CC$_{CLIP170}$)	This study	N/A
Strep-GFP-MCAK full length	This study	N/A
TOG1	This study	N/A
TOG2	This study	N/A
TOG3	This study	N/A
CLIP-ID	This study	N/A
mCherry-EB3 full length	Montenegro Gouveia et al., 2010	N/A
mCherry-EB3 ΔTail	Montenegro Gouveia et al., 2010	N/A
GFP-EB3 full length	Montenegro Gouveia et al., 2010	N/A
Deposited Data		
Atomic coordinates and structure factors	PDBe	5NR4
Experimental Models: Cell Lines		
Human: HEK293T	ATCC	CRL-11268
Monkey: COS7	ATCC	CRL-1651
MDA-MB231 EB3GFP	Bouchet et al., 2016	N/A
Oligonucleotides		
siRNA targeting sequence: CLASP1# A: GCCATTATGCCAACTATCT	Mimori-Kiyosue et al., 2005	N/A
siRNA targeting sequence: CLASP2# A: GTTCAGAAAGCCCTTGATG	Mimori-Kiyosue et al., 2005	N/A
Recombinant DNA		
Strep-GFP-CLASP2α 734-813 (S)	This study	N/A
Strep-GFP-CLASP2α 1-813 (TOG12-S)	This study	N/A
Strep-GFP-CLASP2α 1-260$^{\wedge}$585-813 (TOG1-S)	This study	N/A
Strep-GFP-CLASP2α 261-813 (L-TOG2-S)	This study	N/A
Strep-GFP-CLASP2α 261-813 R462E (L-TOG2-S R462E)	This study	N/A
Software and Algorithms		
ImageJ	NIH	https://imagej.nih.gov/ij/
Metamorph	Molecular Devices	https://www.moleculardevices.com/products/cellular-imaging-systems/acquisition-and-analysis-software/metamorph-microscopy
Matlab	Mathworks	https://www.mathworks.com/
GraphPad Prism	GraphPad	https://www.graphpad.com/scientific-software/prism/
KymoResliceWide plugin	Eugene Katrukha	https://github.com/ekatrukha/KymoResliceWide
Multiscale Trend Analysis Matlab code	Eugene Katrukha	https://github.com/ekatrukha/MTA
Matlab code for Figures 6, 7, and S5	This study	https://doi.org/10.6084/m9.figshare.6260732

CONTACT FOR REAGENT AND RESOURCE SHARING

Further information and requests for resources and reagents should be directed to and will be fulfilled by the Lead Contact, Anna Akhmanova (a.akhmanova@uu.nl).

EXPERIMENTAL MODEL AND SUBJECT DETAILS

E.coli expression strain BL21 (DE3) was used for recombinant expression of individual CLASP2α TOG domains used for MT pelleting, tubulin binding, biophysical and X-ray crystallography experiments. The CLASP2-TOG-EB3CH protein was also purified from *E.coli* BL21 (DE3). The cells were cultured in standard LB medium.

All the other CLASP, chTOG, MCAK full length, truncations and fusion constructs were overexpressed in HEK293T cells for purification. MDA-MB-231 cells stably expressing EB3-GFP (Bouchet et al., 2016) were cultured in DMEM supplemented with 10% FCS. COS-7 and HEK 293T cells were cultured in DMEM/F10 (1:1 ratio, Lonza, Basel, Switzerland) supplemented with 10% FCS, both grown at 37°C. HEK293T, MDA-MB-231 and COS-7 cell lines used here were not found in the database of commonly misidentified cell lines maintained by ICLAC and NCBI BioSample, were not authenticated and were negative for mycoplasma contamination.

METHOD DETAILS

DNA Constructs, Cell Lines and Cell Culture

CLASP truncations expressed in mammalian cells were made from the full length constructs described previously (Akhmanova et al., 2001; Mimori-Kiyosue et al., 2005) in modified pEGFP-C1 or pmCherry-C1 vectors with a StrepII tag. Ch-TOG construct was a gift of S. Royle (University of Warwick, UK). For siRNA transfection, MDA-MB-231 cells stably expressing EB3-GFP were simultaneously treated with siRNAs specific for CLASP1 and CLASP2 (Mimori-Kiyosue et al., 2005) or with control (luciferase) siRNA (Bouchet et al., 2016) for 72 hours. EB3-GFP comets were imaged in live cells on a TIRF microscope and kymographs were analyzed to determine the effects of CLASP1/2 depletion on MT plus end dynamics. For overexpression of CLASP2 constructs, COS-7 cells were transiently transfected with different StrepII-GFP-CLASP2 constructs (as indicated in the figures) for 12 hours, and the GFP signals of these constructs were used to quantify MT dynamics. Live imaging of COS-7 cells overexpressing different constructs of CLASP2 in the presence of colchicine (250 nM) was performed within 40 min of colchicine treatment.

Protein Purification from HEK293T Cells for *In Vitro* Reconstitution Assays

GFP-CLASP1α, mCherry-CLASP2α, Tag-BFP-CLASP2α, GFP-CLASP2α, its TOG-domain truncations, point mutants, fusion proteins with the TOG domains of chTOG and coiled coil of CLIP-170, and GFP-MCAK used in the *in vitro* reconstitutions assays were purified from HEK293T cells using the Strep(II)-streptactin affinity purification. Cells were harvested 2 days after transfection. Cells from a 15 cm dish were lysed in 500 μl of lysis buffer (50 mM HEPES, 300 mM NaCl and 0.5% Triton X-100, pH 7.4) supplemented with protease inhibitors (Roche) on ice for 15 minutes. The supernatant obtained from the cell lysate after centrifugation at 21,000 x g for 20 minutes was incubated with 40 μl of StrepTactin Sepharose beads (GE) for 45 minutes. The beads were washed 3 times in the lysis buffer without the protease inhibitors. The protein was eluted with 40 μl of elution buffer (50 mM HEPES, 150 mM NaCl, 1 mM $MgCl_2$, 1 mM EGTA, 1 mM dithiothreitol (DTT), 2.5 mM d-Desthiobiotin and 0.05% Triton X-100, pH 7.4). Purified proteins were snap-frozen and stored at −80°C.

Cloning, Protein Expression and Purification from *E. coli*

Individual domains of CLASP2 (Figure S2A, rightmost panel) were cloned into a pET-based bacterial expression vector using the restriction free positive selection method (Olieric et al., 2010). All recombinant proteins contained either an N-terminal thioredoxin-6xHis or 6xHis cleavable tag for affinity purification. For standard expression, the proteins were transformed into the *E. coli* expression strain BL21(DE3). Transformed cells were cultivated in LB at 37°C until an OD_{600} between 0.4 to 0.6 was reached. The cultures were subsequently cooled down to 20°C prior to induction with 0.4 mM isopropyl 1-thio-β-galactopyranoside (IPTG, Sigma). Expression was carried out overnight at 20°C. Cells were harvested by centrifugation at 4°C for 15-20 min and lysed by sonication (50 mM HEPES, pH 8.0, 500 mM NaCl, 10 mM Imidazole, 10% Glycerol, 2 mM β-mercaptoethanol, proteases inhibitors (Roche)). The crude extracts were cleared by centrifugation at 20,000 x g for 20 min and the supernatants were filtered through a 0.45 micron filter before purification.

TOG domain proteins were purified by immobilized metal-affinity chromatography (IMAC) on HisTrap HP Ni^{2+} Sepharose columns (GE Healthcare) at 4°C according to the manufacturer's instructions. The thioredoxin-6xHis or 6xHis tags were cleaved by 3C protease during dialysis against lysis buffer (without proteases inhibitors). Cleaved samples were reapplied onto an IMAC column to separate the cleaved products from the respective tags and potentially uncleaved protein. Processed proteins were concentrated and gel filtrated on a HiLoad Superdex 75 16/60 size exclusion chromatography column (GE Healthcare) equilibrated in 20 mM Tris HCl, pH 7.5, 150 mM NaCl, 2 mM DTT. Protein fractions were analyzed by Coomasie stained SDS-PAGE. Fractions containing the target protein were pooled and concentrated by ultrafiltration. Protein concentrations were estimated by UV at 280 nm and the pure proteins were aliquoted, flash frozen in liquid nitrogen and stored at −80°C.

MT Pelleting Assay

MT pelleting assays were performed as previously described (Campbell and Slep, 2011). Briefly, taxol-stabilized MTs were assembled in BRB80 buffer (80 mM PIPES-KOH, pH 6.8, 1 mM $MgCl_2$, 1 mM EGTA) from pure bovine brain tubulin at 1 mg/mL. 50 μL of polymerized MTs were incubated 20 min with 20 μL of the protein of interest at 2 mg/mL (diluted to the desired protein concentration with 2 x BRB80 buffer) and 50 μL of BRB80. The mixture was centrifuged at 25°C for 20 min at 180,000 x g. Mix, supernatant and pellet fractions were analyzed by Coomasie stained 12% SDS-PAGE. As controls, MTs alone and individual TOG domains were processed the same way.

Isothermal Titration Calorimetry (ITC)

All the proteins were buffer exchanged to BRB80 buffer supplemented with 50 mM NaCl by overnight dialysis at 4°C. ITC experiments were performed at 25°C using an ITC200 system (Microcal) by step wise addition of different TOG domain proteins (syringe

concentration was 150 μM for CLASP2 TOG1, TOG2 and TOG3, 200 μM for CLIP-ID, and 250 μM for Stu2 TOG1) in the ITC cell containing 15 μM bovine brain tubulin. The resulting heats were integrated and fitted in Origin (OriginLab) using the standard 'one set of sites' model implemented in the software package. Only for Stu2 TOG1 the dissociation constant of binding to tubulin could be determined (K_d=18.8 ± 3.21 nM). TOG1-TOG2 interaction was probed using 227 μM of CLASP2 TOG1 in the syringe and 60 μM TOG2 in the cell). TOG2-CLIP-ID interaction was probed using 500 μM CLIP-ID in the syringe and 50 μM TOG2 in the cell.

Size Exclusion Chromatography Followed by Multi-angle Light Scattering (SEC-MALS)

SEC-MALS experiments were performed in 20 mM Tris-HCl, pH 7.5, 150 mM NaCl, 2 mM DTT) using a S-200 10/300 analytical size exclusion chromatography column connected in-line to a miniDAWN TREOS light scattering and Optilab T-rEX refractive index detector (Wyatt Technology). Measurements were carried out at 20°C and each sample was injected at 1 mg/ml (injected volume: 30 μl). Data analysis was performed using the software package provided by the instrument.

Crystallization, Data Collection and Structure Solution

CLASP2-TOG1 (HsCLASP2 residues 2-228) crystals were obtained by the hanging-drop vapor diffusion method at 20°C in the Morpheus crystallization condition B12 (Molecular Dimensions) by mixing 2 μL of the protein at 7 mg/mL with 2 μL of the reservoir solution. Crystals appeared over-night and were frozen directly in liquid nitrogen.

A single-wavelength anomalous diffraction experiment from intrinsic sulfur atoms (S-SAD) was performed at the macromolecular crystallography super-bending magnet beamline X06DA (PXIII) at the Swiss Light Source, Villigen, Switzerland. 360° native data sets were collected at 1.0 Å wavelength on a single crystal at 100 K. Multi-orientation 360° data were collected on the same crystal at 100 K at a wavelength of 2.066 Å with 0.2° oscillation and 0.1 sec exposure at 8 different orientations of multi-axis. The sample-to-detector distance was set to 120 mm. The data were processed using XDS (Kabsch, 2010b) and scaled and merged with XSCALE (Kabsch, 2010a).

Substructure determination and phasing were performed with SHELXC/D/E (Sheldrick, 2010). The successful SHELXD substructure solution that was found in a search for 20 sites had a CCall and a CCweak of 41.27 and 23.45, respectively. 154 cycles of density modification resulted in a clear separation of hands. Model building was performed using Bucaneer (Cowtan, 2006). The resulting model was improved through iterative model rebuilding in Coot (Emsley and Cowtan, 2004) and refined in the PHENIX software package (Adams et al., 2010). The quality of the structure was assessed with MolProbity (Chen et al., 2010). See Table S1 for crystallography data collection and refinement statistics. The structure was deposited in the PDB with the accession code 5NR4.

In Vitro MT Dynamics Assays

Doubly cycled GMPCPP MT seeds were prepared as described before (Mohan et al., 2013), by incubating tubulin mix containing 70% unlabeled porcine brain tubulin (Cytoskeleton), 18% biotin-tubulin (Cytoskeleton) and 12% rhodamine-tubulin (Cytoskeleton) at a total final tubulin concentration of 20 μM with 1 mM GMPCPP (Jena Biosciences) at 37°C for 30 minutes. MTs were pelleted by centrifugation in an Airfuge for 5 minutes at 119,000 × *g* and then depolymerized on ice for 20 minutes. This was followed by a second round of polymerization at 37°C with 1 mM GMPCPP. MT seeds were then pelleted as above and diluted 10 fold in MRB80 buffer containing 10% glycerol, snap frozen in liquid nitrogen and stored at −80°C.

Reconstitution of MT growth dynamics *in vitro* was performed as described previously (Montenegro Gouveia et al., 2010). Flow chambers, assembled from sticking plasma-cleaned glass coverslips onto microscopic slides with a double sided tape were functionalized by sequential incubation with 0.2 mg/ml PLL-PEG-biotin (Susos AG, Switzerland) and 1 mg/ml NeutrAvidin (Invitrogen) in MRB80 buffer (80 mM piperazine-*N*,*N*[prime]-bis(2-ethanesulfonic acid), pH 6.8, supplemented with 4 mM $MgCl_2$, and 1 mM EGTA. MT seeds were attached to the coverslip through biotin-NeutrAvidin interactions. Flow chambers were further blocked with 1 mg/ml κ-casein. The reaction mixture with or without CLASP proteins (MRB80 buffer supplemented with 15 μM porcine brain tubulin, 0.5 μM rhodamine-tubulin, 50 mM KCl, 1 mM guanosine triphosphate, 0.2 mg/ml κ-casein, 0.1% methylcellulose, and oxygen scavenger mix (50 mM glucose, 400 μg/ ml glucose oxidase, 200 μg/ml catalase, and 4 mM DTT)) was added to the flow chamber after centrifugation in an Airfuge for 5 minutes at 119,000 × *g*. For experiments in the presence of EB3, concentration of mCherry-EB3 or GFP-EB3 was as indicated in the figures and rhodamine-tubulin was excluded from the assay. The flow chamber was sealed with vacuum grease, and dynamic MTs were imaged immediately at 30°C using TIRF microscopy. All tubulin products were from Cytoskeleton Inc.

For experiments to test binding of TOG2-S to depolymerizing MTs, MT seeds were elongated in the presence of 20 μM porcine brain tubulin with 10% rhodamine-tubulin for 5 minutes in the buffer containing 50 mM KCl, 1 mM guanosine triphosphate, 0.2 mg/ml κ-casein, 0.1% methylcellulose, and oxygen scavenger mix (50 mM glucose, 400 μg/ ml glucose oxidase, 200 μg/ml catalase, and 4 mM DTT) in MRB80. Subsequently, stable GMPCPP caps were added by exchanging the reaction mixture to the one supplemented with 0.125 mM GMPCPP and 10% HiLyte647 labelled 5 μM porcine tubulin for 10 minutes. Finally, the GMPCPP-containing mixture was exchanged for a reaction mixture without tubulin, containing the indicated concentrations of GFP-TOG2-S (30 nM and 200 nM) and imaged for 30 minutes to observe depolymerizing MT ends.

TIRF Microscopy

In vitro reconstitution assays were imaged on a TIRF microscope setup as described previously (Mohan et al., 2013) or on an Ilas^2 TIRF setup. In brief, we used an inverted research microscope Nikon Eclipse Ti-E (Nikon) with the perfect focus system (Nikon), equipped with Nikon CFI Apo TIRF 100x 1.49 N.A. oil objective (Nikon) and controlled with MetaMorph 7.7.5 software (Molecular

Devices). The microscope was equipped with TIRF-E motorized TIRF illuminator modified by Roper Scientific France/PICT-IBiSA, Institut Curie. To keep the *in vitro* samples at 30°C, a stage top incubator model INUBG2E-ZILCS (Tokai Hit) was used. For excitation, 491 nm 100 mW Calypso (Cobolt) and 561 nm 100 mW Jive (Cobolt) lasers were used. We used ET-GFP 49002 filter set (Chroma) for imaging of proteins tagged with GFP or ET-mCherry 49008 filter set (Chroma) for imaging of proteins tagged with mCherry. Fluorescence was detected using an EMCCD Evolve 512 camera (Roper Scientific) with the intermediate lens 2.5X (Nikon C mount adapter 2.5X) or using the CoolSNAP HQ2 CCD camera (Roper Scientific) without an additional lens. In both cases the final magnification was 0.063 μm/pixel.

Ilas^2 system (Roper Scientific, Evry, FRANCE) is a dual laser illuminator for azimuthal spinning TIRF (or Hilo) illumination and with a custom modification for targeted photomanipulation. This system was installed on Nikon Ti microscope (with the perfect focus system, Nikon), equipped with 150 mW 488 nm laser and 100 mW 561 nm laser, 49002 and 49008 Chroma filter sets, EMCCD Evolve mono FW DELTA 512x512 camera (Roper Scientific) with the intermediate lens 2.5X (Nikon C mount adapter 2.5X), CCD camera CoolSNAP MYO M-USB-14-AC (Roper Scientific) and controlled with MetaMorph 7.8.8 software (Molecular Device). To keep the *in vitro* samples at 30°C, a stage top incubator model INUBG2E-ZILCS (Tokai Hit) was used. The final resolution using EMCCD camera was 0.065 μm/pixel, using CCD camera it was 0.045 μm/pixel.

In Vitro Template-based MT Outgrowth Assay

GMPCPP MT seeds labeled with HiLyte 488 tubulin were attached to the coverslips through biotin-neutravidin interaction as described above. After washing out unbound seeds, the flow chambers were blocked with 1 mg/ml κ-casein followed by the polymerization reaction mixture as above with different concentrations of Rhodamine-labeled tubulin. The nucleation probability was estimated as the fraction of the total GMPCPP seeds that showed MT outgrowth within 15 min imaging window. The nucleation probabilities over different tubulin concentration were fitted to the sigmoidal equation using GraphPad Prism 7.

Microfabrication of SiO_2 Barriers

Fabrication of the SiO_2 barriers was achieved in a cleanroom environment by subsequent deposition, lithography, and plasma etching steps, as previously described by (Kalisch et al., 2011). In short, the glass coverslips are first cleaned with a 70°C solution of base piranha (5:1:1 of H_2O:NH_4OH:H_2O_2) to remove any organic residues. To ensure a smooth surface for TIRF imaging, etching of the barriers must be done in pure SiO_2. To that end, we deposit a layer of 2 μm SiO_2 via Plasma-Enhanced Chemical Vapor Deposition with a deposition rate of 70 nm/min at 300°C (Oxford Instruments PlasmaPro 80). Then, a 2.2 μm layer of the positive resist S1813 (MicroChem) is spin coated at 1500 rpm on the coverslip and baked at 115°C for 90 seconds on a hotplate. Photo-lithography (EVG-620) with near-UV (13 mW/cm^2) through a chromium mask for 5 seconds transfers the barrier pattern into the resist. The sample is then developed in MF-321 (MicroPosit) for 60 seconds to remove the regions of UV-exposed resist. Next, Reactive Ion Etching (Leybold Hereaus) with a mixture of CHF_3:O_2 (50sccm:2.5sccm) ensures an anisotropic etch into the exposed SiO_2 with an etch rate of 33 nm/min. We made sure not to etch completely through the SiO_2 layer as the original surface of the glass coverslip is too rough for TIRF microscopy after etching. Finally, the remaining resist is removed and the sample cleaned in HNO_3 for 5 min. The final barriers were 10 μm wide and 1.7 μm high, enclosing channels with a width of 15 μm.

MT Growth against SiO_2 Barriers and Analysis of Barrier Contact Events

The micro-fabricated samples were passivated with PLL-PEG-biotin and κ-casein. Biotinylated GMPCPP-stabilized seeds were attached to the surface via streptavidin. The direction of flow of the seed mix was perpendicular to the barriers in order to favour perpendicular MT-barrier contact events. The height and straightness of the barriers in combination with methyl-cellulose in solution prevents MTs from growing over the barriers. The experiments without CLASP in solution were imaged on an Olympus TIRF microscope with a 60x, 1.45 NA oil immersion objective using an additional magnification of 1.6 to obtain 96x image magnification. Images were collected on two Andor iXon Ultra 897 EMCCD cameras for simultaneous dual-colour acquisition. The experiments with CLASP2α were imaged on a ilas^2 TIRF setup described in the TIRF microscopy section.

All MT-barrier contact events were separated into three different event types, i.e. sliding, stalling, and buckling. The contact angle of a MT with a barrier and the barrier contact times were determined and analyzed with a custom written MATLAB script, adapted from (Ruhnow et al., 2011). The catastrophe frequency was determined by counting the number of observed catastrophes and dividing this by the time a MT spends in contact with the barrier. The statistical error was obtained by dividing this number by the square root of the number of measured contact events. The growth of buckling MTs was determined by manually tracking the MT (Figure 5D).

Quantification of the Intensities of EB Comets

To obtain the intensity values of EB comets in Figure 7D, we collected the intensity profiles of mCherry-EB3 comets along several time points for each individual growth event by averaging across 6-pixel wide lines. The intensity profile for each time point was fitted to a Gaussian function with the background intensity (I_{BG}) to obtain the amplitude of the comet's peak I_A according to:

$$I_p(x,t) = I_{BG}(t) + I_A(t)e^{-\frac{(x-x_C)^2}{2\sigma^2}}$$

The final values were obtained by averaging the $I_A(t)$ for each individual growth event.

Tip-averaging of MT Intensity Profiles

To build average HyLite 488 tubulin intensity distribution at the growing tip (Figure 7K and 7L) we generated intensity profiles of 6 pixel thick line (400 nm) of 2-3 μm length with its middle point positioned approximately at the MT tip using Fiji (Schindelin et al., 2012) (similar to (Maurer et al., 2014)). Resulting profiles *I*(*x*) were fitted with the error function shifted in *x* using custom written MATLAB script:

$$I(x) = I_{BG} + \frac{1}{2} I_{AMP} \left(1 + \mathrm{erf}\left(\frac{x - x_c}{\sqrt{2}\sigma}\right)\right)$$

where fitting parameter I_{BG} corresponds to the intensity of background, I_{AMP} to the amplitude of the fluorescent signal, x_c to the position of the MT tip and σ to the degree of tip tapering convolved with microscope's point spread function (PSF) (see Figures S7H and S7J). Each profile was shifted by its x_c value, background subtracted with I_{BG} and normalized by I_{AMP} (Figures S7H and S7J).

Fitting and Averaging of GFP-TOG2-S Intensity Profiles

For simultaneous two color imaging of GFP-TOG2-S/rhodamine tubulin labeled MTs and GFP-TOG2-S/mCherry-EB3ΔTail we used OptoSplit III beamsplitter (Cairn Research Ltd, UK) equipped with double emission filter cube projecting two channels on the camera chip simultaneously. To account for chromatic aberrations of the objective, images of a calibration photomask with round 500 nm features positioned equidistantly with 2 μm (Compugraphics, UK) were acquired simultaneously in GFP and mCherry channels using transmitted bright-field illumination (Maurer et al., 2014). Based on feature detections we made sub-pixel channels alignment and non-linear registration using B-spline transform implemented in our Detection of Molecules ImageJ plugin and described earlier (version 1.1.5, https://github.com/ekatrukha/DoM_Utrecht, (Chazeau et al., 2016)). Registered videos were used to create kymographs by drawing segmented lines of 20 pixel width (1.25 μm) along growing MTs using KymoResliceWide plugin with maximum transverse intensity (http://fiji.sc/KymoResliceWide). Using kymograph images, we traced the growing MT tips with the "Segmented Line" ROI tool from ImageJ to mark the approximate position of the growing tip used for exact fitting later. The fitting of fluorescent intensity profiles was performed using a custom-written MATLAB script. Intensity profiles were extracted from kymographs at each time point corresponding with a range of ± 1μm from the approximate growing tip position marked earlier.

For the fitting of the rhodamine-labeled MT tip we used the complimentary error function:

$$I(x) = I_{BG} + \frac{1}{2} I_{AMP} \bullet \mathrm{erfc}\left(\frac{x - x_c}{\sqrt{2}\sigma}\right)$$

where the fitting parameter I_{BG} corresponds to the intensity of background, I_{AMP} to the amplitude of the fluorescent signal, x_c to the position of the MT tip and σ to the degree of tip tapering convolved with microscope's point spread function (PSF). For the averaging, each raw fluorescent profile was shifted by its x_c value, background subtracted with I_{BG} and normalized by I_{AMP}.

For the fitting of the mCherry-EB3ΔTail profile we assumed that the density distribution of EB3 in the comet decays exponentially from its maximum value close to the MT tip. To represent its convolution with microscope's PSF, we used a sum of the complimentary error function (lattice binding) and an exponentially modified Gaussian distribution:

$$I(x) = I_{BG} + \frac{1}{2} I_{lattice} \bullet \mathrm{erfc}\left(\frac{x - x_c}{\sqrt{2}\sigma}\right) +$$

$$+ \frac{1}{2} I_{EB} \bullet \exp\left(\frac{\lambda}{2}\left(\sigma^2 \lambda + 2(x - x_c)\right)\right) \bullet \left(1 - \mathrm{erf}\left(\frac{\sigma^2 \lambda + x - x_c}{\sqrt{2}\sigma}\right)\right)$$

where fitting parameter I_{BG} corresponds to the intensity of background, $I_{lattice}$ to the amplitude of the fluorescent intensity fraction associated with the MT lattice binding, I_{EB} to the amplitude of convolved exponential decay, x_c to the position of the maximum number of molecules in the molecules distribution (start of exponential decay position), σ to the PSF standard deviation and λ to the exponential decay constant. For the averaging, each raw fluorescent profile was shifted by its x_c value, background subtracted with I_{BG} and normalized by the maximum *I(x)* value.

For the fitting of the GFP-TOG2-S profile, we used a sum of the complimentary error function (lattice binding) and a Gaussian peak with standard deviation equal to microscopes point spread function (corresponding to the peak accumulation):

$$I(x) = I_{BG} + \frac{1}{2} I_{lattice} \bullet \mathrm{erfc}\left(\frac{x - x_c}{\sqrt{2}\sigma}\right) + I_{peak} \bullet exp\left(-\frac{1}{2}\left(\frac{x - x_c - x_{peak}}{\sigma_{PSF}}\right)^2\right)$$

where fitting parameter I_{BG} corresponds to the intensity of background, $I_{lattice}$ to the amplitude of the fluorescent intensity fraction associated with the lattice binding, I_{peak} to the amplitude of peak accumulation, x_c to the position of the lattice binding tip and σ to the degree of tip tapering, x_{peak} to the position of peak accumulation with respect to the lattice tip and fixed parameter σ_{PSF} is equal to the PSF standard deviation (1.5 px = 97.5 nm). For the averaging, each raw fluorescent profile was background subtracted with I_{BG}, normalized by $I_{lattice}$ and shifted by x_c value of either rhodamine-tubulin or mCherry-EB3ΔTail.

After normalization and alignment, all profiles corresponding to specific condition were linearly interpolated with the same step size of half-pixel (32.5 nm) and averaged for each individual kymograph. The final profiles (Figures 6H, 6I, and S5G) represent averages among several average kymograph profiles.

To calculate MT tip to lattice intensity ratio for GFP-TOG2-S we took into account differences in the convolution of step function (lattice) and delta-function (point-peak accumulation) using following formula:

$$ratio = \frac{I_{peak}\sqrt{2\pi}\sigma_{PSF}}{I_{lattice}}$$

where $I_{lattice}$ and I_{peak} are fitted values and σ_{PSF} is equal to the PSF standard deviation.

To find segments with continuous average speed of growth we decomposed fitted time sequences $x_c(t)$ of rhodamine-tubulin and mCherry-EB3ΔTail by piecewise linear approximations. It was done using multiscale trend analysis Matlab code (https://github.com/ekatrukha/MTA) based on (Zaliapin et al., 2005).

End Tapering Simulations

Monte-Carlo simulations of MTs tips were performed using 13 element- (protofilament-) wide regular array with 8 nm longitudinal distance between dimers as a MT lattice representation (Figure S7F). Absent length of *N* protofilaments was constant and defined by parameter *d* for Model A. For Model B it was randomly sampled from an exponential distribution with parameter *d*. Present tubulin dimers were labeled with a probability equal to the fraction of labeled tubulin in the corresponding experiment (0.09). Only labeled dimers were assumed to generate intensity profile in molecule number (Figure S7F) and its version convoluted with PSF of used microscope (Figure S7G). The PSF was approximated with a Gaussian function with the standard deviation of 122 nm. Convolved intensity values were binned together according to the image pixel size (65 nm) and the Gaussian noise was added leading to the signal-to-noise ratio of 7 observed in experiment (Figure S7I). Noise containing profiles were fitted and normalized using the same procedure as described in the previous section. For a single iteration of simulation, we used the same number of MTs as in the corresponding experimental condition with lengths determined from the fitting. A total of 50 iterations were run and averaged for each parameters combination of *N* and *d*. Residual between simulated and experimental profiles was calculated as a sum of squared differences using only those pixels in *x* which contain all individual profiles. The final fitting result was obtained by varying *N* and *d* independently in a search for a minimal residual value (Figure 7M).

Analysis of MT Growth Variability

Time lapse images of growing MTs labeled with HiLyte 488 tubulin were recorded at intervals of 0.7 s for 5 min and 350-400 ms of exposure time. To estimate the position of the MT plus end, we fitted MT intensity profiles as described above. Subsequently, the change in MT length over time was calculated as $\Delta L(t) = L(t) - L(\textit{first frame})$. Next, as described previously (Gardner et al., 2011a), we calculated the average mean squared displacement (MSD) of the MT length increments $\Delta L^2(\tau)$ for increasing values of time delay τ. We then fitted $\Delta L^2(\tau)$ to an MSD equation containing diffusion with drift:

$$\langle \Delta L^2 \rangle(\tau) = v_g^2\tau^2 + 2D_p\tau + \sigma_{err}^2$$

where τ corresponds to delay, v_g is the average speed of growth, D_p is the effective diffusion coefficient for the MT polymerization and σ_{err} is the experimental error.

Single-molecule Fluorescence Intensity Analysis of CLASP2α

Diluted protein samples of GFP, GFP-MACF43-LZ (Honnappa et al., 2009) and GFP-CLASP2α were immobilized in adjacent flow chambers of the same plasma cleaned glass coverslip as described previously (Sharma et al., 2016). The flow chambers were washed with MRB80 buffer and sealed with vacuum grease and immediately imaged with a TIRF microscope. 10-20 images of previously unexposed coverslip areas were acquired with 100 ms exposure time and low laser power. GFP, GFP-MACF43LZ and GFP-CLASP2α were located in different chambers of the same coverslip, so the same imaging conditions could be preserved. Single molecule fluorescence spots were detected and fitted with 2D Gaussian function using custom written ImageJ plugin DoM_Utrecht (https://github.com/ekatrukha/DoM_Utrecht). The fitted peak intensity values were used to build fluorescence intensity histograms.

CLASP Molecule Counting at MT Tips and Rescue Points

To determine the number of molecules of CLASP2α at a MT tip, we immobilized single molecules of CLASP2α onto the coverslip of one of the flow chambers and performed the *in vitro* reconstitution assay in the adjacent chamber of the same coverslip as described previously (Sharma et al., 2016). Images of unbleached CLASP2α single molecules were acquired first and using the same imaging/illumination conditions, time lapse imaging was performed with the *in vitro* assay with CLASP2α at 3 nM or 30 nM, using 100 ms exposure and 2 s intervals for 5 minutes. The plus end localized CLASP2α molecules or the molecules present at the rescue site were manually located in each frame and fitted with 2D Gaussian, the amplitude of which was used for the intensity analysis. For CLASP2α at the rescue site, 2-3 frames after rescue initiation were used to get the intensity values. To build the distributions of CLASP2α molecule numbers at the MT tip, each CLASP2α intensity value at the MT plus end or a rescue site was normalized by the average CLASP2α single molecule intensity from the adjacent chamber.

QUANTIFICATION AND STATISTICAL ANALYSIS

Kymographs were generated using the ImageJ plugin KymoResliceWide (http://fiji.sc/KymoResliceWide). MT dynamics parameters were determined from kymographs using an optimized version of the custom made JAVA plug in for ImageJ as described previously (Montenegro Gouveia et al., 2010; Sharma et al., 2016; Taylor, 1997). ~100-200 MT growth events were analyzed per condition.

The relative standard error for catastrophe frequency was calculated as described previously (Mohan et al., 2013). The relative standard error of mean rescue frequency was calculated in the same way as the standard error of the mean catastrophe frequency, i.e. $SE_r = \bar{f}_r \frac{SE_{t_{sh}}}{\bar{t}_{sh}}$, where $\bar{f}_r$, $\bar{t}_{sh}$ are average values and SE_{f_r}, $SE_{t_{sh}}$ are standard errors of rescue frequency and shortening time respectively. When the number of observed rescue events was relatively small (number of rescues ≤ 10) as compared to the catastrophes, we assumed that they follow a Poisson distribution. The standard deviation of the rescue frequency was calculated as the square root of its mean value and the standard error was calculated according to $SE_{f_r} = \frac{\sqrt{\bar{f}_r}}{\sqrt{N_r}}$, where $\bar{f}_r$ and SE_{f_r} are the average and the standard error of the rescue frequency and N_r is the number of rescues.

Statistical comparison between the different conditions was performed with Mann Whitney U test using GraphPad Prism 7.

DATA AND SOFTWARE AVAILABILITY

Figure 3F/ Figure S3E/ Table S1

The CLASP2-TOG1 structure has been deposited in the RCSB PDB (www.rcsb.org) under the PDB code 5NR4.

Developmental Cell, Volume *46*

Supplemental Information

CLASP Suppresses Microtubule Catastrophes

through a Single TOG Domain

Amol Aher, Maurits Kok, Ashwani Sharma, Ankit Rai, Natacha Olieric, Ruddi Rodriguez-Garcia, Eugene A. Katrukha, Tobias Weinert, Vincent Olieric, Lukas C. Kapitein, Michel O. Steinmetz, Marileen Dogterom, and Anna Akhmanova

Figure S1

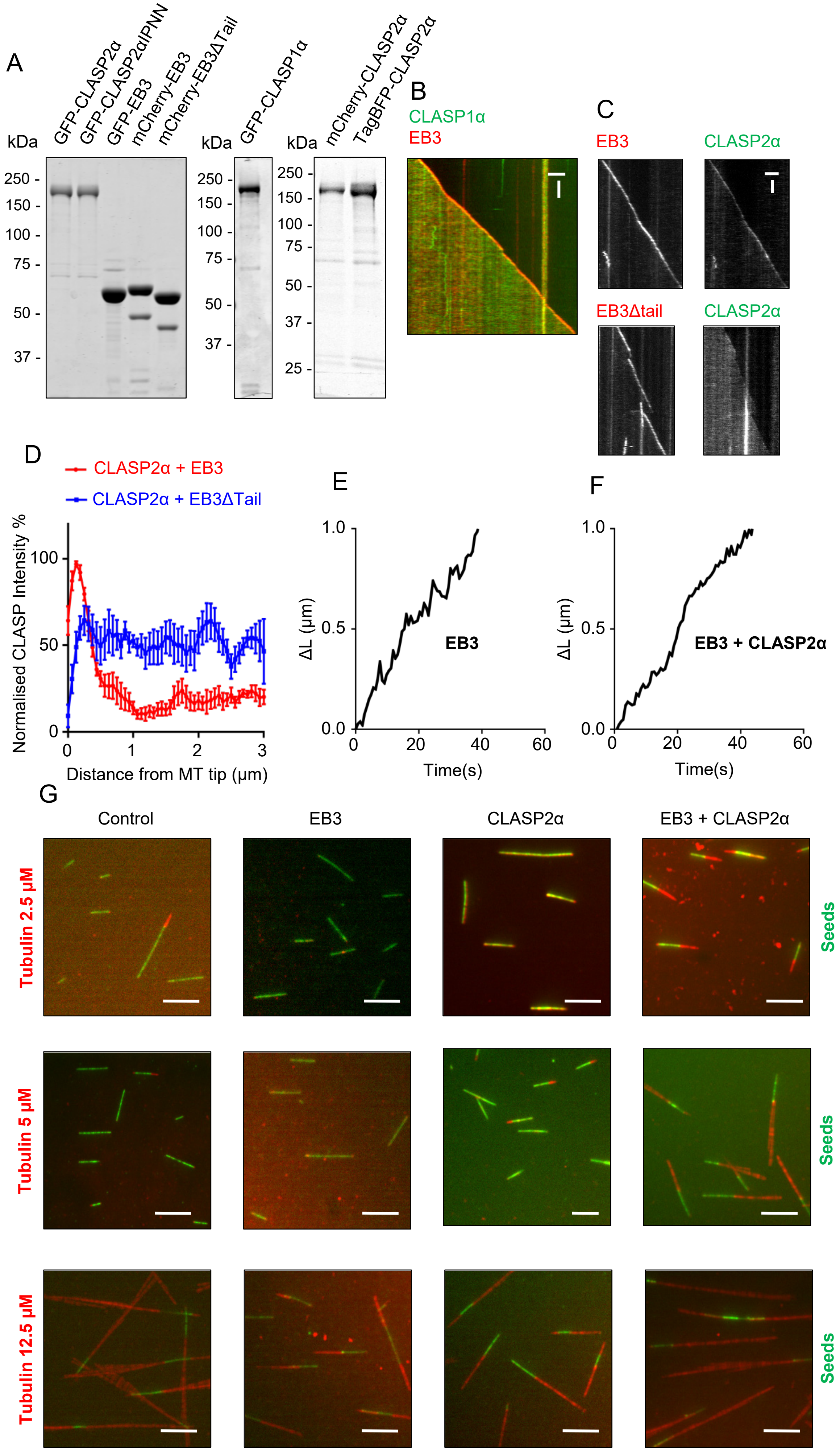
A
GFP-CLASP2α
GFP-CLASP2αIPNN
GFP-EB3
mCherry-EB3
mCherry-EB3ΔTail
GFP-CLASP1α
mCherry-CLASP2α
TagBFP-CLASP2α
kDa
250
150
100
75
50
37
25
B
CLASP1α
EB3
C
EB3
CLASP2α
EB3Δtail
CLASP2α
D
CLASP2α + EB3
CLASP2α + EB3ΔTail
Normalised CLASP Intensity %
Distance from MT tip (μm)
E
EB3
ΔL (μm)
Time(s)
F
EB3 + CLASP2α
ΔL (μm)
Time(s)
G
Control
EB3
CLASP2α
EB3 + CLASP2α
Tubulin 2.5 μM
Tubulin 5 μM
Tubulin 12.5 μM
Seeds

Figure S1, related to Figure 1. CLASPs suppress catastrophes and promote MT outgrowth from GMPCPP-stabilized seeds.

(A) Coomassie blue stained gels with GFP, mCherry and TagBFP-fusions of CLASP2α, GFP-fusions of the SxIP mutant CLASP2α, EB3 and mCherry-fusions of EB3 and EB3ΔTail. All CLASP proteins were purified from HEK293T cells whereas the EB3 proteins were purified from *E. coli*.

(B) Kymograph showing MT plus end dynamics in the presence of 20 nM mCherry-EB3 and 30 nM GFP-CLASP1α. Scale bars: 2 µm (horizontal) and 60 s (vertical).

(C, D) Kymographs illustrating MT plus end growth for MTs grown in the presence of 30 nM GFP-CLASP2α and 20 nM mCherry-EB3 or 300 nM GFP-CLASP2α and 20 nM mCherry-EB3Δtail (C) and normalized mean fluorescence intensity profiles along a growing MT plus end in the CLASP channel in the same conditions (D). Scale bars: 2 µm (horizontal) and 30 s (vertical). 8 MT profiles were used for averaging.

(E, F) Individual time traces of MT tip position in the presence of 20 nM mCherry-EB3 alone (E) or together with 30 nM GFP-CLASP2α (F).

(G) Maximum intensity projections of MT outgrowth (red) from HiLyte488-labeled GMPCPP seeds (green) in the presence of 2.5 µM, 5 µM and 12.5 µM rhodamine-labeled tubulin alone or together with 200 nM GFP-EB3 or 100 nM GFP-CLASP2α or both. Scale bar: 5 µm.

Figure S2

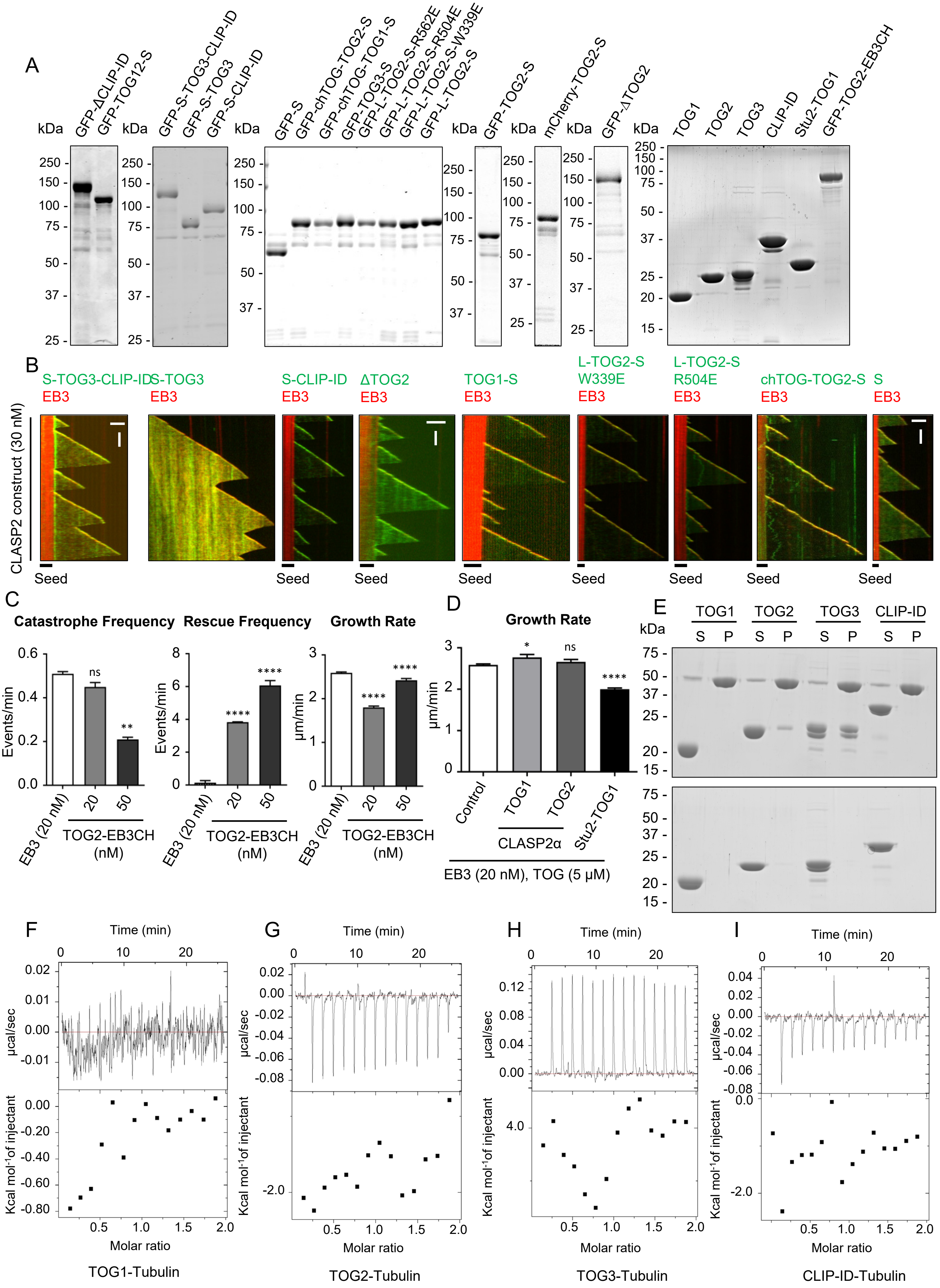

A
kDa
GFP-ΔCLIP-ID
GFP-TOG12-S
GFP-S-TOG3-CLIP-ID
GFP-S-TOG3
GFP-S-CLIP-ID
GFP-S
GFP-chTOG-TOG2-S
GFP-chTOG-TOG1-S
GFP-TOG3-S
GFP-L-TOG2-S-R562E
GFP-L-TOG2-S-R504E
GFP-L-TOG2-S-W339E
GFP-L-TOG2-S
GFP-TOG2-S
mCherry-TOG2-S
GFP-ΔTOG2
TOG1
TOG2
TOG3
CLIP-ID
Stu2-TOG1
GFP-TOG2-EB3CH
B
S-TOG3-CLIP-ID
S-TOG3
S-CLIP-ID
ΔTOG2
TOG1-S
L-TOG2-S W339E
L-TOG2-S R504E
chTOG-TOG2-S
S
EB3
CLASP2 construct (30 nM)
Seed
C
Catastrophe Frequency
Rescue Frequency
Growth Rate
Events/min
µm/min
EB3 (20 nM)
TOG2-EB3CH (nM)
ns
D
Growth Rate
Control
TOG1
TOG2
Stu2-TOG1
CLASP2α
EB3 (20 nM), TOG (5 µM)
E
TOG1
TOG2
TOG3
CLIP-ID
S
P
F
G
H
I
Time (min)
µcal/sec
Kcal mol-1of injectant
Molar ratio
TOG1-Tubulin
TOG2-Tubulin
TOG3-Tubulin
CLIP-ID-Tubulin

Figure S2, related to Figure 2. TOG2 domain of CLASP2α is necessary and sufficient for catastrophe suppression.

(A) Coomassie blue stained gels with GFP-fusions of full-length CLASP2α, TOG domain deletions, SxIP-fusions of different individual TOG domains, point mutants or chimeras with SxIP of CLASP2α fused to chTOG-TOG domains and mCherry-fusion of TOG2-S purified from HEK293T cells and the individual TOG domains of CLASP2α, STU2-TOG1 and the GFP-fusion of CLASP2α-TOG2 domain fused to EB3-CH domain purified from *E. coli*.

(B) Kymographs showing MT plus end dynamics in the presence of 20 nM mCherry-EB3 and 30 nM GFP-fusions of the indicated CLASP2α-TOG domain combinations, point mutants or SxIP motif of CLASP2α fused to chTOG-TOG domain or alone. Scale bars: 2 μm (horizontal) and 60 s (vertical).

(C) Parameters of MT plus end dynamics in the presence of mCherry-EB3 or GFP-TOG2-EB3CH at the indicated concentrations. Number of growth events analyzed: mCherry-EB3 alone, n= 207, GFP-TOG2-EB3CH (20 nM), n=194, GFP-TOG2-EB3CH (50 nM), n=173. Error bars represent SEM. For all plots, $^{**}p<0.05$, $^{****}p<0.0001$ and ns, no significant difference with control, Mann-Whitney U test.

(D) MT plus end growth rates in the presence of 20 nM mCherry-EB3 alone or together with 5 μM of CLASP2α-TOG1 or TOG2 domains or with Stu2-TOG1 domain. Error bars represent SEM. $^{*}p<0.05$, $^{****}p<0.0001$ and ns, no significant difference with control, Mann-Whitney U test.

(E) MT pelleting assays with CLASP2α-TOG1, TOG2, TOG3 and the CLIP-interacting domains with taxol-stabilized MTs.

(F-I) ITC analysis of the interaction between tubulin and the CLASP2α-TOG1, TOG2, TOG3 or the CLIP-interacting domain. No interactions were detected.

Figure S3

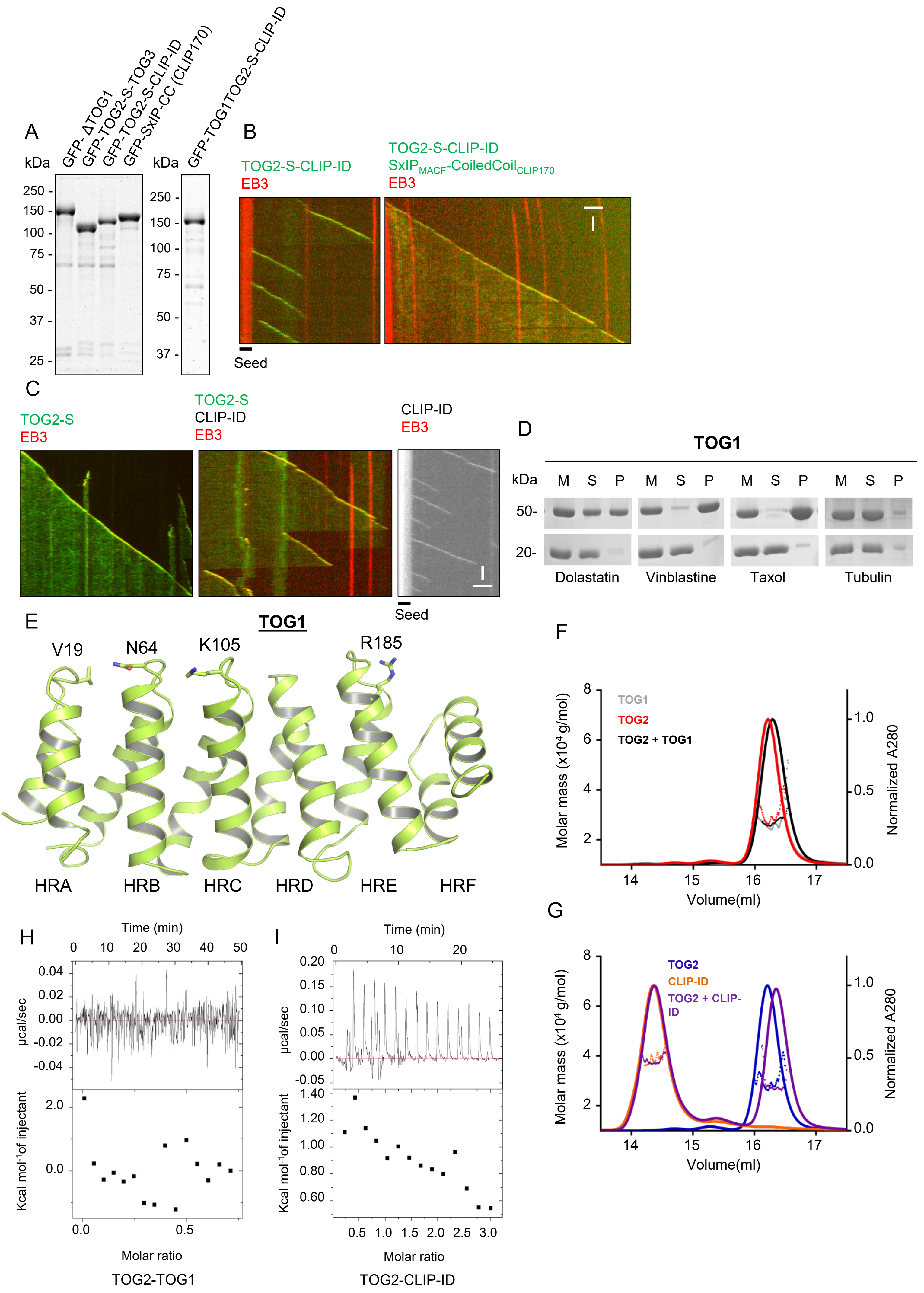

A
kDa
GFP- ΔTOG1
GFP-TOG2-S-TOG3
GFP-TOG2-S-CLIP-ID
GFP-SxIP-CC (CLIP170)
GFP-TOG1TOG2-S-CLIP-ID
250
150
100
75
50
37
25
B
TOG2-S-CLIP-ID
EB3
TOG2-S-CLIP-ID
SxIPMACF-CoiledCoilCLIP170
EB3
Seed
C
TOG2-S
EB3
TOG2-S
CLIP-ID
EB3
CLIP-ID
EB3
Seed
D
TOG1
kDa
M S P
50-
20-
Dolastatin
Vinblastine
Taxol
Tubulin
E
TOG1
V19
N64
K105
R185
HRA
HRB
HRC
HRD
HRE
HRF
F
TOG1
TOG2
TOG2 + TOG1
Molar mass (x10⁴ g/mol)
Normalized A280
Volume(ml)
G
TOG2
CLIP-ID
TOG2 + CLIP-ID
Molar mass (x10⁴ g/mol)
Normalized A280
Volume(ml)
H
Time (min)
µcal/sec
Kcal mol⁻¹of injectant
Molar ratio
TOG2-TOG1
I
Time (min)
µcal/sec
Kcal mol⁻¹of injectant
Molar ratio
TOG2-CLIP-ID

Figure S3, related to Figure 3. TOG1 domain of CLASP2α has an autoregulatory function and does not interact with MTs or tubulin oligomers.

(A) Coomassie blue stained gels with the indicated GFP-fusions, purified from HEK293T cells.

(B,C) Kymographs showing MT plus end dynamics in the presence of 20 nM mCherry-EB3 and together with the indicated proteins. 30 nM GFP-CLASP fusions, 30 nM of the MACF-CLIP-170 coiled coil fusion and 500 nM CLIP-ID were used. Scale bars: 2 μm (horizontal) and 60 s (vertical).

(D) MT pelleting of CLASP2α-TOG1 domain with tubulin-dolastatin rings, tubulin-vinblastine spirals, taxol-stabilized MTs or tubulin alone, as indicated.

(E) Crystal structure of the CLASP2α-TOG1 domain with the indicated HEAT repeats and the residues at the intra-HEAT loop regions.

(F, G) Analysis of the interactions between tubulin CLASP2α-TOG1 and TOG2, and TOG2 and CLIP-ID domains by size exclusion chromatography followed by multi-angle light scattering (SEC-MALS). No interactions were detected.

(H,I) ITC analysis of the interactions between CLASP2α TOG1 and TOG2 (H), and TOG2 and CLIP-ID (I). No interactions were detected in both the cases.

Figure S4

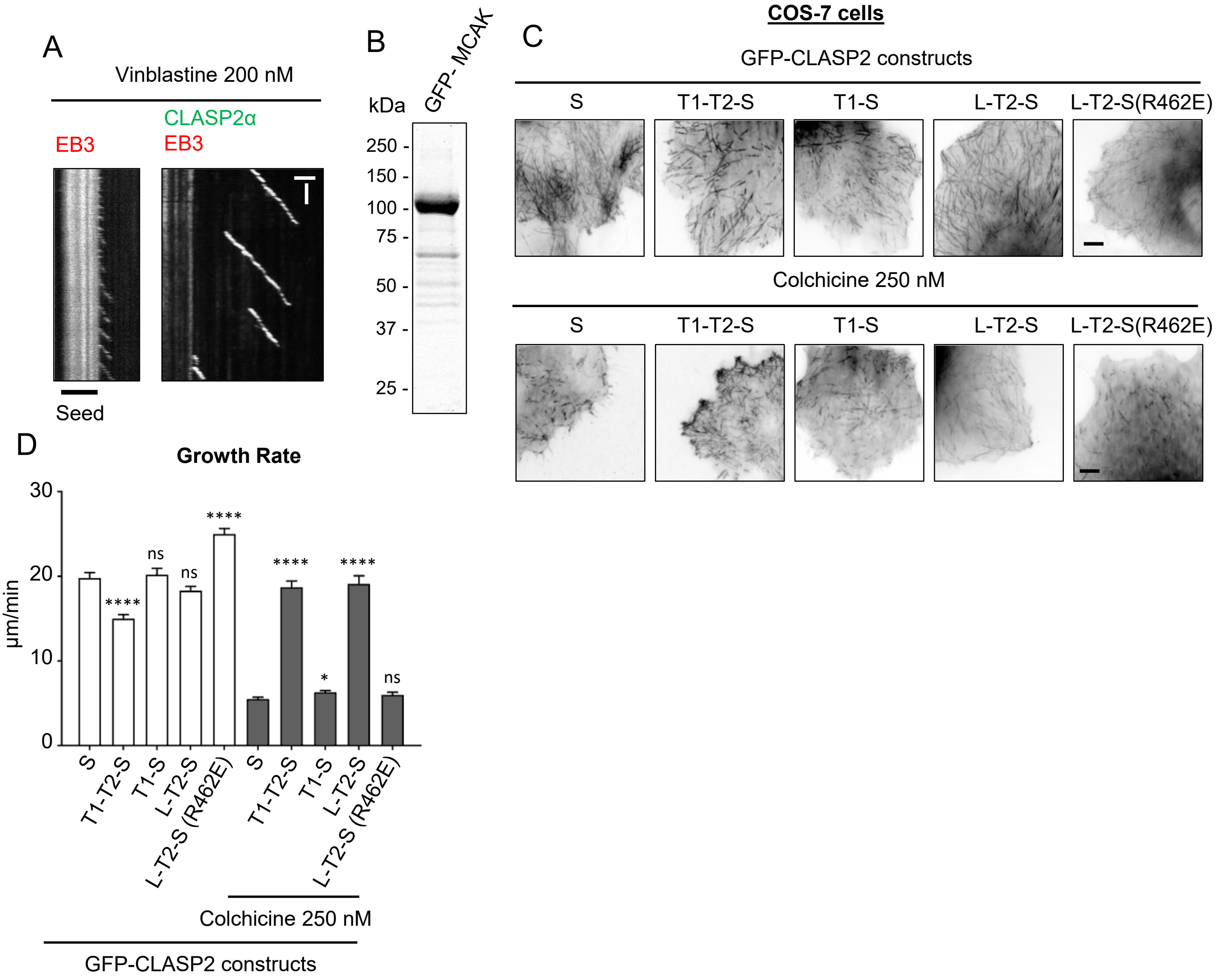
A
Vinblastine 200 nM
EB3
CLASP2α
EB3
Seed
B
GFP- MCAK
kDa
250
150
100
75
50
37
25
C
COS-7 cells
GFP-CLASP2 constructs
S
T1-T2-S
T1-S
L-T2-S
L-T2-S(R462E)
Colchicine 250 nM
S
T1-T2-S
T1-S
L-T2-S
L-T2-S(R462E)
D
Growth Rate
µm/min
30
20
10
0
****
ns
ns
****
****
*
****
ns
S
T1-T2-S
T1-S
L-T2-S
L-T2-S (R462E)
S
T1-T2-S
T1-S
L-T2-S
L-T2-S (R462E)
Colchicine 250 nM
GFP-CLASP2 constructs

Figure S4, related to Figure 4. CLASP2α suppresses catastrophes induced by MT-depolymerizing agents.

(A) Kymographs showing MT plus end dynamics in the presence of 20 nM mCherry-EB3 together with either 200 nM vinblastine alone or 30 nM GFP-CLASP2α. Scale bars: 2 μm (horizontal) and 60 s (vertical).

(B) Coomassie blue stained gel with GFP-MCAK.

(C) Maximum intensity projections illustrating time lapse imaging of COS-7 cells expressing GFP fusions of different TOG domain combinations of CLASP2α or a TOG2 point mutant fused to the SxIP motif of CLASP2α; cells were untreated or treated with 250 nM colchicine. Scale bar: 5 μm.

(D) MT plus end growth rates in COS-7 cells shown in (C). *$p<0.05$, ****$p<0.0001$ and ns, no significant difference with control, Mann-Whitney U test.

Figure S5

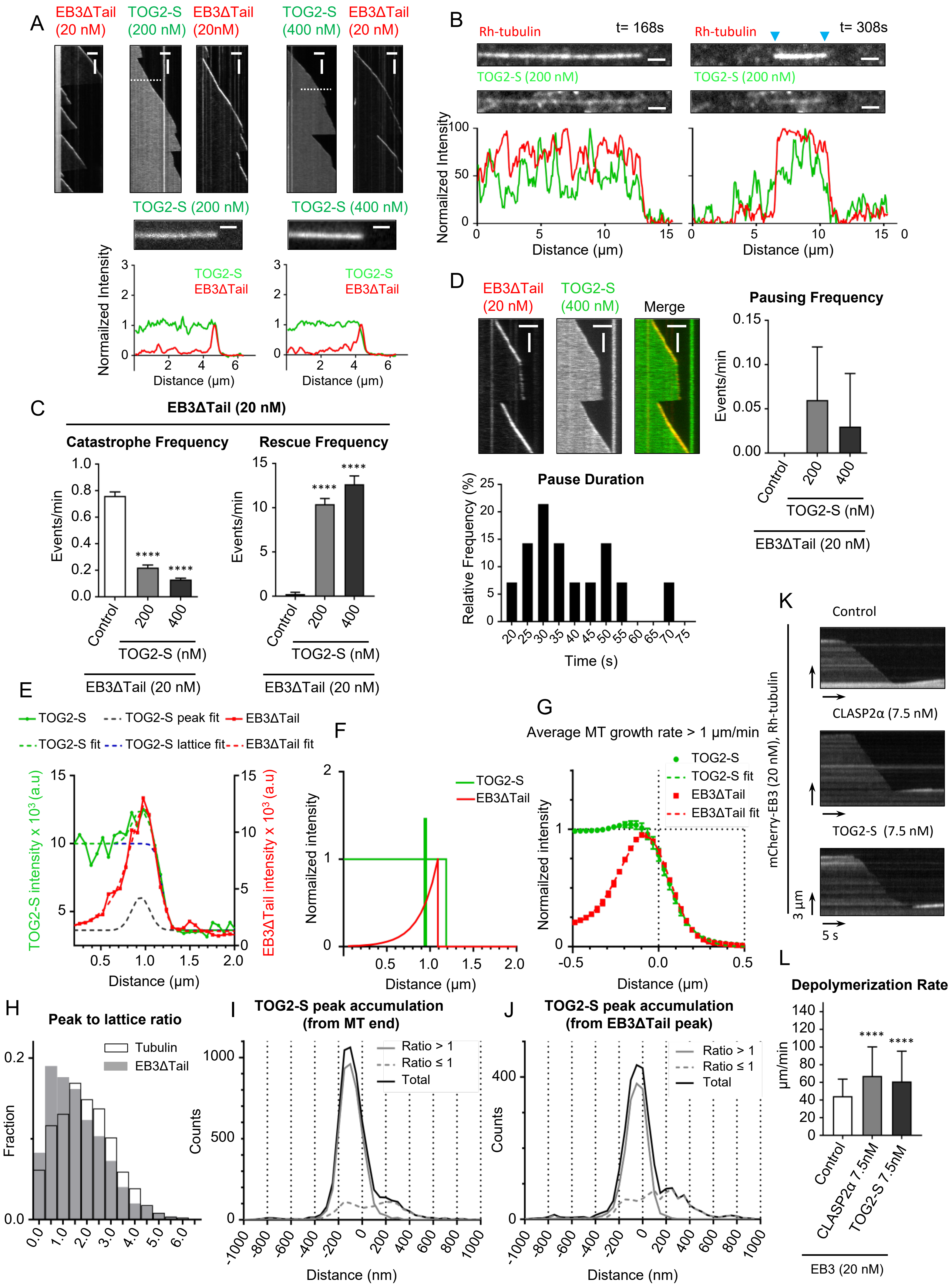
A
EB3ΔTail (20 nM)
TOG2-S (200 nM)
EB3ΔTail (20nM)
TOG2-S (400 nM)
EB3ΔTail (20 nM)
TOG2-S (200 nM)
TOG2-S (400 nM)
Normalized Intensity
Distance (μm)
TOG2-S
EB3ΔTail
B
Rh-tubulin
t= 168s
t= 308s
TOG2-S (200 nM)
Normalized Intensity
Distance (μm)
C
EB3ΔTail (20 nM)
Catastrophe Frequency
Rescue Frequency
Events/min
Control
200
400
TOG2-S (nM)
EB3ΔTail (20 nM)
D
EB3ΔTail (20 nM)
TOG2-S (400 nM)
Merge
Pausing Frequency
Events/min
Pause Duration
Relative Frequency (%)
Time (s)
E
TOG2-S
TOG2-S peak fit
EB3ΔTail
TOG2-S fit
TOG2-S lattice fit
EB3ΔTail fit
TOG2-S intensity x 10³ (a.u)
EB3ΔTail intensity x 10³ (a.u)
Distance (μm)
F
Normalized intensity
G
Average MT growth rate > 1 μm/min
H
Peak to lattice ratio
Tubulin
Fraction
I
TOG2-S peak accumulation (from MT end)
Ratio > 1
Ratio ≤ 1
Total
Counts
Distance (nm)
J
TOG2-S peak accumulation (from EB3ΔTail peak)
K
Control
CLASP2α (7.5 nM)
TOG2-S (7.5 nM)
mCherry-EB3 (20 nM), Rh-tubulin
3 μm
5 s
L
Depolymerization Rate
μm/min
Control
CLASP2α 7.5nM
TOG2-S 7.5nM
EB3 (20 nM)

Figure S5, related to Figure 6. TOG2 shows preference for a MT region behind the outmost MT end.

(A) Kymographs for 20 nM mCherry-EB3ΔTail alone or together with 200 nM GFP-TOG2-S or with 400 nM GFP-TOG2-S, stills and fluorescence intensity profiles for GFP-TOG2-S at the indicated concentrations (200 nM and 400 nM) in the presence of 20 nM mCherry-EB3ΔTail. For stills, scale bar: 1 µm. Scale bars for kymographs: 3 µm (horizontal) and 60 s (vertical).

(B) Stills and fluorescence intensity profiles for GFP-TOG2-S (200 nM) for a microtubule grown in the presence of rhodamine-tubulin post tubulin washout with the time after tubulin washout indicated. Blue arrowheads indicate the depolymerizing MT ends. Scale bar: 2 microns.

(C) Parameters of MT plus end dynamics in the presence of 20 nM mCherry-EB3ΔTail (n=187) in combination with GFP-TOG2-S at 200 nM (n=110) and 400 nM (n=105). Error bars denote SEM. For all plots, ****$p<0.0001$ and ns, no significant difference with control, Mann-Whitney U test.

(D) Kymographs showing pausing induced by GFP-TOG2-S (400 nM) in the presence of 20 nM mCherry-EB3ΔTail. Scale bar: 2 µm (horizontal) and 30s (vertical). Distributions of the pause durations (200 nM GFP-TOG2-S) are shown in the bottom panel, and the pausing frequency in the presence of 20 nM mCherry-EB3ΔTail alone or in combination with 200nM and 400 nM of GFP-TOG2-S (n=14 and n=9 in a total growth time of 255.5 and 291.9 minutes respectively) is shown in the right panel. Error bars denote SEM.

(E) Example of an individual fitting of GFP-TOG2-S and mCherry-EB3ΔTail fluorescence intensity profiles at GFP-TOG2-S (400 nM) and mCherry-EB3ΔTail (20 nM). Blue dashed line corresponds to the lattice and black dashed line to the peak accumulation components of overall TOG2-S fit function (green dashed line).

(F) Density distribution of TOG2-S (peak and lattice components shown separately, green lines) and EB3ΔTail (exponential decay, red line) extracted from the fitting shown in (E).

(G) Normalized, aligned, resampled and averaged fluorescence intensity profiles of GFP-TOG2-S (400 nM) and mCherry-EB3ΔTail (20 nM) with the average MT growth rate above 1 μm/min. Profiles were first averaged per kymograph. Error bars represent SEM of second averaging among multiple kymographs (10 kymographs, 2413 total time points).

(H) Histograms of ratios of the peak intensity to lattice intensity of GFP-TOG2-S (400 nM) measured on rhodamine-tubulin-labeled MTs (white bars, n=5997) or in the presence of mCherry-EB3ΔTail (20 nM) (grey bars, n=2774 time points).

(I) Histograms of the distances between TOG2-S intensity peak accumulation and the fitted position of MT tip (solid line, n=5997) for 400 nM GFP-TOG2-S. Grey line displays the histogram when TOG2-S tip to lattice intensity ratio is above 1 (n=4586) and dashed grey line when it is below 1 (n=1411). In the histograms, 0 corresponds to the fitted position of the MT end or EB3ΔTail peak.

(J) Histograms of distance between TOG2-S intensity peak accumulation and the fitted peak of EB3ΔTail (solid line, n=2774) for 400 nM GFP-TOG2-S and 20 nM mCherry-EB3Δtail. Grey line displays the histogram when TOG2-S tip to lattice intensity ratio is above 1 (n=1778) and dashed grey line when it is below 1 (n=996). In the histograms, 0 corresponds to the fitted position of the EB3ΔTail peak.

(K,L) Kymographs of depolymerizing MT ends in the presence of rhodamine-tubulin and mCherry-EB3 (20 nM) alone (n=160) or together with 7.5 nM GFP-CLASP2α (n=114) or 7.5 nM GFP-TOG2-S (n=151) (K) and the corresponding quantification of

depolymerization rates (L). Scale bar: 3 μm (vertical) and 5s (horizontal). Error bars denote SD. For depolymerization rates plot, ****$p<0.0001$, Mann-Whitney U test.

Figure S6

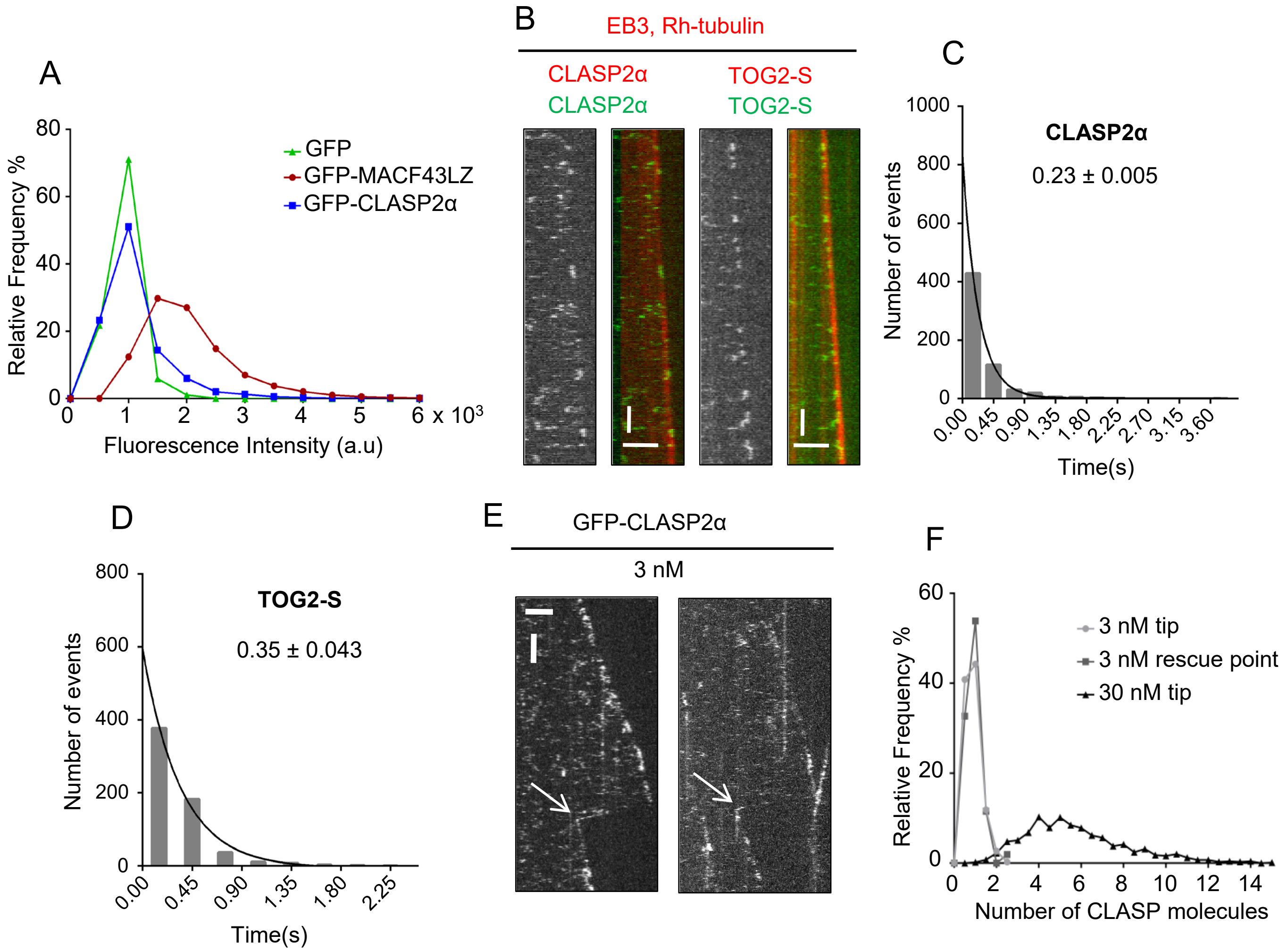
A
GFP
GFP-MACF43LZ
GFP-CLASP2α
Relative Frequency %
Fluorescence Intensity (a.u)
6 x 10³
B
EB3, Rh-tubulin
CLASP2α
CLASP2α
TOG2-S
TOG2-S
C
CLASP2α
0.23 ± 0.005
Number of events
Time(s)
D
TOG2-S
0.35 ± 0.043
Number of events
Time(s)
E
GFP-CLASP2α
3 nM
F
3 nM tip
3 nM rescue point
30 nM tip
Relative Frequency %
Number of CLASP molecules

Figure S6, related to Figure 7. A small number of CLASP2α molecules can suppress catastrophe and induce rescue.

(A) Distributions of fluorescence intensity values for single molecules of GFP (green, mean intensity value=0.92 x 10^3 ± 0.25 x 10^3) (n=846 molecules analyzed), GFP-CLASP2α (blue, mean=1.2 x 10^3 ± 1.3 x 10^3) (n=2341) and GFP-MACF43-LZ (red, mean=2.12 x 10^3 ± 1.5 x 10^3) (n=7516).

(B) Kymographs showing MT plus end and lattice turnover for GFP-CLASP2α and TOG2-S for MTs grown in the presence of 20 nM mCherry-EB3, rhodamine-tubulin together with both 30 nM mCherry-CLASP2α and 0.5 nM GFP-CLASP2α or 30 nM mCherry-TOG2-S and 0.5 nM GFP-TOG2-S. Scale bars: 2 μm (horizontal) and 2 s (vertical).

(C, D) Exponential fits of the distributions of dwell times at the MT tip for CLASP2α (mean time= 0.23 ± 0.005 s), n=608 tip events (C) and TOG2-S (mean time= 0.35 ± 0.043 s), n=622 tip events (D).

(E) Kymographs showing MT plus end dynamics in the presence of 3 nM GFP-CLASP2α and 20 nM mCherry-EB3 (only green channel shown). Scale bars: 2 μm (horizontal) and 30 s (vertical).

(F) Distributions of the number of GFP-CLASP2α molecules in the presence of 20 nM mCherry-EB3 at growing MT plus ends (n=1078 tip events) and at rescue points (n=17 rescue events) in the presence of 3 nM GFP-CLASP2α, and at growing MT plus end with 30 nM GFP-CLASP2α (n=1393 tip events).

Figure S7

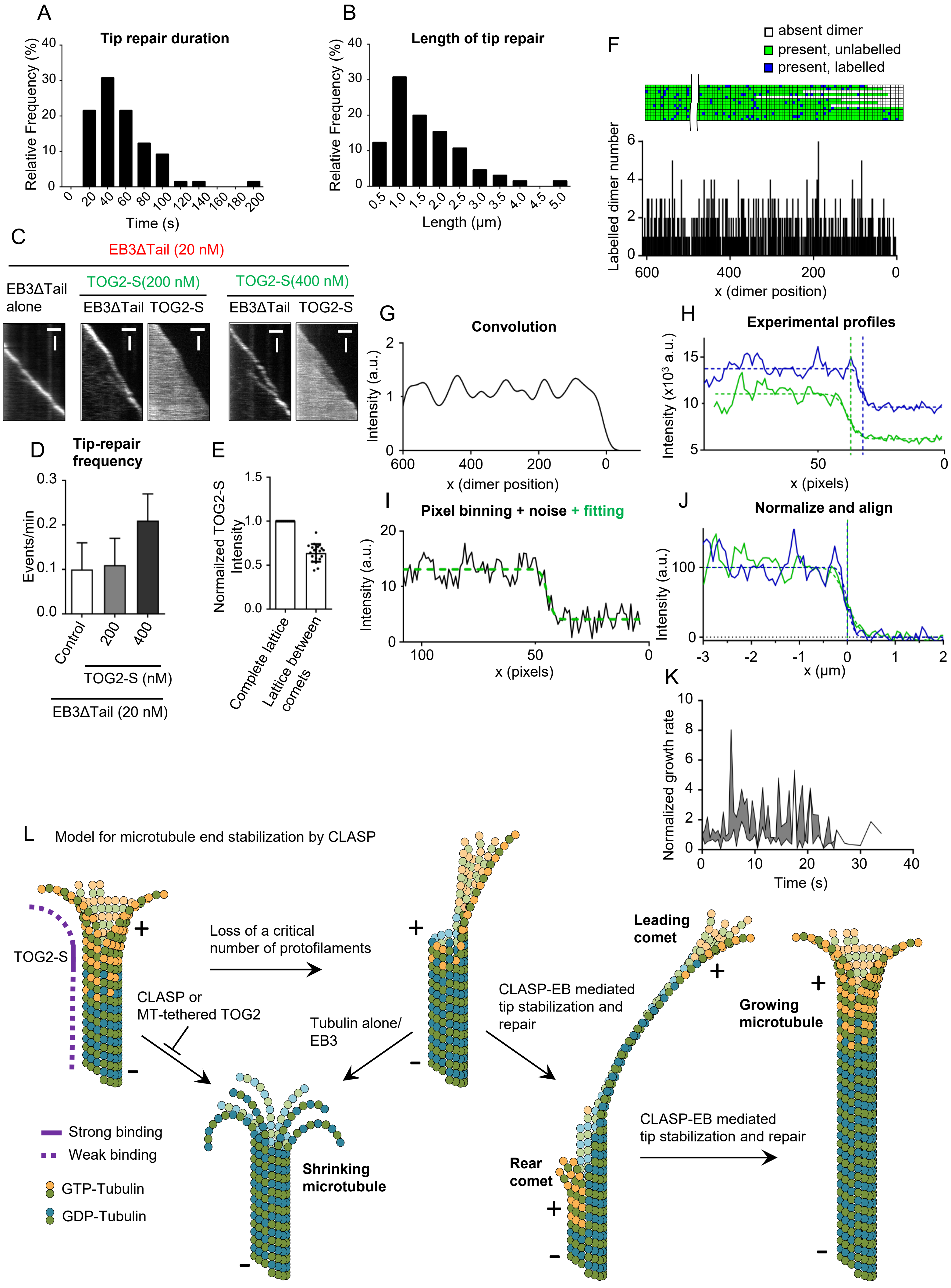
A
Tip repair duration
Relative Frequency (%)
Time (s)
B
Length of tip repair
Relative Frequency (%)
Length (µm)
F
absent dimer
present, unlabelled
present, labelled
Labelled dimer number
x (dimer position)
C
EB3ΔTail (20 nM)
EB3ΔTail alone
TOG2-S(200 nM)
EB3ΔTail TOG2-S
TOG2-S(400 nM)
EB3ΔTail TOG2-S
D
Tip-repair frequency
Events/min
Control
200
400
TOG2-S (nM)
EB3ΔTail (20 nM)
E
Normalized TOG2-S Intensity
Complete lattice
Lattice between comets
G
Convolution
Intensity (a.u.)
x (dimer position)
H
Experimental profiles
Intensity (x10³ a.u.)
x (pixels)
I
Pixel binning + noise + fitting
Intensity (a.u.)
x (pixels)
J
Normalize and align
Intensity (a.u.)
x (µm)
K
Normalized growth rate
Time (s)
L
Model for microtubule end stabilization by CLASP
TOG2-S
Loss of a critical number of protofilaments
CLASP or MT-tethered TOG2
Tubulin alone/ EB3
CLASP-EB mediated tip stabilization and repair
Leading comet
Growing microtubule
Rear comet
CLASP-EB mediated tip stabilization and repair
Shrinking microtubule
Strong binding
Weak binding
GTP-Tubulin
GDP-Tubulin

Figure S7, related to Figure 7. Characterization of MT tip repair events.

(A, B) Distributions of the duration (A) and length (B) of tip repair events, mean time=54 ± 4 s; mean length= 1.6 ± 0.1 microns from n=65 events. Error bars represent SEM.

(C) Kymographs showing tip repair events with EB3ΔTail alone and together with the indicated concentrations of TOG2-S (200 nM and 400 nM). Scale bars: 1 μm (horizontal) and 15 s (vertical).

(D) Frequency of tip repair for MTs grown in the presence of 20 nM mCherry-EB3ΔTail alone (n=24) or together with 200 nM or 400nM GFP-TOG2-S (n=27 and 62 respectively). The frequency was calculated by dividing the number of observed tip repair events by the total growth time, n=number of tip repair events observed in each condition. Error bars represent SEM.

(E) Averaged fluorescence intensity of GFP-TOG2-S in the MT lattice region between the leading and lagging comet, normalized to the average intensity along the complete MT lattice (n=21). Mean ± SD.

(F) Schematic view of MT structure used for Monte-Carlo simulations and an example of labeled dimer number distribution along MT length for a single random realization of simulation.

(G) Intensity profile after convolution of dimer number density from (F) with the point spread function of a microscope.

(H) Examples of experimentally measured MT tip intensity profiles (solid lines) and corresponding error function fittings (dashed lines), vertical lines mark a position of tip derived from the fitting.

(I) Intensity profile from (G) after binning in *x* with the experimental image pixel size and noise addition (black solid line) and corresponding error function fitting (green dashed line).

(J) Experimental intensity profiles from (H) (solid lines) and corresponding fitted curves (dashed lines) after background subtraction, alignment and normalization.

(K) Quantification of rear comet velocities over time during tip repair events corresponding to Fig 7N. Grey areas represent SEM.

(L) Model for MT end stabilization mediated by CLASP.

Table S1, related to Figure 3, S3. X ray crystallography data collection and refinement statistics.

**Data Collection**[a]	**Native**	**S-SAD**
Wavelength (Å)	1	2.066
Resolution range (Å)[b]	38.49 - 1.19 (1.24 - 1.19)	47.12 - 2.14 (2.2 - 2.14)
Space group	P 1 21 1	P 1 21 1
Unit cell a, b, c (Å) α, β, γ (°)	66.08, 47.12, 74.15, 90, 115.83, 90	66.05, 47.12, 74.02, 90, 115.797, 90
Total reflections	797853 (62543)	831552 (6204)
Unique reflections	128081 (11920)	21534 (1379)
Multiplicity	6.2 (5.1)	38.62 (4.50)
Completeness (%)	99 (95)	93.49 (60.91)
Mean I/sigma(I)	13.42 (0.84)	64.0 (10.2)
Wilson B-factor	12.78	-
R-merge	0.07116 (1.571)	0.056 (0.119)
R-meas	0.07762 (1.748)	0.056 (0.132)
CC1/2[c]	1 (0.3)	0.999 (0.984)
CC*	1 (0.679)	Chi orientations/oscillations :<br>Chi0/360°<br>Chi5/360°<br>Chi10/360°<br>Chi15/360°<br>Chi20/360°<br>Chi25/360°<br>Chi30/360°<br>Chi35/360°
**Refinement**		
R-work	0.2003 (0.2917)	
R-free	0.2137 (0.3135)	
Macromolecules	3368	
Water	770	
RMS(bonds) (Å)	0.008	
RMS(angles) (°)	1.27	
Ramachandran favored (%)[d]	99	
Ramachandran outliers (%)[d]	0	
**B-factors**		
Average B-factor	21.22	
Macromolecules	19.03	

Solvent	30.80

[a] Highest resolution shell statistics are in parentheses.

[b] Resolution cutoffs were chosen based on CC1/2 and Mean I/sigma(I) (Karplus and Diederichs, 2012)

[c] As defined by (Karplus and Diederichs, 2012)

[d] As defined by MolProbity (Davis et al., 2004)
